# Improved mental health outcomes and normalised spontaneous EEG activity in veterans reporting a history of traumatic brain injuries following participation in a psilocybin retreat

**DOI:** 10.3389/fpsyt.2025.1594307

**Published:** 2025-08-06

**Authors:** Grace Blest-Hopley, Giuseppe Pasculli, Simon G. D. Ruffell, WaiFung Tsang, Olateju Emmanuel, Kathryn M. Pate, Hannes Kettner, Leor Roseman, David Erritzoe, Robin Carhart-Harris

**Affiliations:** ^1^ King’s College London, Institute of Psychiatry, Psychology and Neuroscience, London, United Kingdom; ^2^ Department of Computer, Automatic and Management Engineering, Faculty of Information Engineering, Computer Science and Statistics (DIAG), Sapienza University of Rome, Rome, Italy; ^3^ R&D, Italian Society of Psychedelic Medicine (Societá Italiana di Medicina Psichedelica - SIMePsi), Bari, Italy; ^4^ Department of Psychology, University of Exeter Onaya Science, Iquitos, Peru; ^5^ Applied Artificial Intelligence and Robotics Research Laboratory, Department of Electronic and Electrical Engineering, Obafemi Awolowo University, Ife, Nigeria; ^6^ R&D, Coruna Medical LLC, Longmont, CO, United States; ^7^ Department of Medicine, Centre for Psychedelic Research, Imperial College London, London, United Kingdom; ^8^ Psychedelics Division, Neuroscape, University of California, San Francisco, San Francisco, United States; ^9^ Department of Psychology, University of Exeter, Exeter, United Kingdom

**Keywords:** psilocybin, traumatic brain injury, veterans, psychedelic therapy, mental health, PTSD, neuroplasticity, EEG

## Abstract

**Introduction:**

Psilocybin, a serotonergic psychedelic, has shown therapeutic potential in treating mental health disorders by, amongst the many effects, promoting neuroplasticity and reorganising functional connectivity across cortical and subcortical networks involved in emotion and cognition. Veterans with traumatic brain injuries (TBI) often experience chronic neurological and psychological symptoms such as post-traumatic stress disorder (PTSD) and depression. This study investigates the effects of psilocybin administered in retreat settings on veterans with a history of TBI, focusing on mental health outcomes and changes in brain connectivity as measured by EEG.

**Methods:**

A total of 21 participants were recruited through the Heroic Hearts Project, which facilitated access to two six-day psilocybin retreats in Jamaica. Before the retreat, participants underwent three individual and three group coaching sessions to prepare for the experience. During the retreat, two psilocybin ceremonies were held, spaced 48 hours apart. Participants received an initial dose of 1.5g to 3.5g of dried psilocybin mushrooms, with the option to increase the second dose up to 5g. Psilocybin was administered in a tea format, under the supervision of experienced facilitators. Psychological outcomes were assessed using validated questionnaires (PCL-5, PHQ-9, STAI) at baseline (four weeks pre-retreat) and four weeks post-retreat. Electroencephalography (EEG) was used to measure brainwave activity pre- and post-treatment. Paired t-tests were used to analyze changes in psychological scores, while EEG frequency band analysis assessed changes in brain function and connectivity.

**Results:**

Improvements were observed across several mental health measures: PTSD (PCL-5 scores decreased by 50%, p=0.010), depression (PHQ-9 scores decreased by 65%, p<0.001), and anxiety (STAI) scores decreased by 28%, p<0.001). EEG data showed decreased delta and theta power in frontal and temporal regions, indicating potential improvements in cognitive control and emotional processing. Enhanced coherence in alpha and beta bands suggested improved neural communication.

**Discussion:**

The study suggests that psilocybin retreats might provide improvements in psychological well-being and brain connectivity in veterans with TBI. Reduced delta power and normalised theta activity suggest better emotional regulation, while improved coherence in alpha and beta bands may reflect increased cognitive engagement. Further, these preliminary outcomes provide a potential rationale for the design and implementation of larger-scale, controlled studies to validate and expand upon these initial findings.

## Introduction

Psilocybin, the active ingredient in magic mushrooms, is a naturally occurring psychedelic substance that has garnered significant attention in recent years for its therapeutic potential in treating various mental health disorders (1). As a classic serotonergic psychedelic, psilocybin acts primarily on the serotonin (5-HT) 2A (5-HT2A) receptors in the brain, leading to a cascade of neurobiological effects that promote neuroplasticity (2) and may alleviate symptoms of mental health disorders such as depression, anxiety, and post-traumatic stress disorder (PTSD) (3). Mechanistically, psilocybin’s action at these receptors results in reduced amygdala reactivity during emotional processing and increased insightfulness and introspection, making it a promising candidate for therapeutic use (4, p. 20). These properties, working primarily through the 5-HT2A receptors, help in producing acute psychedelic effects that are beneficial for psychiatric and neurological treatments (5, 6).

Currently, psilocybin is being used in retreat settings, where participants undergo guided psychedelic experiences designed to facilitate psychological healing and personal growth (7). These retreats have shown promising outcomes, with significant behavioral changes (8). Clinical studies and anecdotal evidence suggest that psilocybin can serve as a potent therapeutic tool in mental health disorders, particularly when used in structured environments that provide comprehensive support and integration of the psychedelic experience (9, 10).

Veterans are particularly vulnerable to traumatic brain injury (TBI) due to their exposure to concussive forces during military service (11). These injuries, often resulting from physical impacts or close proximity to explosions (12), can lead to a range of chronic neurological and psychological issues (13). The pathology of head trauma involves complex interactions between physical brain damage and subsequent mental health challenges, including a high prevalence of conditions such as depression, anxiety, and PTSD (14). Furthermore, TBI is associated with headaches, dizziness, and impairments in memory, cognition, vision, sleep, and coordination (15–17). Electroencephalography (EEG) is a well-established tool for detecting electrophysiological abnormalities following TBI, including mild TBI (mTBI). Numerous studies have demonstrated that TBI leads to characteristic changes in EEG spectral power and connectivity that can persist into the chronic phase, even when structural neuroimaging is unremarkable (18–20).

### Delta and Theta Bands

Increased power in the delta (1–3.5 Hz) and theta (4–7.5 Hz) bands is among the most consistently reported EEG abnormalities in TBI patients. Lewine et al. (19) found that individuals with chronic mTBI exhibited significantly elevated global relative theta power compared to controls, as well as increased delta power at specific electrode sites. Notably, quantitative analyses revealed significantly increased (P < 0.025) absolute, or more commonly relative, delta or theta power for at least one electrode location in 20 of these cases (19). These slow-wave increases are thought to reflect diffuse or focal cortical dysfunction and have been linked to both acute and persistent post-concussive symptoms, with higher theta:alpha and theta:beta amplitude ratios, as well as increased slow wave quantity with slow wave abnormalities correlating with symptom severity (18, 20).

### Alpha Band

Alterations in alpha (8–12 Hz) activity are also documented. Lewine et al. reported that global relative alpha power was decreased for mTBI patients compared to controls (19). Another study has observed a transient post-traumatic slowing of the posterior alpha rhythm in the acute phase, followed by a gradual return to baseline over weeks to months (18), and reduced alpha power was considered a marker of impaired thalamocortical and cortical network function, with lower alpha power been associated with poorer functional outcomes in severe TBI (21).

### Beta Band

Beta (12.5–25 Hz) abnormalities are less frequently described but are nonetheless significant. Lewine et al. found that global beta-band interhemispheric coherence was decreased for mTBI patients (19). Additionally, a recent intracranial EEG study in TBI indicates that beta power is positively correlated with neurological recovery. In particular, improvement of Glasgow Coma Scale (GCS) during recovery coincided with the recovery of beta power over time, whereas beta power was usually persistently low in patients with poor neurological outcomes (22). These findings suggest that beta oscillations may serve as a marker for both injury severity and recovery trajectory.

Beyond spectral power, TBI is associated with disruptions in EEG coherence and functional connectivity, particularly in the slow-wave bands. For example, individuals with mTBI have shown significantly less EEG global coherence compared to control subjects while awake, and this reduction in coherence correlates with the severity of post-concussive symptoms (20). Lewine et al. also highlighted decreased interhemispheric coherence in the beta band as a distinguishing feature of mTBI (19).

Psilocybin’s therapeutic potential may extend to individuals with head trauma through its profound effects on behaviour, brain function, and potential neural recovery. Behaviourally, psilocybin induces significant mood shifts, reducing anxiety and enhancing emotional processing (23). Functionally, it disrupts maladaptive neural networks and promotes new, healthier patterns of connectivity, which can be particularly beneficial for repairing or rewiring damaged neural pathways in individuals with head trauma (24). Furthermore, psilocybin has been found to modulate synaptogenesis and neurogenesis and increase synaptic plasticity (25–28), offering a novel paradigm for neuronal recovery after TBI.

Residential psilocybin retreat programs offer a structured environment where veterans can receive comprehensive support before, during, and after their psychedelic experiences on-site, as well as before and after attending the retreat (29). Group dynamics play a critical role in these retreats, providing participants with a sense of community and shared understanding (30). Specialised programs for veterans address their unique needs, offering tailored support and integration strategies to maximise the therapeutic benefits of psilocybin for this population. Appropriate screening is necessary to ensure the suitability of those attending such a retreat; however, the safety profile of psilocybin is well-established, showing low physiological toxicity and no notable withdrawal symptoms, making it suitable for use in controlled settings (31, 32).

### Study rationale

Researching the outcomes of psilocybin retreat programs is crucial for expanding our understanding of psilocybin’s effects on behavioural, functional (measured using EEG), and cognitive measures in veterans with psychological difficulties and who have a history of TBI. Previous studies have shown that psychedelics can induce significant improvements in mental health outcomes in veterans (29, 33), but there is a need for more comprehensive and focused data on its effects in treating symptoms associated with head traumas.

Our hypothesis is that attending a residential psilocybin retreat will lead to measurable improvements in military veterans’ mental health, wellbeing, and neurological functioning. Mental health and wellbeing will be measured by a battery of behavioural questionnaires, that assess symptoms of PTSD, depression, anxiety, sleep, concussion related symptoms, wellbeing, quality of life and military to civilian connectedness.

Building on prior work characterising electrophysiological abnormalities in TBI—including elevated delta and theta power and attenuated alpha and beta rhythms (18–21)—we hypothesise that participation in a psilocybin-assisted retreat will elicit measurable changes in resting-state EEG activity. Specifically, we anticipate a reduction in delta (1–4 Hz) power, a frequency band often elevated in chronic TBI and associated with cortical dysfunction; this may reflect a shift toward more adaptive waking cognitive states, consistent with its role in neural repair and inhibition (34). We further predict increased coherence in the theta band (4–8 Hz), which has been linked to memory integration and emotional regulation (35). Alpha (8–12 Hz) and beta (12–30 Hz) activity—frequencies associated with thalamocortical function and cognitive processing (36, 37)—are expected to show increased power and coherence, reflecting improved attentional control and emotional stability. These changes are hypothesised to localise primarily to frontal and temporal regions, which are frequently implicated in TBI-related network dysfunction (38).

## Methods

This study employed a comprehensive, multidimensional approach to assess the therapeutic effects of psilocybin administered in retreat settings among combat veterans with a history of TBI and currently presenting with psychological distress.

### Participant recruitment

Participants were recruited through the Heroic Hearts Project network, who facilitated veterans who reported experiencing TBIs with access to psilocybin retreats provided by Beckley Retreats and geographically located in Jamaica. The retreats focused on veterans who reported having a history of TBI and served in roles with a higher risk for TBI; and those actively seeking to attend a psychedelic retreat program with Heroic Hearts Project. Potential participants to the retreat underwent a screening process based on inclusion and exclusion criteria defined by Heroic Hearts Project and Beckley Retreats. Inclusion criteria included a history of suspected chronic TBI and psychological distress, while exclusion criteria ensured participants had good general health, no history of heart problems, no diagnoses of psychotic disorders or personality disorders and no further physiological or mental health issues deemed unsuitable by retreat staff for participation in the retreat. Participants were asked to disclose any mental health conditions; however, a formal diagnosis was not a prerequisite for retreat participation. All potential participants were screened by the Heroic Heart Program team and medical staff, before being screened again for suitability by Beckley Retreats.

As part of this screening process, individuals taking contraindicated psychoactive medications (e.g., antidepressants, anxiolytics) were required to discontinue their use under physician supervision prior to attending the retreat. At the time of participation, no individuals were taking active contraindicated medications. Psychological therapy status was not formally documented, but participants were not engaged in ongoing therapy during the retreat itself. Medication and treatment history were reviewed and confirmed by both the Heroic Hearts medical screening team and Beckley Retreats staff.

Prior to participation, candidates completed three individual, and three group coaching sessions conducted remotely by trained coaches provided by Heroic Hearts Project. These sessions aimed to prepare participants for the retreat experience and facilitate group cohesion. Participants travelled to the retreat centres in Jamaica for a six-day long stay, where they engaged in two psilocybin ceremonies, each involving controlled doses of psilocybin. In total 2 6-day retreats were provided by Beckley Retreats at a location in Jamaica over an 8 month period.

### Data collection

Data collection was structured to capture a wide range of psychological, behavioural, cognitive, and neurophysiological measures. The study used a combination of digital platforms for demographic and psychological questionnaires (Alchemer^1^) and EEG conducted at the retreat centre.

### Demographic, medical, and medication history

Participants provided comprehensive demographic information, including vocational history and self-reported medical history. This baseline data collection was conducted remotely from four weeks before the retreat.

### Psychological and behavioral measures

Several validated questionnaires were used to assess various mental health and wellbeing outcomes:

1. PTSD Checklist (PCL-5) (39)
2. Patient Health Questionnaire (PHQ-9) for depression (40)
3. State-Trait Anxiety Inventory (STAI) (41)
4. Rivermead Post-Concussive Questionnaire (RPQ) (42)
5. Quality of Life After Brain Injury – Overall Scale (Qolibri-OS) (43)
6. PROMIS Sleep Disturbance Short Form (PROMIS-SD) (44)
7. The Warwick – Edinburgh Mental Well-being Scale (W-EMWS) (45)
8. Military to civilian questionnaire (M2C) (46)

These questionnaires assessed symptoms related to TBI, PTSD, sleep disturbances, quality of life, depression, anxiety, socioeconomic variables, and previous psychedelic experiences. They were administered at two timepoints: 4 weeks prior to the retreat (baseline) and 4 weeks after leaving the retreat (follow-up).

#### Scoring and standardisation of psychological measures

For several psychological instruments, we reported both raw scores and standardised scores (e.g., T-scores for PROMIS-SD scale, conversion for the W-EMWS) to improve interpretability and cross-study comparability. Raw scores reflect the direct sum of item responses, while standardised scores are computed through validated conversion algorithms that align individual results with reference population distributions (47, 48). This dual reporting approach follows best practices in psychometric assessment and ensures that observed changes can be interpreted both in absolute terms and in relation to normative benchmarks. Standardised scores were derived using the official scoring tools or conversion tables associated with each instrument.

### EEG data

Resting state EEG data were collected at the retreat site in Jamaica pre- and post-psilocybin use to assess changes in brain function. All resting-state measurements were conducted with participants seated facing a blank wall, instructed to keep their eyes open throughout the session. EEG recordings were taken using a dry cap with 19 electrodes and 2 reference electrodes, following the 10/20 international system during resting state. The first EEG recording occurred on day one of the retreat before any psilocybin administration, and the second on the final day of the retreat, over 36 hours from the final ceremony. This data aimed to provide insights into changes in brain connectivity and functional dynamics following psilocybin therapy.

### Psilocybin dosage and administration

During the retreat, participants consumed psilocybin in the form of dried Psilocybe cubensis mushrooms prepared as a tea across two ceremonial sessions spaced 48 hours apart. The initial dosage of psilocybin was between 1.5g and 3.5g of dried psilocybin mushrooms, with an option for an increased dose, between 3g and 5g, for the second ceremony. Participants were consulted individually with retreat staff to decide on the individual dosing and consumed the psilocybin under the supervision of retreat staff.

We acknowledge that naturally occurring psilocybin concentrations vary significantly, typically ranging between 0.5%–2% of dried mushroom weight (49). Drawing from the latter study, we estimated that the administered doses of 1.5g–3.5g during the first session corresponded to approximately 15–35 mg of psilocybin. The tea preparation method was chosen to reduce gastrointestinal discomfort and enhance participant acceptability, though whether this may slightly alter absorption kinetics compared to ingestion of dried material is still speculative.

The 48-hour interval between sessions was implemented based on a balance between practical retreat logistics and preclinical evidence that tolerance to psilocybin develops rapidly after repeated exposure within a short time span (50). These preliminary findings informed our decision to moderately increase the dose in the second ceremony (typically 3g–5g) to maintain experiential potency and remain within the boundaries of a supra-therapeutic dose (49). Additional booster doses (up to 1g) were offered at the discretion of retreat staff and participants within one hour of initial ingestion to titrate intensity within the expected onset window (20–40 min) (51).

Importantly, we acknowledge that the use of natural mushroom preparations introduces pharmacokinetic uncertainty, both in dose quantification and in the profile of accompanying alkaloids. A recent systematic review by Meshkat et al. (52) emphasises that psilocybin pharmacokinetics show significant inter-study variability due to differences in formulation, route of administration, species, and enzymatic metabolism—especially involving CYP enzymes that mediate drug–drug interactions (52). While most pharmacokinetic studies to date have involved synthetic psilocybin, only one published *in vivo* study has assessed *psilocin* pharmacokinetics from natural products: Chen et al. (53) reported rapid absorption and measurable plasma concentrations in rats following oral administration of Gymnopilus spectabilis extract, highlighting the feasibility (and ultimately, the need) of pharmacokinetic evaluation from mushroom preparations (53).

Moreover, emerging preclinical data suggests that psilocybin-containing mushroom extracts may have distinct and potentially more robust neurobiological effects compared to chemically synthesised psilocybin alone. Shahar et al. (54) demonstrated that psychedelic mushroom extract (PME) elicited greater and more sustained upregulation of synaptic plasticity markers and induced unique frontal cortex metabolomic profiles compared to synthetic psilocybin in mice (54). These findings support the hypothesis that additional bioactive compounds within the mushroom matrix may enhance or prolong psilocybin’s therapeutic effects.

It is also important to emphasise that the current state of research on synthetic psilocybin and related pharmacokinetic variability would benefit from a more comprehensive characterisation. As reviewed by Otto et al. (55), substantial heterogeneity exists in synthetic psilocybin formulations used in clinical trials, with many studies omitting key details such as drug purity, dose correction, and formulation specifications (55). Inconsistent reporting of whether dose calculations reflect free base or salt forms has further impeded cross-study comparisons and dose-normalised pharmacokinetic modelling. Therefore, while synthesised psilocybin may offer standardisation in principle, in practice, formulation ambiguity continues to challenge data interpretation.

### Data analysis

Data analysis was designed to explore the psychological, behavioural, cognitive, and neurofunctional effects of psilocybin therapy. Analyses for psychological and behavioural outcomes were conducted using the statistical software R (56). All statistical tests were two-tailed, with significance set at p < 0.05. Descriptive statistics were computed for all variables, including means, standard deviations, and ranges to summarise the demographic and baseline characteristics of the study participants. For comparing pre- and post-treatment scores, paired t-tests, also adjusted for multiple testing by means of Bonferroni correction, were employed to evaluate the significance of changes across various psychological and cognitive measures.

#### Subgroup analyses controlling for prior psychedelic use

We conducted additional pre-post treatment analyses stratified by prior psychedelic use. Participants were categorised as psychedelic-naïve (no prior use) or psychedelic-experienced (any prior use). Paired t-tests were performed within each subgroup to evaluate whether therapeutic outcomes varied based on previous psychedelic exposure. Full details are provided in **Supplementary Material S1**.

### EEG data analysis

EEG data were processed to evaluate within-subject effects pre- and post-psilocybin use. The analysis focused on changes in functional connectivity associated with the TBIs, aiming to identify alterations in brain network dynamics associated with the therapeutic effects of psilocybin. Statistical comparisons were performed on band-specific EEG measures across canonical frequency bands (delta, theta, alpha, beta). Paired, non-parametric Wilcoxon signed-rank tests were used to assess differences in both spectral power and variance across electrodes within each frequency band. To evaluate inter-band distinctions, pairwise comparisons were conducted between delta and each of the higher frequency bands, as well as between theta and alpha, within each subject. P-values were transformed using the negative base-10 logarithm (–log_10_(p)) for interpretability, with values exceeding 1.3 indicating statistical significance at p < 0.05. False discovery rate (FDR) correction was applied to control for multiple comparisons across electrodes. This approach enabled spatially resolved identification of significant changes in oscillatory dynamics and enhanced network differentiation following psilocybin administration. Full descriptions of the EEG analysis are provided in the **Supplementary Material S2**.

## Results

### Descriptive statistics of study participants

**Table 1** summarises the demographic characteristics of the study participants (n=21).

**Table 1:** Demographic characteristics of the study participants.

Variable	EEG (n=21)	Psychol (n=13)
Age		
Mean (SD)	38.524 (6.202)	38.154 (6.606)
Range	26.000 - 50.000	26.000 - 48.000
Gender		
Male	21 (100.0%)	13 (100.0%)
Nationality		
CA - Canada	2 (9.5%)	1 (7.7%)
GB - United Kingdom	1 (4.8%)	1 (7.7%)
US - United States	18 (85.7%)	11 (84.6%)
Highest Education		
Bachelor’s degree	7 (33.3%)	5 (38.5%)
Doctorate or professional degree (e.g., MD, PhD, Law Degree, JD)	1 (4.8%)	1 (7.7%)
Graduated high school	1 (4.8%)	1 (7.7%)
Less than high school	1 (4.8%)	0 (0.0%)
Master’s degree	4 (19.0%)	3 (23.1%)
Some college, no degree	5 (23.8%)	2 (15.4%)
Trade/Technical school	2 (9.5%)	1 (7.7%)
Employment		
Employed full time (40 or more hours per week)	11 (52.4%)	6 (46.2%)
Homemaker	2 (9.5%)	2 (15.4%)
Retired	2 (9.5%)	2 (15.4%)
Self-employed	4 (19.0%)	2 (15.4%)
Unable to work	2 (9.5%)	1 (7.7%)
Marital Status		
Divorced	2 (9.5%)	1 (7.7%)
Married	13 (61.9%)	9 (69.2%)
Single (never married)	3 (14.3%)	3 (23.1%)
Separated	1 (4.8%)	0 (0.0%)
In a relationship	2 (9.5%)	0 (0.0%)
Monthly Household Income (USD)		
Mean (SD)	33880.952 (50500.967)	34692.308 (55248.506)
Range	0.000 - 200000.000	3000.000 - 200000.000

EEG data were collected from all 21 participants and psychological data from a subset of 13 participants. The mean age of participants was relatively consistent across groups, with a mean of 38.52 years (SD = 6.20) for the EEG group and 38.15 years (SD = 6.61) for the psychological group. All participants were male. Most participants were from the United States, with slight variations among groups: 85.7% (n=18) in the EEG group and 84.6% (n=11) in the psychological group. Educational backgrounds showed that a Bachelor’s degree was the most common highest level of education, with 33.3% (n=7) in the EEG group and 38.5% (n=5) in the psychological group. Employment status was similar, with around half of the participants employed full-time in each group: 52.4% (n=11) for EEG and 46.2% (n=6) for the psychological group. The majority were married, with slight variations: 61.9% (n=13) in the EEG group and 69.2% (n=9) in the psychological group. Mean monthly household income was (United States Dollars [USD]) $34,692 (SD = $55,249) in the psychological group and $33,881 (SD = $50,501) in the EEG group.

### Psychiatric history and comorbidities

**Table 2** details the psychiatric history and comorbidities of the participants. Previous usage of psychiatric medications varied slightly, with the psychological group having the highest percentage of participants using medications 21–50 times (23.1%, n=3) compared to the EEG group (19.0%, n=4). Major depressive disorder was most prevalent in the psychological group at 38.5% (n=5), compared to 23.8% (n=5) in the EEG group. Anxiety disorders were reported by 76.9% (n=10) in the psychological group, higher than in the EEG group (57.1%, n=12). PTSD was similarly prevalent across both groups: 85.7% (n=18) in the EEG group and 84.6% (n=11) in the psychological group. Substance use disorder and alcohol dependence showed slight variations in percentage with 2 in each group, (9.5%) in the EEG and (15.4%) psychological groups. Chronic pain was reported by 76.9% (n=10) in the psychological group, compared to 71.4% (n=15) in the EEG group. ADHD was reported to be slightly higher in the psychological group (30.8%, n=4) and the EEG group (23.8%, n=5).

**Table 2:** Psychiatric history and comorbidities of the study participants.

Variable	EEG (n=21)	Psychol (n=13)
Prev Psychedelic Usage		
2–5 times	6 (28.6%)	4 (30.8%)
21–50 times	4 (19.0%)	3 (23.1%)
6–10 times	2 (9.5%)	0 (0.0%)
More than 100 times	1 (4.8%)	1 (7.7%)
Never	7 (33.3%)	4 (30.8%)
Only once	1 (4.8%)	1 (7.7%)
Major Depressive Disorder		
Major depressive disorder	5 (23.8%)	5 (38.5%)
No	16 (76.2%)	8 (61.5%)
Anxiety Disorder		
Anxiety disorder (e.g., OCD)	12 (57.1%)	10 (76.9%)
No	9 (42.9%)	3 (23.1%)
Post-traumatic Stress Disorder (PTSD)		
No	3 (14.3%)	2 (15.4%)
PTSD	18 (85.7%)	11 (84.6%)
Substance Use Disorder		
No	19 (90.5%)	11 (84.6%)
Substance use disorder	2 (9.5%)	2 (15.4%)
Alcohol Dependence		
Alcohol dependence	1 (4.8%)	1 (7.7%)
No	20 (95.2%)	12 (92.3%)
Eating Disorder		
Eating disorder	1 (4.8%)	1 (7.7%)
No	20 (95.2%)	12 (92.3%)
**ADHD**		
ADHD	5 (23.8%)	4 (30.8%)
No	16 (76.2%)	9 (69.2%)
**Phobia**		
No	20 (95.2%)	12 (92.3%)
Phobia (e.g. social phobia)	1 (4.8%)	1 (7.7%)
**Chronic Pain**		
No	6 (28.6%)	3 (23.1%)
Yes	15 (71.4%)	10 (76.9%)

### Pre and post-retreat comparisons

**Table 3** presents the comparison of study variables pre- and post- attendance to the psilocybin retreat.

**Table 3:** Comparison of pre- and post-treatment outcomes in study variables following psilocybin therapy (paired T-test) *Bonferroni correction.

Psychological Outcome	Pre-Treatment (n=13)	Post-Treatment (n=13)	p-value	p-adjusted*
PHQ-9			< 0.001	< 0.001
Mean (SD)	15.538 (4.115)	5.308 (5.186)		
Range	10.000 - 25.000	0.000 - 15.000		
Qolibri-OS			< 0.001	0.007
Mean (SD)	41.614 (13.764)	67.522 (17.277)		
Range	18.750 - 61.810	43.750 - 97.220		
RPQ (Rivermead)			< 0.001	0.019
Mean (SD)	34.692 (9.013)	18.538 (12.258)		
Range	19.000 - 47.000	0.000 - 33.000		
PCL-5			0.010	0.235
Mean (SD)	40.769 (16.799)	20.923 (19.410)		
Range	9.000 - 65.000	0.000 - 61.000		
PROMIS-SD Raw			0.002	0.045
Mean (SD)	32.538 (5.301)	23.077 (8.271)		
Range	22.000 - 40.000	10.000 - 40.000		
PROMIS-SD T-Score			0.004	0.102
Mean (SD)	63.938 (6.596)	53.231 (10.383)		
Range	52.200 - 76.500	35.900 - 76.500		
W-EMWS Raw			0.003	0.066
Mean (SD)	20.692 (4.733)	27.000 (4.950)		
Range	11.000 - 29.000	16.000 - 35.000		
W-EMWS Conversion			0.004	0.083
Mean (SD)	19.396 (3.277)	24.903 (5.212)		
Range	13.330 - 26.020	16.360 - 35.000		
STAI			< 0.001	0.013
N-Miss	0	1		
Mean (SD)	53.769 (7.259)	38.500 (11.533)		
Range	36.000 - 62.000	21.000 - 58.000		
M2C			< 0.001	0.016
N-Miss	0	1		
Mean (SD)	2.174 (0.677)	0.959 (0.871)		
Range	0.940 - 3.440	0.000 - 2.310		

### Behavioural questionnaires

**Figure 1:**
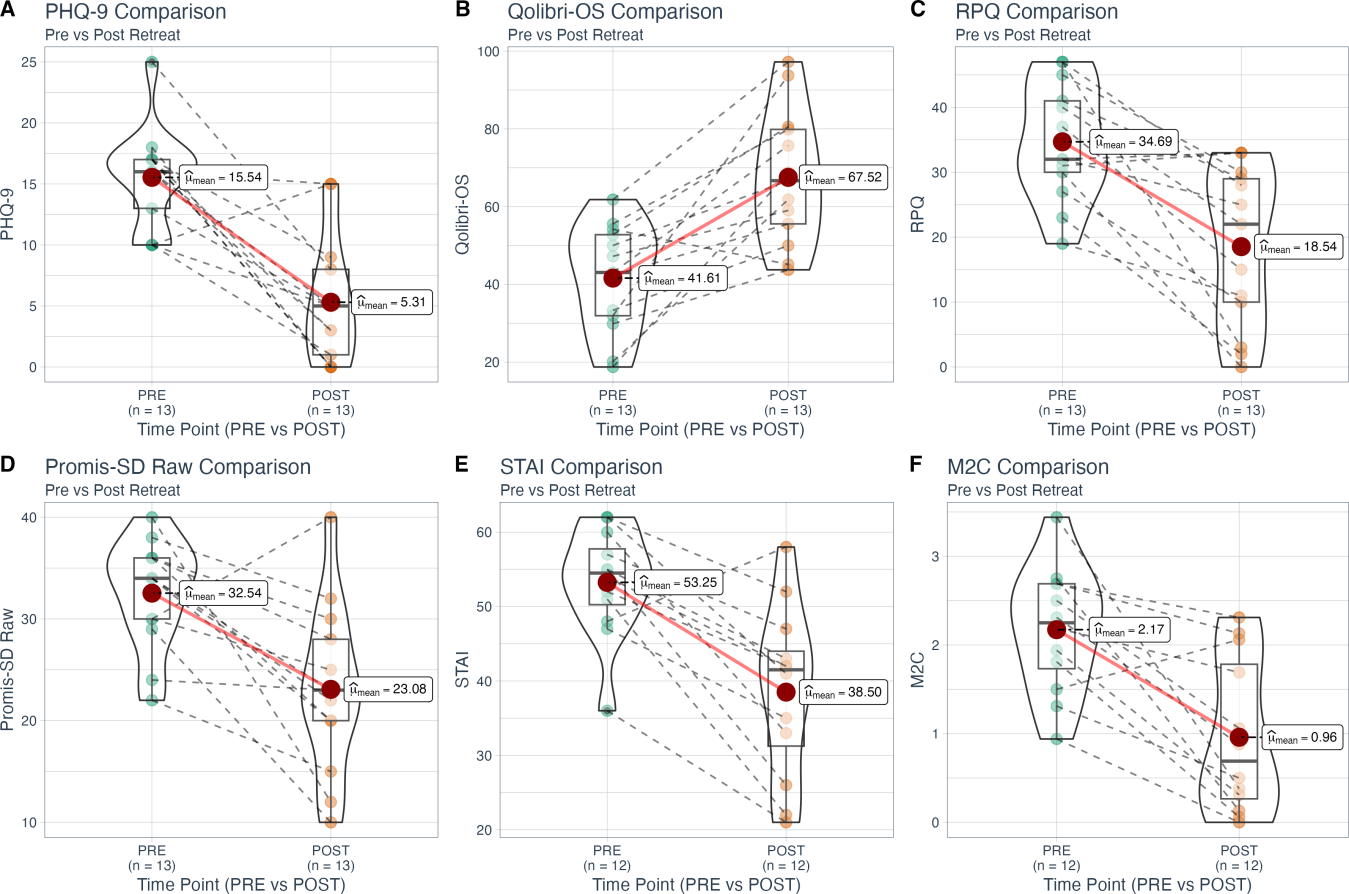
Within-subject comparisons of key variables pre- and post-psilocybin retreat. Violin plots display the distribution of scores at baseline (PRE, left) and post-retreat (POST, right) for: **(A)** PHQ-9, **(B)** Qolibri-OS, **(C)** RPQ, **(D)** Promis-SD Raw, **(E)** STAI, and **(F)** M2C. Boxplots embedded within the violin plots indicate the median and interquartile ranges, with individual participant data points connected by dashed lines to illustrate within-subject changes.

Behavioural questionnaires were collected pre- and post-retreat from 13 participants and compared between pre and post treatment scenarios (**Figure 1**). The PTSD Checklist (PCL-5) scores significantly decreased from a baseline mean of 40.769 (SD = 16.799) to a follow-up mean of 20.923 (SD = 19.410, p = 0.010), but this result did not survive the Bonferroni correction (adjusted p = 0.235).

The Patient Health Questionnaire (PHQ-9) scores significantly decreased from a baseline mean of 15.538 (SD = 4.115), indicating moderate to severe depression, to a follow-up mean of 5.308 (SD = 5.186, p < 0.001), and this result survived the Bonferroni correction (adjusted p < 0.001).

The State-Trait Anxiety Inventory (STAI) scores significantly decreased from a baseline mean of 53.769 (SD = 7.259) to a follow-up mean of 38.500 (SD = 11.533, p < 0.001), which also survived the Bonferroni correction (adjusted p = 0.013).

The (RPQ) scores significantly decreased from a baseline mean of 34.692 (SD = 9.013) to a follow-up mean of 18.538 (SD = 12.258, p < 0.001), surviving the correction (adjusted p = 0.019).

Quality of Life After Brain Injury (Qolibri-OS) scores increased significantly from a baseline mean of 41.614 (SD = 13.764) to a follow-up mean of 67.522 (SD = 17.277, p < 0.001), and this survived the correction (adjusted p = 0.007).

The PROMIS Sleep Disturbance Short Form (PROMIS-SD) raw scores increased significantly from a baseline mean of 32.538 (SD = 5.301) to a follow-up mean of 23.077 (SD = 8.271, p = 0.002), and the T-scores also showed a significant decrease from a baseline mean of 63.938 (SD = 6.596) to a follow-up mean of 53.231 (SD = 10.383, p = 0.004). However, neither of these results survived Bonferroni correction (adjusted p = 0.045 and 0.102, respectively).

The Warwick-Edinburgh Mental Well-being Scale (W-EMWS) raw scores increased significantly from a baseline mean of 20.692 (SD = 4.733) to a follow-up mean of 27.000 (SD = 4.950) (p = 0.003), but this result did not survive Bonferroni correction (adjusted p = 0.066). Similarly, the conversion scores also improved significantly from a baseline mean of 19.396 (SD = 3.277) to a follow-up mean of 24.903 (SD = 5.212) (p = 0.004), but this did not survive Bonferroni correction (adjusted p = 0.083).

The Military to Civilian Questionnaire (M2C) scores decreased significantly from a baseline mean of 2.174 (SD = 0.677), indicating difficulties in reintegration, to a follow-up mean of 0.959 (SD = 0.871, p < 0.001), and this result survived the Bonferroni correction (adjusted p = 0.016).

Subgroup-specific pre-post treatment analyses stratified by prior psychedelic use are provided in **Supplementary Material S1**. Briefly, among participants with a history of prior psychedelic use (n = 9), statistically significant reductions were observed across all primary psychological outcome measures (PHQ-9, STAI, RPQ, M2C, PROMIS-SD) and improvements in quality of life (Qolibri-OS). In contrast, psychedelic-naïve participants (n = 4) exhibited numerically similar trends toward improvement across the same outcomes; however, none of these changes reached statistical significance, and associated effect sizes were small to moderate with wide confidence intervals. These results suggest that, while the directionality of therapeutic effects was comparable between groups, the magnitude and robustness of change were greater among individuals with prior psychedelic exposure, potentially reflecting sample size differences and greater within-group variance in the naïve subgroup.

### Frequency bands correlations

**Figure 2:**
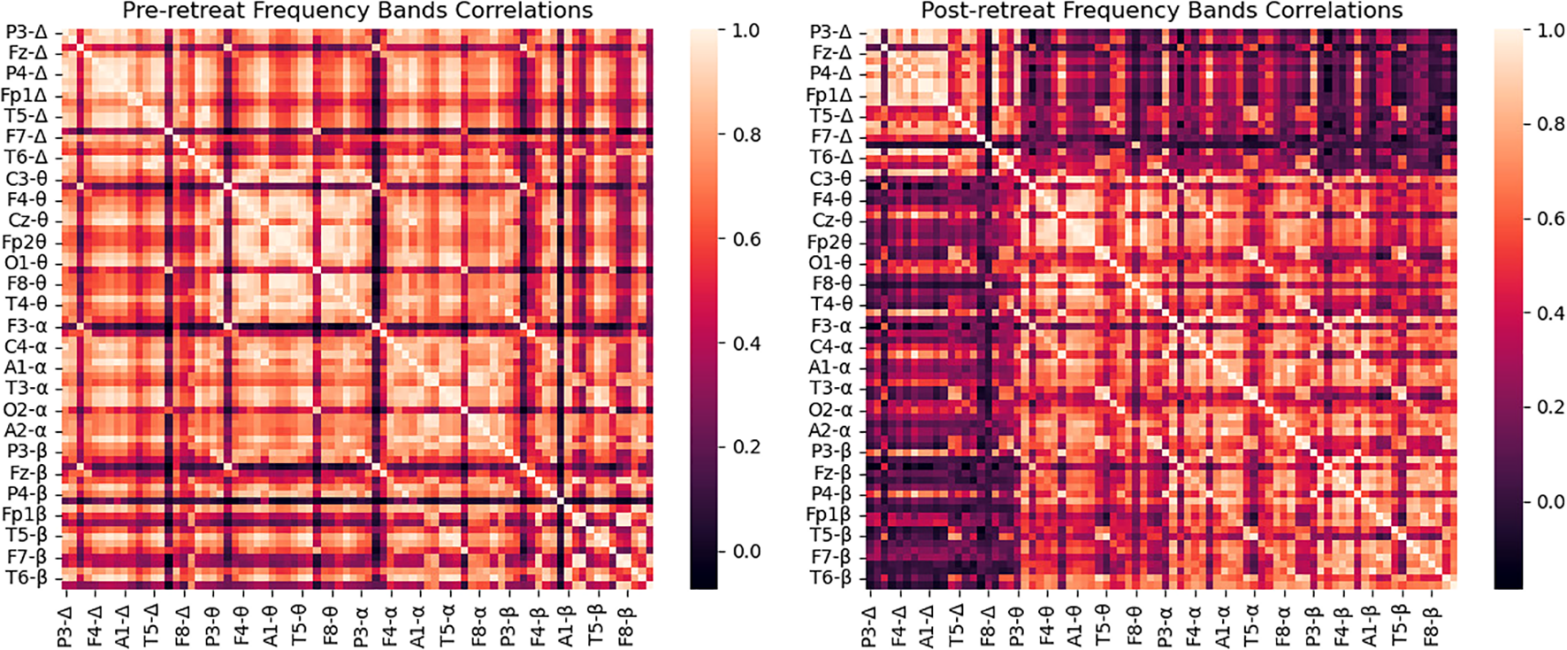
Correlation matrix of delta band powers for pre-retreat and post-retreat recordings, showing significant changes post-treatment.

Prior to any psilocybin use, strong correlations were observed between the delta band and the alpha band. Post-retreat these correlations weakened. Compared to pre-retreat recordings, we observed new connections between theta and alpha waves. Compared to pre-retreat recordings, we observed beta waves, reduced correlations with delta waves (shown in **Figure 2**).

### Frequency bands headplots

**Figure 3:**
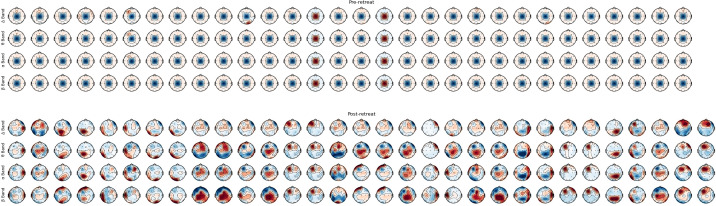
Montage of bands’ power, from pre-retreat and post-retreat recordings.

Prior to psilocybin use at the retreat, EEG recordings revealed widespread overactivity across all frequency bands, particularly in the frontal, temporal, and parietal lobes. Post-retreat, reductions in delta power, particularly in frontal and temporal regions, were identified. We observed normalisation of abnormal theta activity in temporal lobes, modulation of alpha power, specifically decreased activity in frontal and central regions, and increased beta power in frontal and central regions; post-retreat (shown in **Figure 3**).

### Frequency bands power distribution

**Figure 4:**
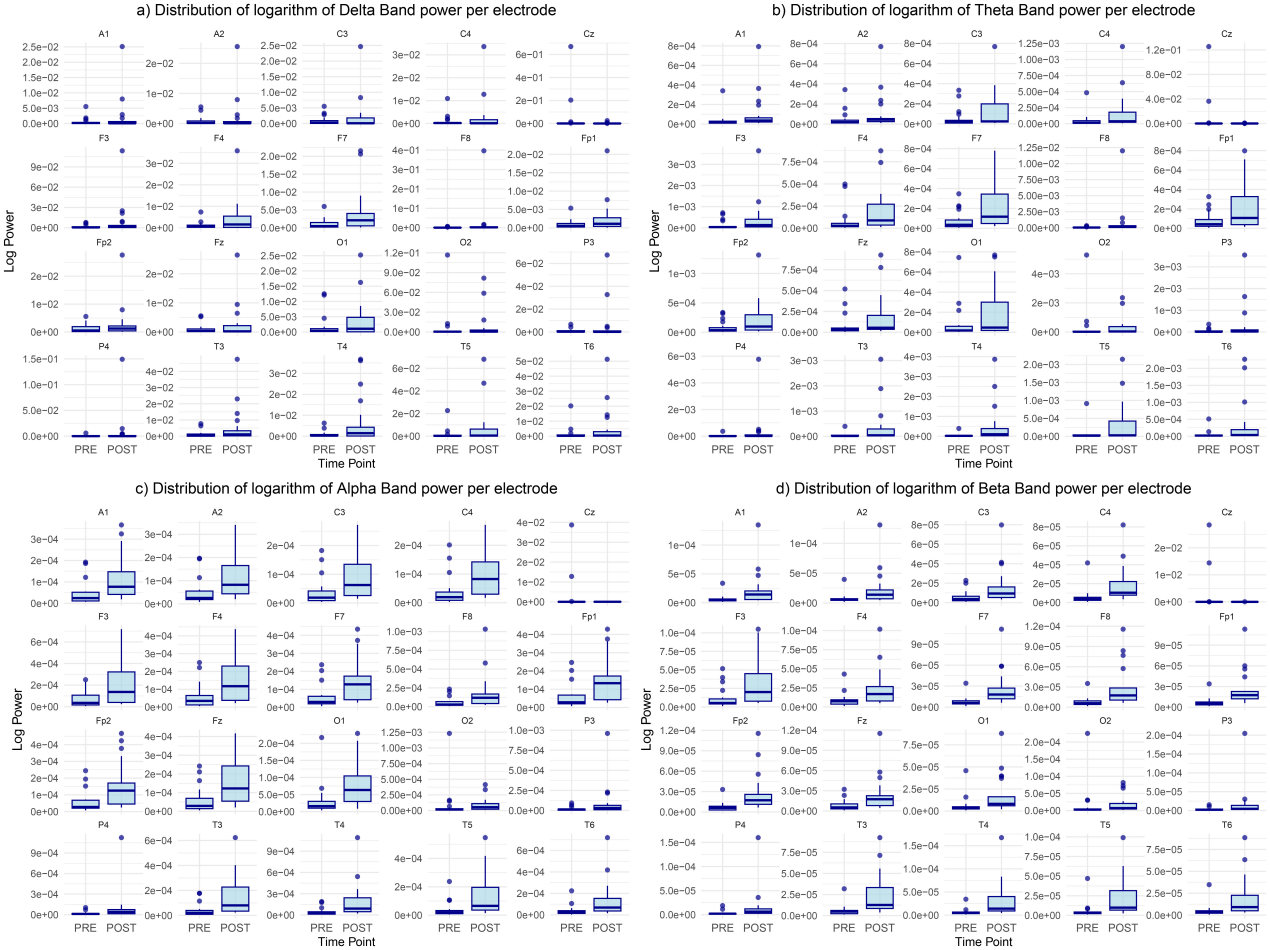
Distribution of the logarithm of bands’ power for **(a)** delta, **(b)** theta, **(c)** alpha, and **(d)** beta bands, showing differences between pre-retreat and post-retreat recordings.

Analysing decibel power across frequency bands pre and post-retreat revealed significant reduction in the delta band power from the pre-retreat to the post-retreat across brain regions with the exception of the Cz electrode in which we observe an increase in the delta band power from the pre-retreat to the post-retreat. This is shown in **Figure 4a**. We also see that this reduction in power is consistent in the frontal, fronto-polar and temporal regions.

Same reduction in power is noticed in the theta band with the Cz electrode theta power still increasing from the pre to post-retreat recordings. This is shown in **Figure 4b**. A much more widespread post-retreat theta band power is also observed as opposed to the delta band. The post-retreat theta band power of the parietal regions has similar variance with its pre-retreat counterpart with slight reduction in the mean suggesting less changes in the parietal theta band activity.

Similar changes are noticed in the alpha band as shown in **Figure 4c** with some exceptions. There exist similar but more widespread alpha activity levels in the frontopolar and frontal regions of the post-retreat recording. There is also a slight increase in occipital alpha power activity from the pre-retreat recordings to the post-retreat recordings.

**Figure 4d** shows consistent reduction in the beta band power from pre to post-retreat recordings with the exception of the Cz and 02 electrodes. The post-retreat beta band power is much more widespread in all brain regions.

The consistent reduction of activity in the delta and theta band after psychedelic treatment, fairly consistent or improved alpha band activity in the post-retreat recordings and increase in the variance of the post-retreat band power from the delta to the alpha could indicate improved alpha functioning which is predominant in simulated sleep as opposed to delta waves whose abnormalities are consistent with some level of psychotic activity.

These qualitative shifts were corroborated by quantitative analyses (see **Supplementary Material S2**). Wilcoxon signed-rank tests comparing pre- and post-retreat variance across the frequency bands revealed a significant increase in spatial variance for theta (p = 4.17 × 10^−6^), alpha (p = 1.00 × 10^−6^), and beta bands (p = 1.00 × 10^−6^), while delta variance showed a modest increase (p ≈ 0.039). These findings confirm that spatial differentiation of band power increased following treatment, particularly in higher-frequency oscillations. Log-scaled violin plots further demonstrated this increased variability, with the beta band showing the most pronounced post-treatment spread in variance across electrodes.

### Coherence: connectivity matrix and connectivity circle

**Figure 5:**
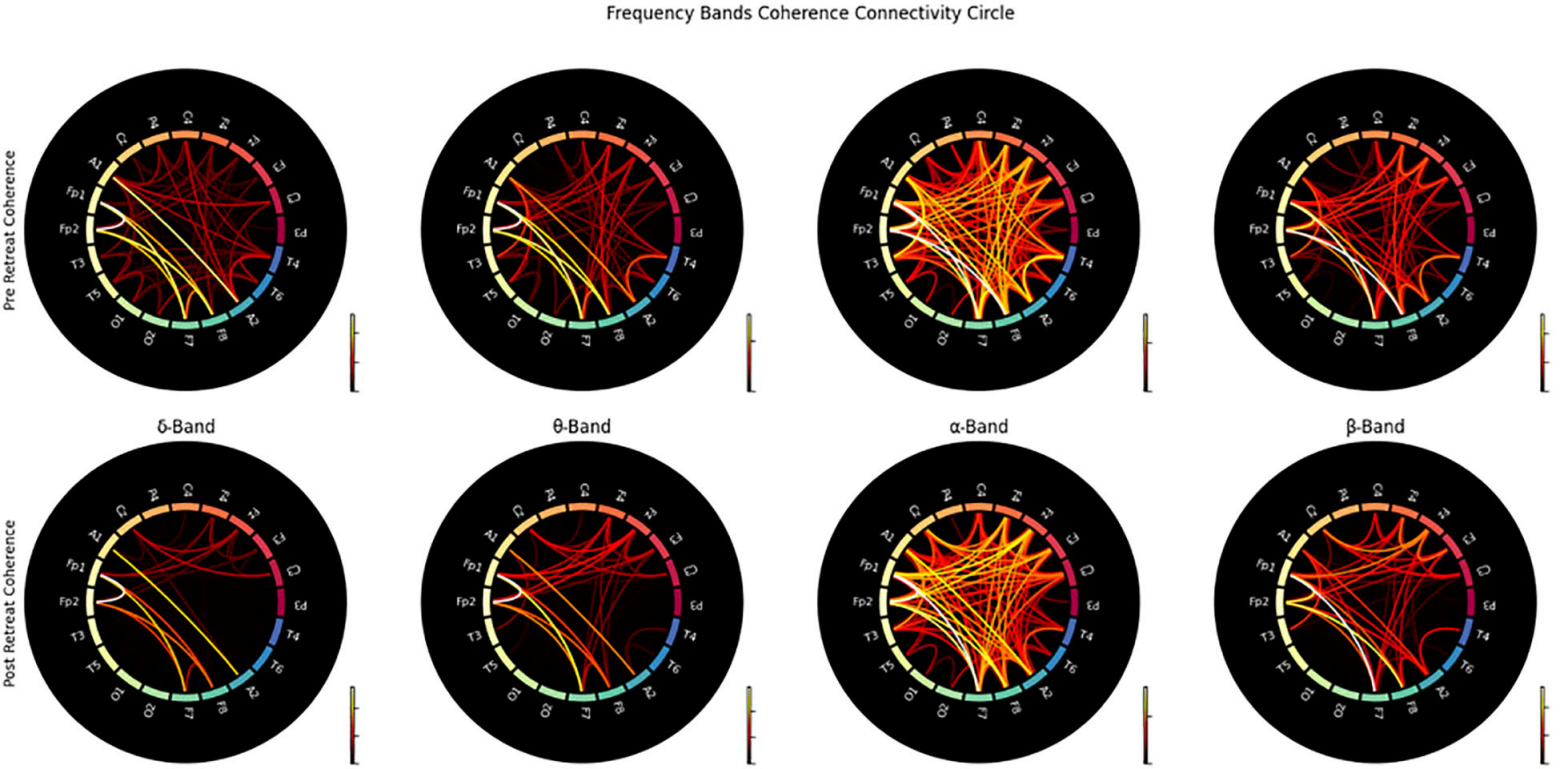
Connectivity circles illustrating the coherence between electrodes for pre-retreat and post-retreat recordings across delta, theta, alpha, and beta bands.

The connectivity matrices analysed pre- and post-retreat reveal notable changes in patterns of brain connectivity. Prior to psilocybin use, there is widespread, moderate connectivity without well-defined clusters. Post-retreat, increased connectivity strength was observed, especially in the frontal and central regions for delta, theta, and alpha bands. The delta band exhibits the most significant increase. Beta band connectivity remained widespread, with some strengthening in frontal and central regions post-treatment. A stronger connection in frontal and central regions was observed (shown in **Figure 5**).

### Short Time Fourier Transform: representational similarity analysis

**Figure 6:**
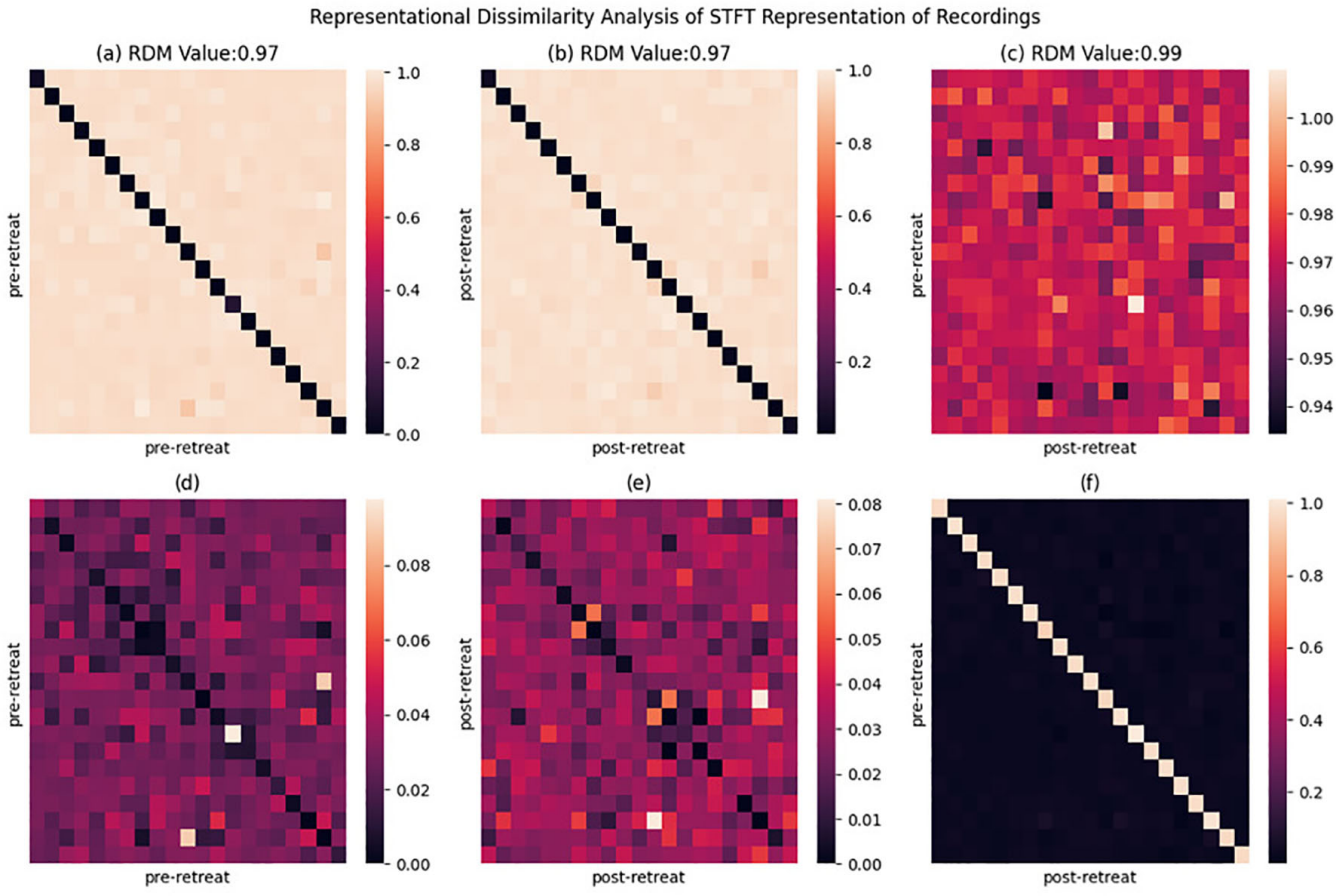
Representational dissimilarity matrix (RDM) heatmaps displaying the cosine dissimilarity between pre-retreat and post-retreat time-frequency spectrograms, as well as within pre-retreat and within post-retreat groups. Each pixel indicates the dissimilarity between two recordings. **(a)** Pre-retreat vs pre-retreat RDM showing within-condition dissimilarity patterns. **(b)** Post-retreat vs post-retreat RDM showing within-condition dissimilarity patterns. **(c)** Pre-retreat vs post-retreat RDM showing between-condition dissimilarity patterns. **(d)** Deviation between perfect RDM and pre-retreat vs pre-retreat RDM matric, indicating deviation from ideal dissimilarity measure. **(e)** Deviation between perfect RDM and post-retreat vs post-retereat RDM matrix. **(f)** Deviation between perfect RDM and pre-retreat vs post-retreat RMD matrix. RDM values in title indicate proximity to perfect dissimilarity measure.

Pre psilocybin use Representational Dissimilarity Matrix (RDM) illustrates widespread bright areas, indicating high dissimilarity between pre-retreat EEG recordings among the participants suggesting a notable variability in the initial brain representations as shown in **Figure 6a**. The same is noticed in the post-retreat RDM in **Figure 6b**. **Figure 6c** shows an increase in dissimilarity values in the diagonal (pre and post recording from the same subject), while showing less dissimilarity in between subjects, yet, the pre-post-retreat RDM as shown in **Figure 6c** have a higher RDM score of 0.99 compared to the pre-pre and post-post RDM in **Figures 6a, b**. **Figures 6d–f** show how distant **Figures 6a–c** are from an ideal RDM matrix. This indicates the deviation of subjects from a common state in the pre-retreat phase to another in the post-retreat phase.

### Canonical correlation analysis: frequency bands power

**Figure 7:**
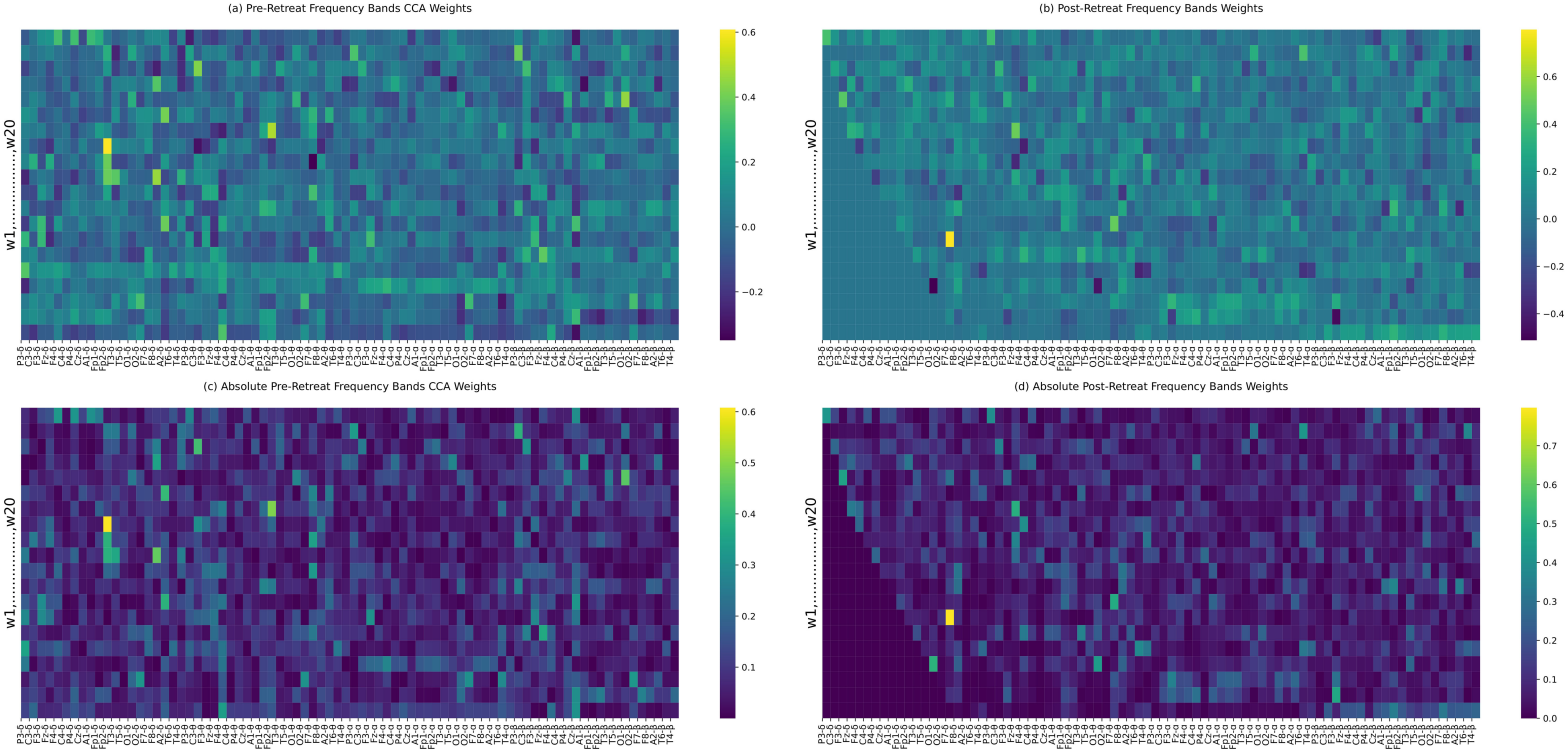
**(a–d)** Canonical correlation analysis (CCA) weights between pre-retreat **(a)** and post-retreat **(b)** frequency bands’ power. Panels **(c)** and **(d)** display the absolute values of the weights from **(a)** and **(b)**, respectively. Significant changes in canonical weights, particularly in the delta band, are highlighted.

Canonical correlation analyis (CCA) of EEG bands power identifies shared patterns (correlation, covariance) or relationships between the pre and post-retreat recording band powers. While there are reduced correlations in the pre-post-retreat delta band power as shown in **Figures 7a–d** indicating the altering of the delta band functioning after the retreat, correlation patterns from the pre to the post-retreat tend towards becoming sparse in the alpha and beta band. This suggests changes in the communication pattern and space of the brain networks after the retreat.

In addition to global correlation changes, electrode-wise statistical comparisons between delta and higher frequency bands (theta, alpha, beta) revealed a post-retreat increase in inter-band distinction at multiple sites, particularly in P3, C3, C4, P4, Cz, T5, and A2 (see **Supplementary Material S2**). These regional effects highlight an increased ability of the brain to differentiate between frequency-specific information channels, suggesting reduced broadband interference and improved oscillatory specificity post-intervention. No significant divergence was found between theta and alpha bands, despite the observed reduction in correlation, indicating preservation of their spatial similarity.

### Canonical correlation analysis: EEG multi-channel time series

**Figure 8:**
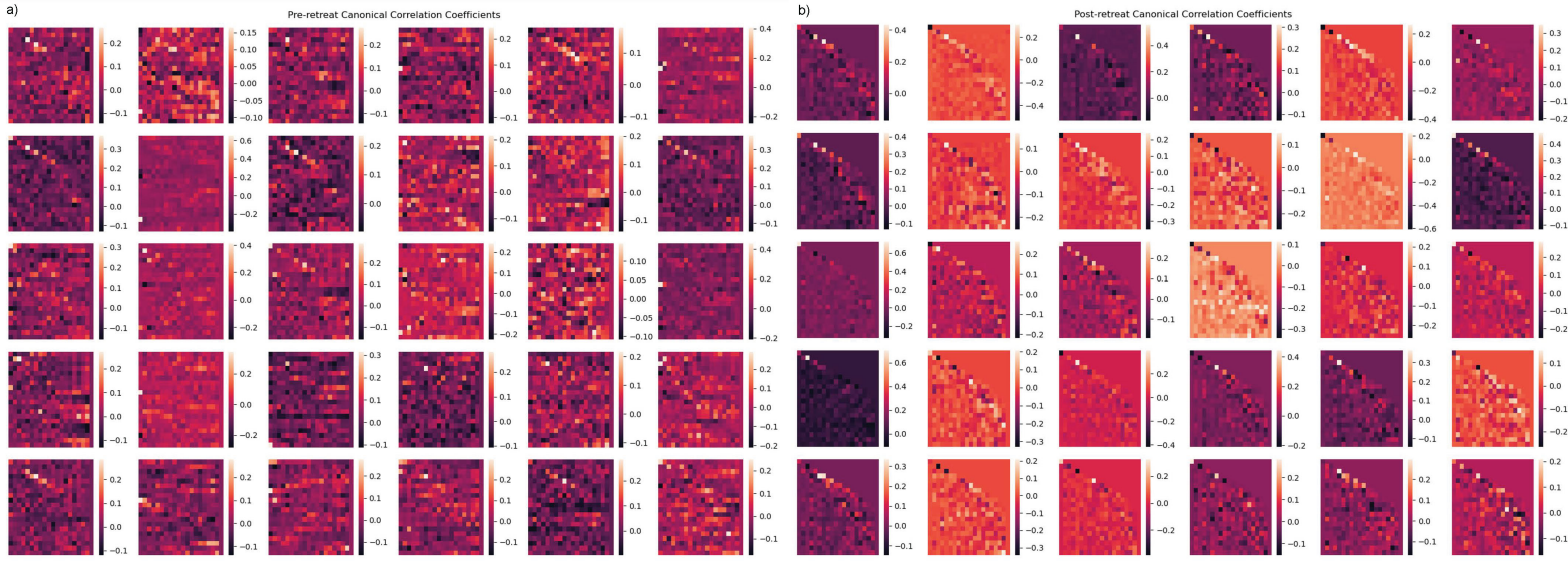
**(a, b)** Canonical correlation analysis (CCA) results for multivariate time-series analysis. **(a)** Independent variable canonical weights (pre-retreat). **(b)** Dependent variable canonical weights (post-retreat), showing repeated structure in post-retreat weights.

The post-retreat analysis of overall network connectivity indicates substantial changes compared to the pre-retreat condition. The post-retreat Y-weight heatmap reveals an augmented strength of positive correlations, marked by increased red and orange hues. A reduction in blue and purple areas in the post-retreat data implies a decrease in negative correlations. High connectivity clusters, particularly central hubs, persist post-retreat. These clusters appear more well-defined and spatially distinct in the post-retreat Y-weight heatmap. Subtle asymmetries observed in the pre-retreat data become less apparent post-retreat- shown in **Figures 8a, b**.

## Discussion

Our findings from this observational study of veterans experiencing psychological distress and with a history of TBI provide further evidence for the multidimensional therapeutic benefits of psilocybin retreats. After applying Bonferroni correction, the results indicate statistically significant improvements in depressive symptoms (PHQ-9), anxiety (STAI), quality of life (Qolibri-OS), and reintegration difficulties (M2C) scores four weeks post-retreat. These findings highlight the potential positive outcomes from attendance at the retreat. However, while improvements were observed for PTSD symptoms (PCL-5), sleep disturbances (PROMIS-SD), and mental well-being (W-EMWS), these results did not survive the Bonferroni correction and should be interpreted with caution. Noteworthy, the significant improvement in Rivermead Post-Concussion Questionnaire scores can support the potential of psilocybin retreat programs in alleviating subjective symptoms of TBI. Overall, the results suggest promising therapeutic benefits, though further studies with larger sample sizes and more rigorous controls are necessary to confirm these findings.

Subgroup analyses controlling for prior psychedelic experience further clarified these results (**Supplementary Material S1**). Significant improvements across multiple behavioural and wellbeing outcomes were predominantly observed in psychedelic-experienced participants, while psychedelic-naïve individuals demonstrated trends toward improvement without reaching statistical significance. Given the small number of psychedelic-naïve participants (n = 4), these differences should be interpreted cautiously. Nonetheless, the consistency of directional effects across groups might suggest that prior psychedelic exposure may enhance—but is not necessary for—the therapeutic response to psilocybin in structured retreat settings.

These improvements in mental health and wellbeing have been shown previously by use of psilocybin in both retreat settings (57), naturalistic environments (58), and clinical settings for major depressive disorder (MDD) (59) and anxiety (60). Furthermore, evidence of psychedelic retreat programs in general have shown similar findings for improved mental health and wellbeing in military veterans (61) and the wider populations (62).

In line with our hypothesis and prior literature on EEG abnormalities in TBI (18, 19), neurofunctional evaluation post-retreat revealed systematic modulation of frequency band power and connectivity, suggesting enhanced neural integration and cognitive-emotional regulation.

Consistent with this, statistical testing showed that variance in band power increased significantly post-retreat for theta, alpha, and beta bands, with spatially resolved differences detected across key centro-parietal and temporal electrodes. These findings indicate enhanced functional differentiation of oscillatory activity, particularly in frequency bands associated with attention and emotional regulation.

Pre- to post-retreat comparisons of frequency band correlations indicated a reduction in delta dominance and a reconfiguration of cross-frequency interactions. In particular, the weakening of delta-beta correlations—often elevated in TBI and associated with disrupted cortical control—suggests improved filtering of irrelevant stimuli and enhanced vigilance. Simultaneously, the emergence of theta–alpha connectivity patterns may reflect more effective cognitive and emotional processing, consistent with prior work linking these bands to memory integration and affective regulation (35, 36).

Frequency bands head plots revealed pre- to post-retreat, reductions in delta power, particularly in frontal and temporal regions—areas classically affected in TBI (38)— signal, potentially pointing towards improvements in emotional regulation and processing control (34).

The normalisation of abnormal theta activity—which increase is among the most consistently reported EEG abnormalities in TBI patients (19)— in temporal lobes post-retreat, may suggest enhanced emotional processing.

Modulation of alpha power specifically decreased activity in frontal and central regions, which can indicate heightened attention and improved cognitive processing directed towards emotional information.

Beta band power increases, especially in frontal and parietal regions, point to greater cognitive engagement and sensory integration, aligning with prior intracranial EEG findings linking beta oscillations to recovery trajectories in TBI (22). This was further supported by increased beta-band spatial variance across electrodes and greater inter-band segregation from delta activity, particularly over P3, Cz, and T5, suggesting functional reorganisation of beta activity within frontal-parietal networks.

Frequency bands power distribution retreat reveals significant changes pre- and post-retreat towards improved symmetry. This shift towards symmetry in the temporal lobes’ theta power, might indicate improved emotional processing and information encoding (63). Partial shift towards symmetry in post-retreat alpha power suggests localised improvements in emotional processing, as frontal alpha asymmetries might indicate avoidance motivation and emotional dysfunction (64). In parietal lobes, increased beta power indicates improved attention, visual processing, and multisensory integration (65).

The connectivity matrices analysed pre- and post-retreat reveal notable changes in patterns of brain connectivity, with increased connectivity strength observed particularly in the delta band, which exhibited the most pronounced post-treatment enhancement. Stronger connections emerged in frontal and central regions—areas frequently implicated in higher-order cognitive and emotional processing and overall brain stability (66). While these patterns are suggestive of enhanced neural integration, we caution that the functional significance of these changes remains speculative. Limitations such as the absence of direct connectivity metrics (e.g., phase-locking value, graph-theoretic measures) and the lack of behavioural correlations constrain our ability to definitively attribute these findings to functional improvements. As such, these connectivity changes should be considered preliminary. Future studies incorporating more granular connectivity analyses and direct links to clinical outcomes will be essential to clarify their mechanistic relevance. These patterns align with a more distributed network architecture, as indicated by the absence of distinct modular organisation or lateralisation. Representational similarity analysis (RSM) further supported this observation, revealing a convergence toward more homogeneous brain activity patterns across individuals following the retreat.

However, limitations such as the lack of specific connectivity measures and clinical context information make it challenging to definitively link observed changes to treatment effects or functional improvements. Overall, the findings suggest enhanced communication within and between crucial brain regions, potentially associated with cognitive improvements following treatment.

CCA of frequency band power revealed strong positive correlations in frontal and parietal lobes, which are critical for higher-order cognitive processes (67). The increased connectivity strength in these regions post-retreat indicated improved regulation of cognitive and emotional processes, with delta and beta bands showing the most significant enhancements in functional connectivity. The post-retreat analysis of overall network connectivity indicated substantial changes, suggesting potential improvements in integration and synchronisation within the brain network following the retreat. This enhanced integration may be associated with the observed psychological improvements, as more coordinated neural activity is crucial for effective cognitive processing and emotional regulation (68). Subtle asymmetries observed in the pre-retreat data become less apparent post-retreat, possibly indicating a greater balance in specialised functions across hemispheres.

Mechanistically, the observed changes across the paradigms investigated in this study may result from psilocybin effects on modulation of synaptogenesis, neurogenesis, neuronal plasticity, and neuroinflammation modulation (26–28, 69, p. 201). The underlying mechanisms include the upregulation of immediate early genes, like c-fos, and brain-derived neurotrophic factor (BDNF), as well as the activation of the tyrosine kinase B receptor and mammalian target of rapamycin (mTOR) signalling pathways (70–72). C-fos regulates cellular processes such as proliferation, differentiation, and survival (73–75), while BDNF is essential for neuronal transmission, survival, and synaptic plasticity (76–78). Additionally, classical hallucinogens like psilocybin activate transcription factors like Egr-1 and Egr-2 and modulate signalling through G-protein-coupled receptors, specifically Gq/11 and Gi/o (79, 80), leading to changes in protein expression.

Neuroinflammation plays a critical role in TBI, driving secondary damage via cytokine release and microglial activation. As noted by Thome et al., acute phases of TBI are characterised by profound neuroinflammation, a process that stimulates the generation and release of proinflammatory cytokines including IL-1α and IL-1β (81). Psilocybin has shown potential in mitigating this response by inhibiting lipopolysaccharide (LPS)-induced TNF-α and IL-1β production in human macrophages (82) and reducing microglial TNF-α levels in hippocampal cultures (83).

While psilocybin’s potential anti-inflammatory effects have been reported in preclinical studies, we did not measure inflammatory markers in this study; thus, such mechanisms remain speculative in our context, and should be explored in future research.

### Study limitations

Despite the promising results, several limitations must be acknowledged to provide a comprehensive understanding of this study’s findings. The relatively small sample sizes (n = 21 for EEG data, and n = 13 for psychological data) limit our power to detect changes between pre- and post-retreat performance. While the improvements observed in mental health are encouraging, a larger sample size would be necessary to confirm these findings and ensure they are representative of the broader population of veterans with TBI. Participants were recruited through the Heroic Hearts Project network, which might have introduced self-selection bias, as veterans more motivated to seek alternative treatments and with a positive attitude towards psilocybin therapy may have been more likely to participate, potentially resulting in an overestimation of the treatment’s efficacy.

Additionally, while both EEG and psychological outcomes demonstrated significant improvements, the sample with overlapping data (n = 13) was too small to support statistically reliable correlations between neural and behavioural changes. We therefore did not include exploratory correlation analyses to avoid overinterpretation or inflated Type I error, especially given the multiple comparisons inherent in EEG data. Future studies should be powered to formally assess whether specific EEG changes (e.g., delta reduction, alpha/beta modulation) predict or mediate improvements in clinical symptoms.

Additionally, we cannot determine whether the EEG changes observed post-retreat are specific to psilocybin administration or may instead reflect nonspecific factors such as natural recovery, psychological support during the retreat, structured group processes, or expectancy effects. Although the observed reductions in delta and theta power and increases in functional connectivity are consistent with psilocybin-induced changes reported in prior studies, the absence of a control group limits our ability to rule out alternative explanations. Future studies employing randomised, placebo-controlled designs are essential to isolate the neurophysiological effects attributable specifically to psilocybin.

An additional limitation relates to the exploratory subgroup analyses stratified by prior psychedelic use. While therapeutic gains were evident in both psychedelic-experienced and naïve participants, statistically significant effects were restricted to the experienced subgroup, plausibly reflecting greater sample size and reduced within-group heterogeneity. Given the very small number of psychedelic-naïve participants (n = 4), the study was underpowered to detect subtle treatment effects in this group. Consequently, definitive conclusions regarding the moderating role of prior psychedelic exposure cannot be drawn from the present data. Future studies with larger, prospectively stratified cohorts are necessary to delineate the influence of prior psychedelic experience on therapeutic efficacy.

Potential reporting biases, including demand characteristics, the Hawthorne effect, and expectancy bias, should also be considered. Participants’ awareness of being observed, their expectations of psilocybin therapy, and the desire to report positive outcomes might have influenced the results. Additionally, adverse events were not collected as part of this protocol, which limits our understanding of any potential negative effects.

The study design lacked a placebo control group, and blinding was not feasible due to the nature of the intervention (psilocybin retreats) and the observational study design. Future studies should incorporate control groups to provide a more rigorous assessment of psilocybin’s effects. A double-blind, placebo-controlled design would be ideal for isolating the effects of psilocybin without the influence of the retreat setting. The structured retreat setting, which includes comprehensive support before, during, and after psilocybin administration, must be considered a contributing factor to the overall outcomes, and it may significantly contribute to the observed therapeutic effects.

The interpretation of EEG data presents inherent challenges due to the complexity of brain activity and the potential for confounding factors. While significant changes in EEG patterns were observed post-retreat, attributing these changes solely to psilocybin without considering other retreat elements (e.g., group therapy, supportive environment) is problematic. Additionally, although post-retreat EEGs were collected at least 36 hours after the final psilocybin ceremony, residual acute effects of the psilocybin may have persisted. Confounding variables associated with the retreat setting, such as group dynamics, emotional support, and participant expectations, could have also influenced the results.

We acknowledge that the structured retreat setting, including its natural environment and supportive practices, may have contributed to the observed neurophysiological outcomes. Indeed, naturalistic settings have been associated with increased alpha power and decreased delta functional connectivity, which have been interpreted as markers of enhanced cognitive functioning and reduced stress (84, 85). As such, we cannot determine whether the EEG changes observed post-retreat are specific to psilocybin administration or may instead reflect nonspecific influences such as environmental exposure, group cohesion, psychological support, or expectancy effects. Although the reductions in delta and theta power and increases in functional connectivity are consistent with prior studies of psilocybin, the absence of a control group limits our ability to isolate drug-specific effects. Future studies employing randomised, placebo-controlled designs will be necessary to clarify the neurophysiological mechanisms underlying these changes. EEG recordings were acquired using a dry cap system, and we acknowledge the potential limitations in signal quality and artefact susceptibility compared to wet electrode systems. A longer follow-up period might strengthen future studies by providing insight into the durability of the observed effects. Intermediate behavioural assessments were not conducted during the retreat due to logistical constraints, which we acknowledge as a limitation. We recommend that future studies incorporate validated psychological measures at multiple timepoints across the intervention to better capture dynamic processes and early mediators of change. Additionally, comparative evaluations of different retreat settings and extended follow-up assessments are warranted to elucidate the long-term effects of psilocybin retreats on psychological, behavioural, cognitive, and neurofunctional outcomes.

## Conclusions

This study on veterans with psychological distress and TBI demonstrated possible benefits of psilocybin retreats. Key findings include significant reductions in depressive symptoms, PTSD, sleep disturbances, and anxiety, along with enhanced quality of life, mental well-being, and reintegration. Neurophysiological changes, indicated by EEG analysis, showed reduced delta power, normalised theta activity, and modulated alpha and beta bands, suggesting better emotional processing and cognitive control. Increased connectivity in frontal and central regions suggested improved brain communication. These results support the therapeutic potential of psilocybin retreats for improving mental health and cognitive function in veterans with TBI. However, more rigorous and controlled research is needed to investigate these findings and their attribution to the retreat setting or the substance psilocybin.

## Data Availability

The raw data supporting the conclusions of this article will be made available by the authors, without undue reservation.

## Glossary

5-HT2A: 5-Hydroxytryptamine (Serotonin) Receptor 2A
ADHD: Attention deficit hyperactivity disorder
BDNF: Brain-Derived Neurotrophic Factor
CCA: Canonical Correlation Analysis
CNS: Central Nervous System
CNSVS: CNS Vital Signs
EEG: Electroencephalography
Egr-1: Early Growth Response 1
Egr-2: Early Growth Response 2
GCS: Glasgow Coma Scale
IL-1α: Interleukin-1 alpha
IL-1β: Interleukin-1 beta
LPS: Lipopolysaccharide
M2C: Military to Civilian Questionnaire
MDD: Major Depressive Disorder
mTBI: Mild Traumatic Brain Injury
mTOR: Mammalian Target of Rapamycin
PCL-5: PTSD Checklist
PHQ-9: Patient Health Questionnaire for Depression
PME: Psychedelic Mushroom Extract
PROMIS-SD: Patient-Reported Outcomes Measurement Information System Sleep Disturbance Short Form
PTSD: Post-Traumatic Stress Disorder
Qolibri-OS: Quality of Life After Brain Injury - Overall Scale
RDM: Representational Dissimilarity Matrix
RPQ: Rivermead Post-Concussive Questionnaire
RSM: Representational Similarity Matrix
SD: Standard Deviation
STAI: State-Trait Anxiety Inventory
TBI: Traumatic Brain Injury
TNF-α: Tumor Necrosis Factor alpha
USD: United States Dollars
W-EMWS: The Warwick-Edinburgh Mental Well-being Scale.
