# Supplementary material for: Improved mental health outcomes and normalised spontaneous EEG activity in veterans reporting a history of traumatic brain injuries following participation in a psilocybin retreat
Source: Front Psychiatry. 2025 Aug 6;16:1594307. doi: 10.3389/fpsyt.2025.1594307 (PMC12364870; doi:10.3389/fpsyt.2025.1594307)
Supplement: Supplementary file 1 [file Supplementaryfile1.docx]

# **Supplementary Material 1: Pre-Post Treatment Analysis Controlling for Prior Psychedelic Use**

# 1. Rationale and Methodology

In response to the reviewer’s concern regarding prior psychedelic use as a potential confounder, we reanalyzed the primary outcomes by stratifying participants based on self-reported psychedelic experience. Participants (N = 13) were initially categorized using five levels of prior usage:

- “Never” (N = 4)
- “Only once” (N = 1)
- “2–5 times” (N = 4)
- “21–50 times” (N = 3)
- “More than 100 times” (N = 1)

To preserve statistical power and ensure meaningful group comparisons given the small sample size, we applied a binary grouping strategy: individuals reporting “Never” were labeled as psychedelic-naïve (N = 4), while all others were labeled as psychedelic-experienced (N = 9). This dichotomization balances the need for interpretability and feasibility under limited sample constraints. All variables were reanalyzed using paired t-tests within each subgroup.

# 2. Updated Results by Prior Psychedelic Use

The figure attached summarizes violin plots and within-subject pre/post trajectories for each outcome stratified by prior psychedelic use (Yes/No). The key findings are:

## 2.1. PHQ-9 (Depression Severity)


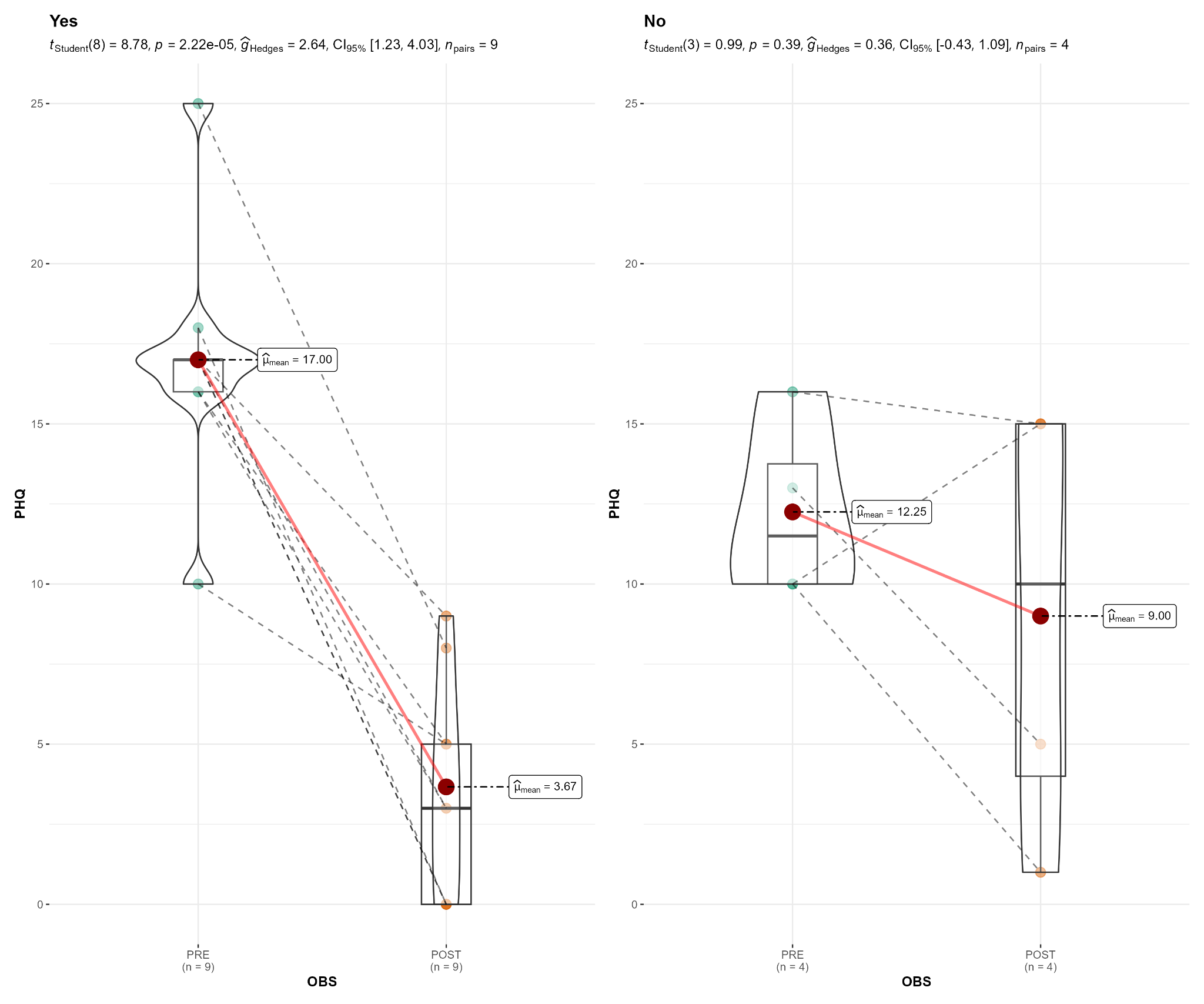


When analyzing PHQ-9 scores, the subgroup with prior psychedelic use demonstrated a highly significant reduction in depressive symptoms post-treatment (t(8) = 8.78, p = 2.22e–05), with a large effect size (Hedges’ g = 2.64, 95% CI [1.23, 4.03]). In contrast, while the psychedelic-naïve group also showed a numerical reduction in scores from pre- to post-treatment (mean = 12.25 to 9.00), this difference did not reach statistical significance (t(3) = 0.99, p = 0.39), and the effect size was small (Hedges’ g = 0.36, 95% CI [–0.43, 1.09]).

##

##

## 2.2. QOLIBRI-OS (Quality of Life After Brain Injury)

**
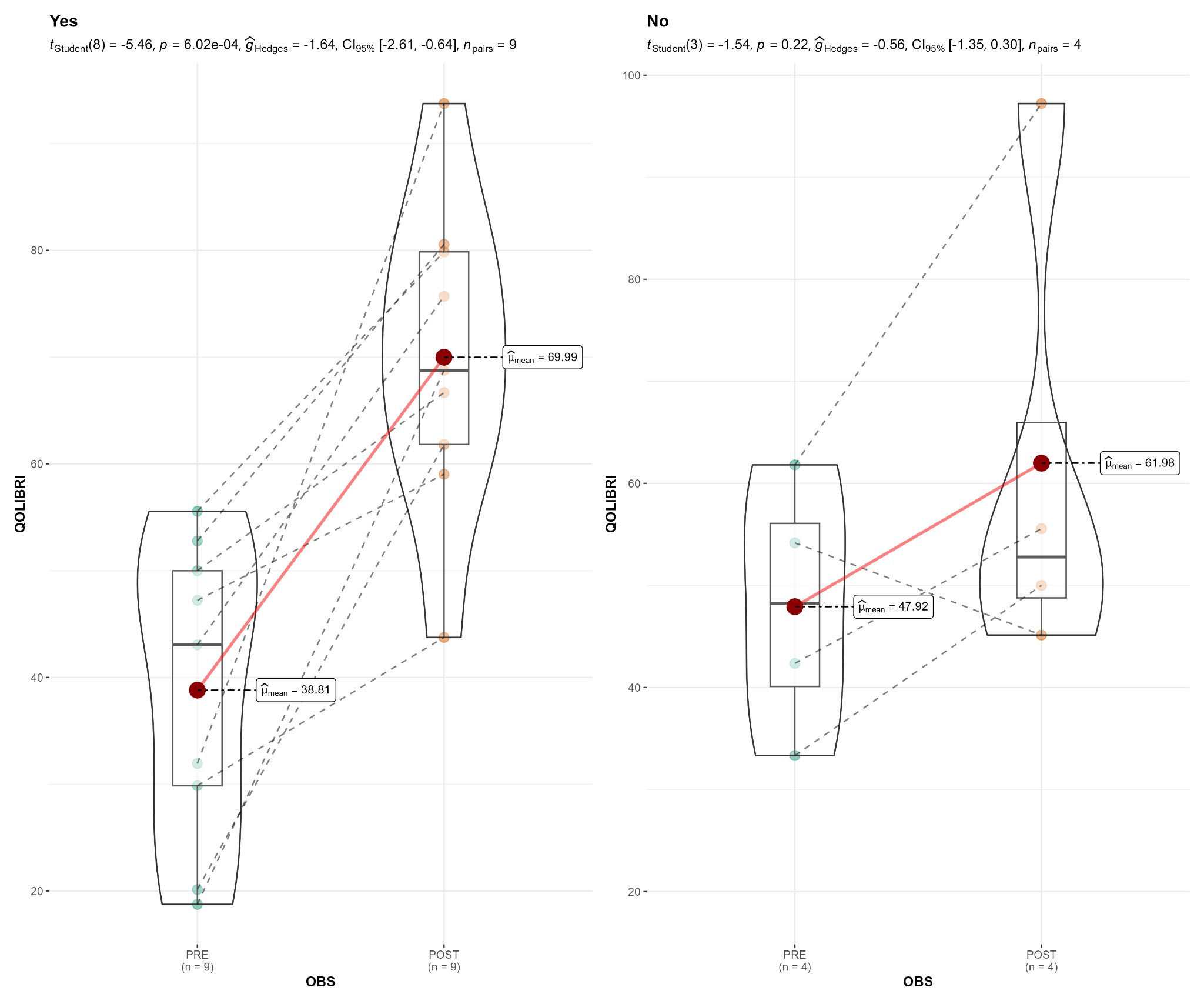
**
When analyzing QOLIBRI scores, the subgroup with prior psychedelic use showed a significant increase in quality of life following treatment (t(8) = -5.46, p = 6.02e–04), with a large effect size (Hedges’ g = -1.64, 95% CI [–2.61, –0.64]). In contrast, although the psychedelic-naïve group also showed a numerical increase in QOLIBRI scores from pre- to post-treatment (mean = 47.92 to 61.98), the change was not statistically significant (t(3) = -1.54, p = 0.22), and the effect size was moderate and imprecise (Hedges’ g = -0.56, 95% CI [–1.35, 0.30]).

##

##

##

##

##

##

## 2.3. Rivermead (Post-Concussion Symptoms)

**
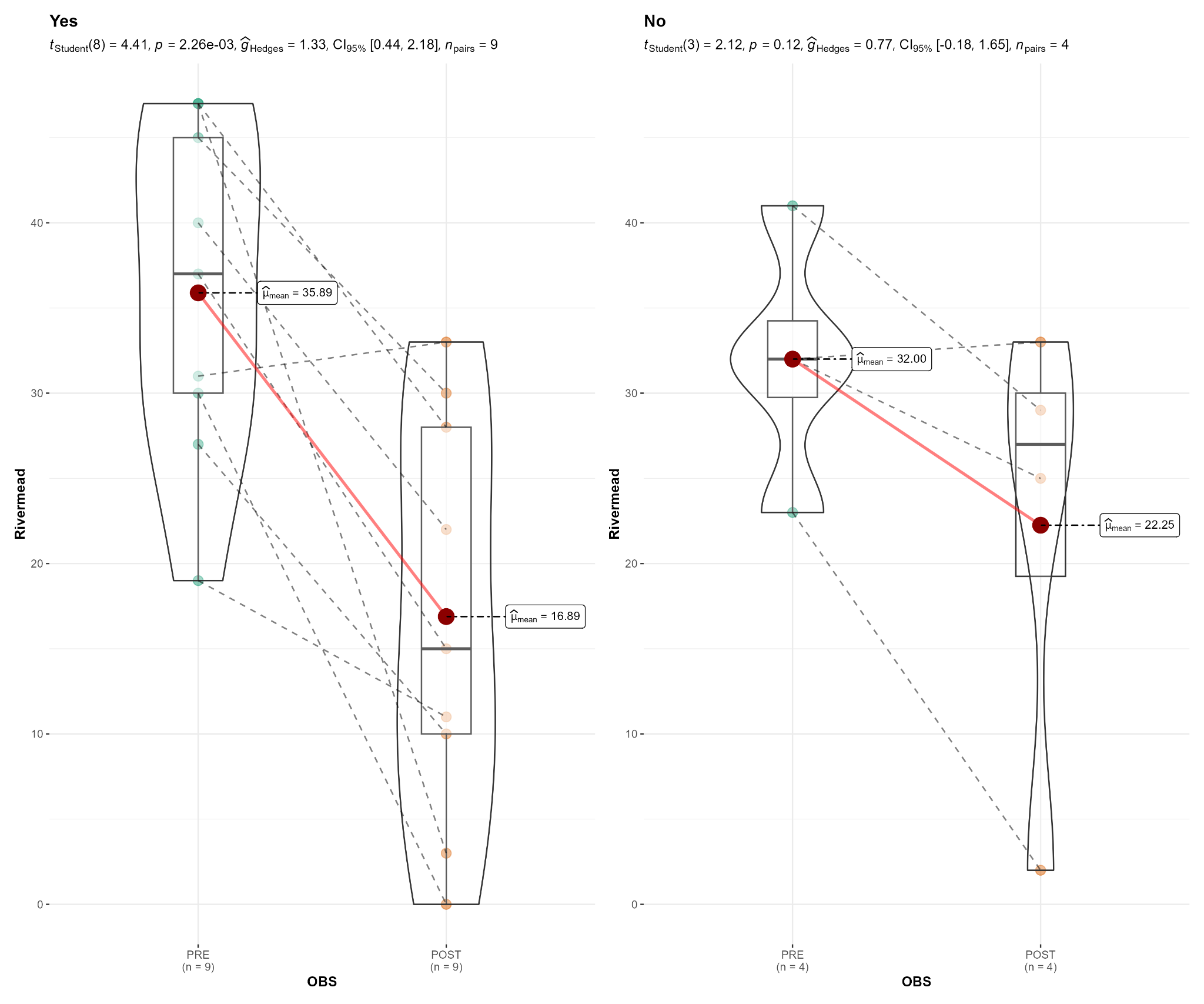
**

When analyzing Rivermead scores, the subgroup with prior psychedelic use demonstrated a significant reduction in post-concussion symptoms following treatment (t(8) = 4.41, p = 2.26e–03), with a large effect size (Hedges’ g = 1.33, 95% CI [0.44, 2.18]). In contrast, while the psychedelic-naïve group also showed a decrease in scores from pre- to post-treatment (mean = 32.00 to 22.25), the difference did not reach statistical significance (t(3) = 2.12, p = 0.12), and the effect size was moderate (Hedges’ g = 0.77, 95% CI [–0.18, 1.65]).

###

###

###

###

## 2.4. PCL-5 (PTSD Checklist)

**
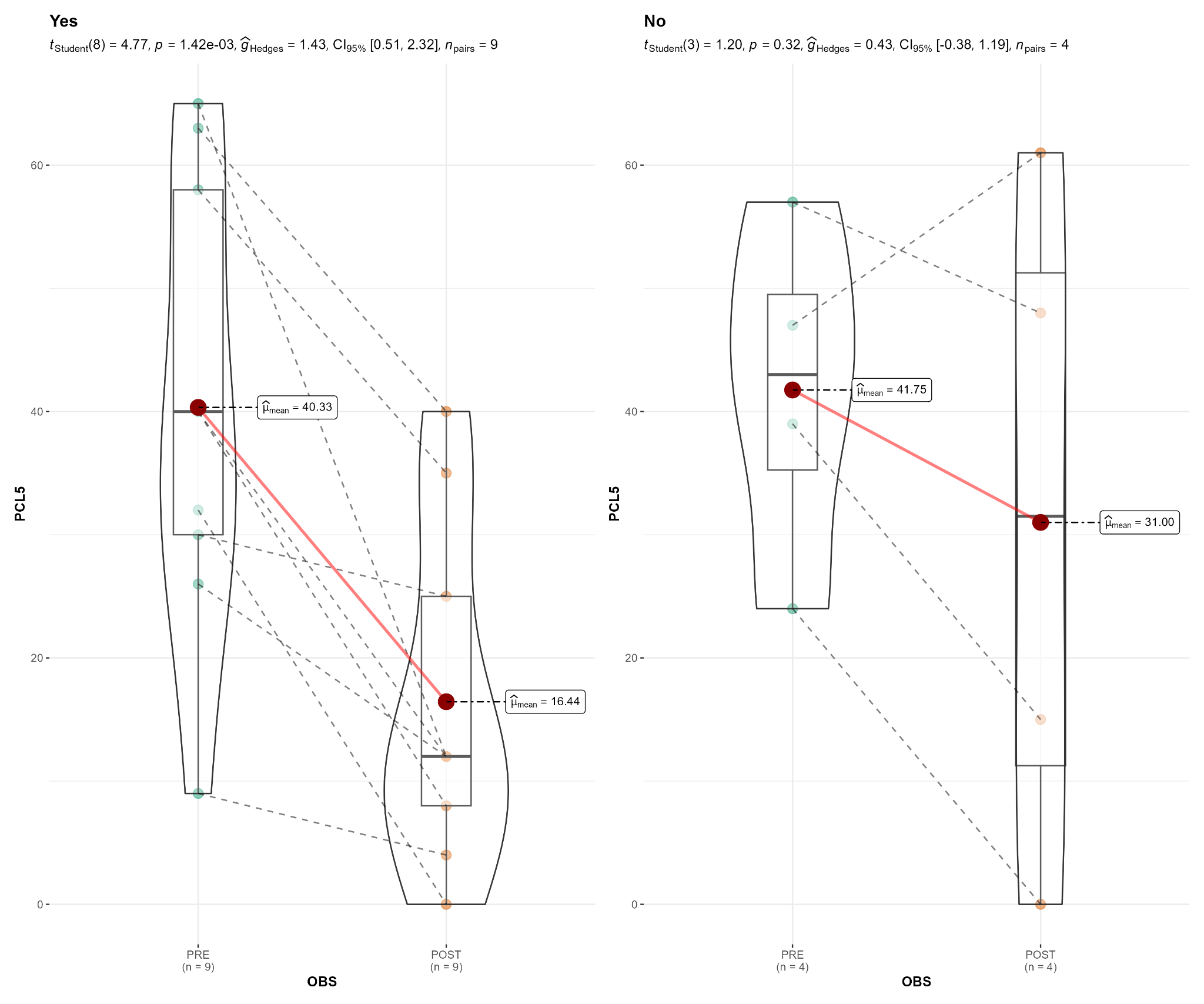
**
When analyzing PCL-5 scores, the subgroup with prior psychedelic use exhibited a significant reduction in PTSD symptoms following treatment (t(8) = 4.77, p = 1.42e–03), with a large effect size (Hedges’ g = 1.43, 95% CI [0.51, 2.32]). In contrast, the psychedelic-naïve group showed a numerical decrease in scores (mean = 41.75 to 31.00), but this change was not statistically significant (t(3) = 1.20, p = 0.32), and the effect size was small (Hedges’ g = 0.43, 95% CI [–0.38, 1.19]).

###

###

###

###

##

##

## 2.5. PROMIS Raw (Sleep Disturbance Short Form)


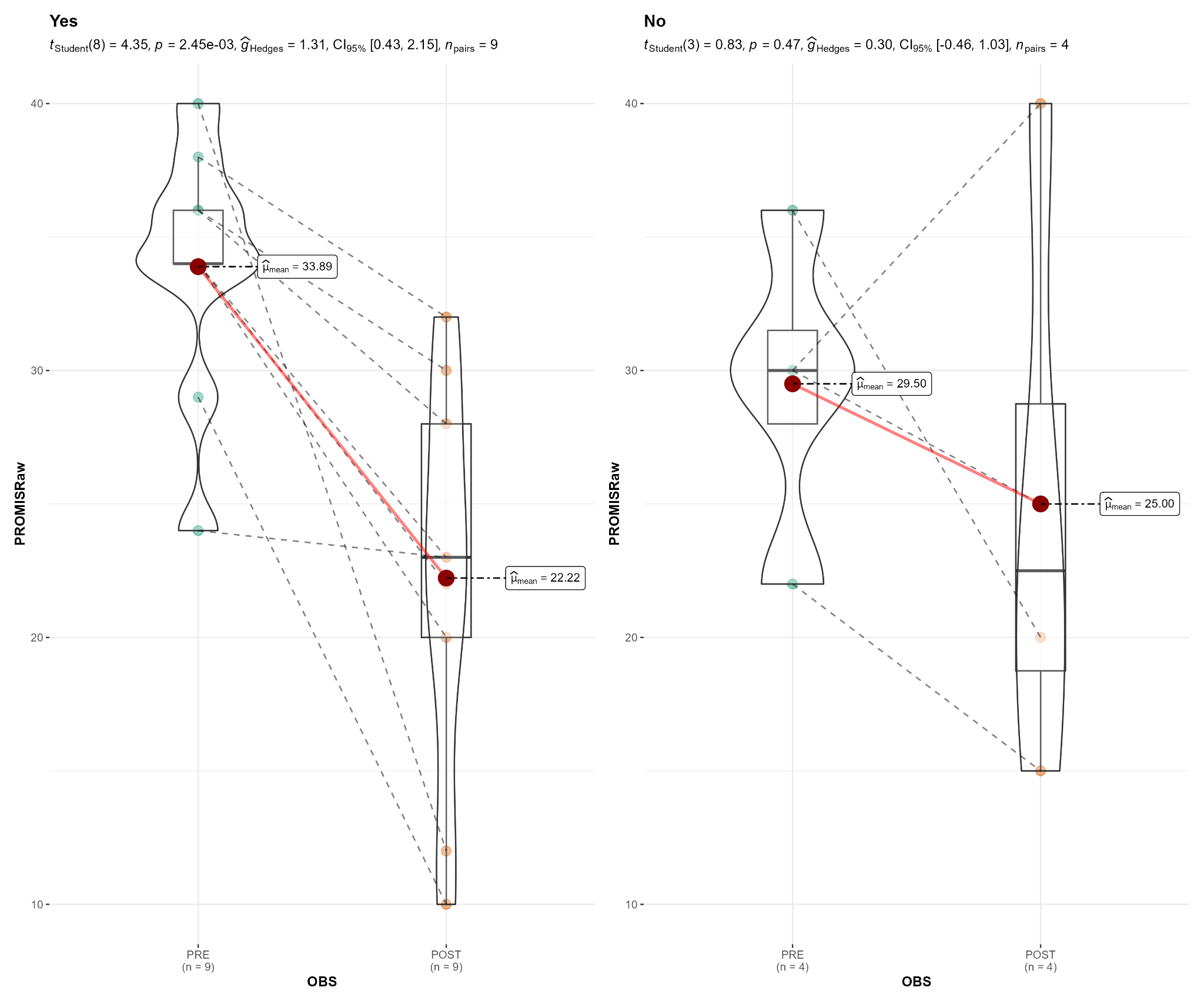


When analyzing PROMIS Raw scores, the subgroup with prior psychedelic use showed a significant reduction in emotional distress post-treatment (t(8) = 4.35, p = 2.45e–03), with a large effect size (Hedges’ g = 1.31, 95% CI [0.43, 2.15]). In contrast, the psychedelic-naïve group also showed a decrease in scores (mean = 29.50 to 25.00), but this change was not statistically significant (t(3) = 0.83, p = 0.47), and the effect size was small (Hedges’ g = 0.30, 95% CI [–0.46, 1.03]).

###

###

###

###

###

###

### 2.5.1 PROMIS T-Score (Sleep Disturbance Short Form)


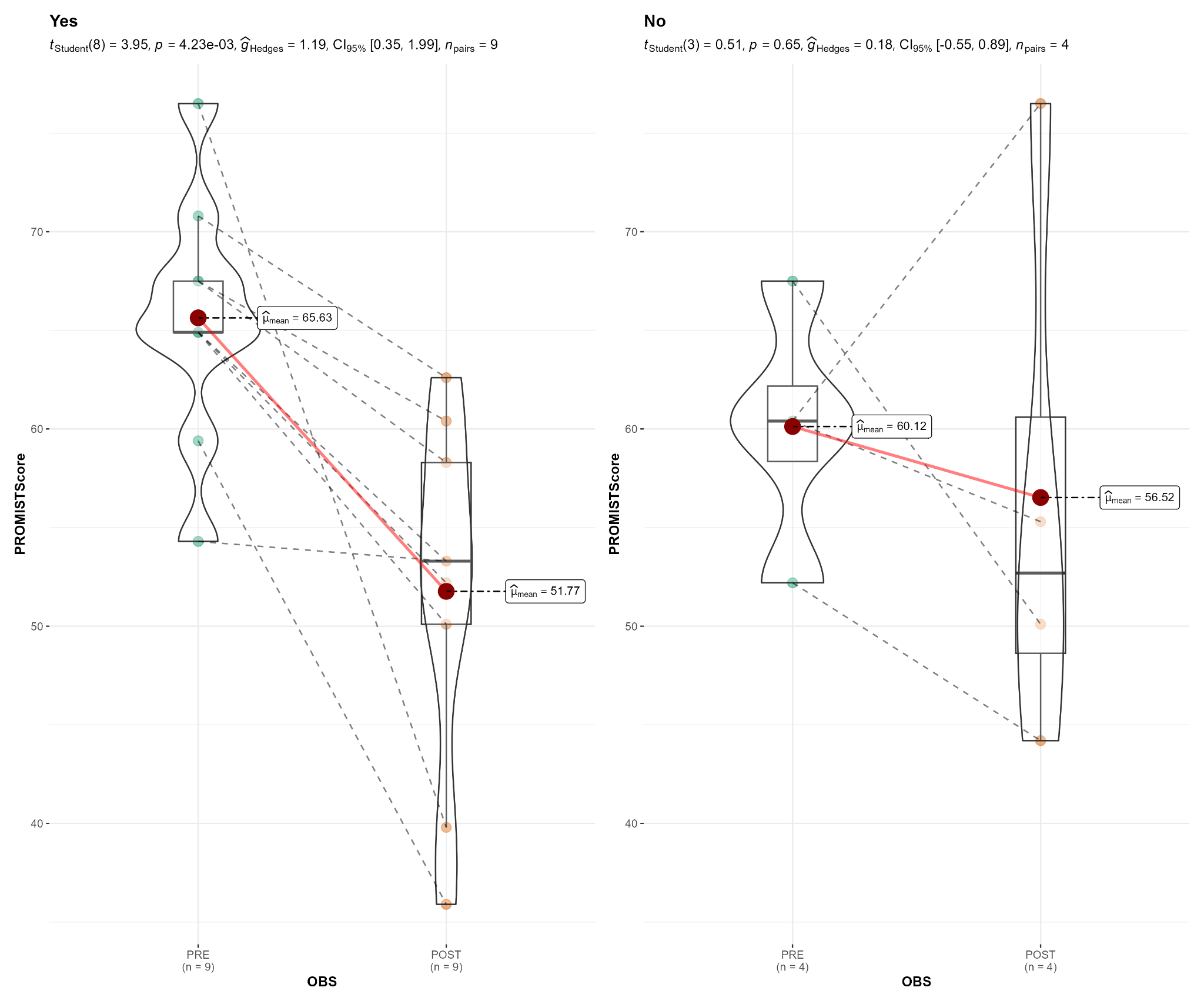


###

When analyzing PROMIS T-scores, the subgroup with prior psychedelic use demonstrated a significant reduction in symptom burden post-treatment (t(8) = 3.95, p = 4.23e–03), with a large effect size (Hedges’ g = 1.19, 95% CI [0.35, 1.99]). In contrast, the psychedelic-naïve group showed only a modest decrease in scores (mean = 60.12 to 56.52), which was not statistically significant (t(3) = 0.51, p = 0.65), and the effect size was very small (Hedges’ g = 0.18, 95% CI [–0.55, 0.89]).

###

###

###

###

###

## 2.6. sWEMWBS Raw (The Warwick – Edinburgh Mental Well-being Scale)


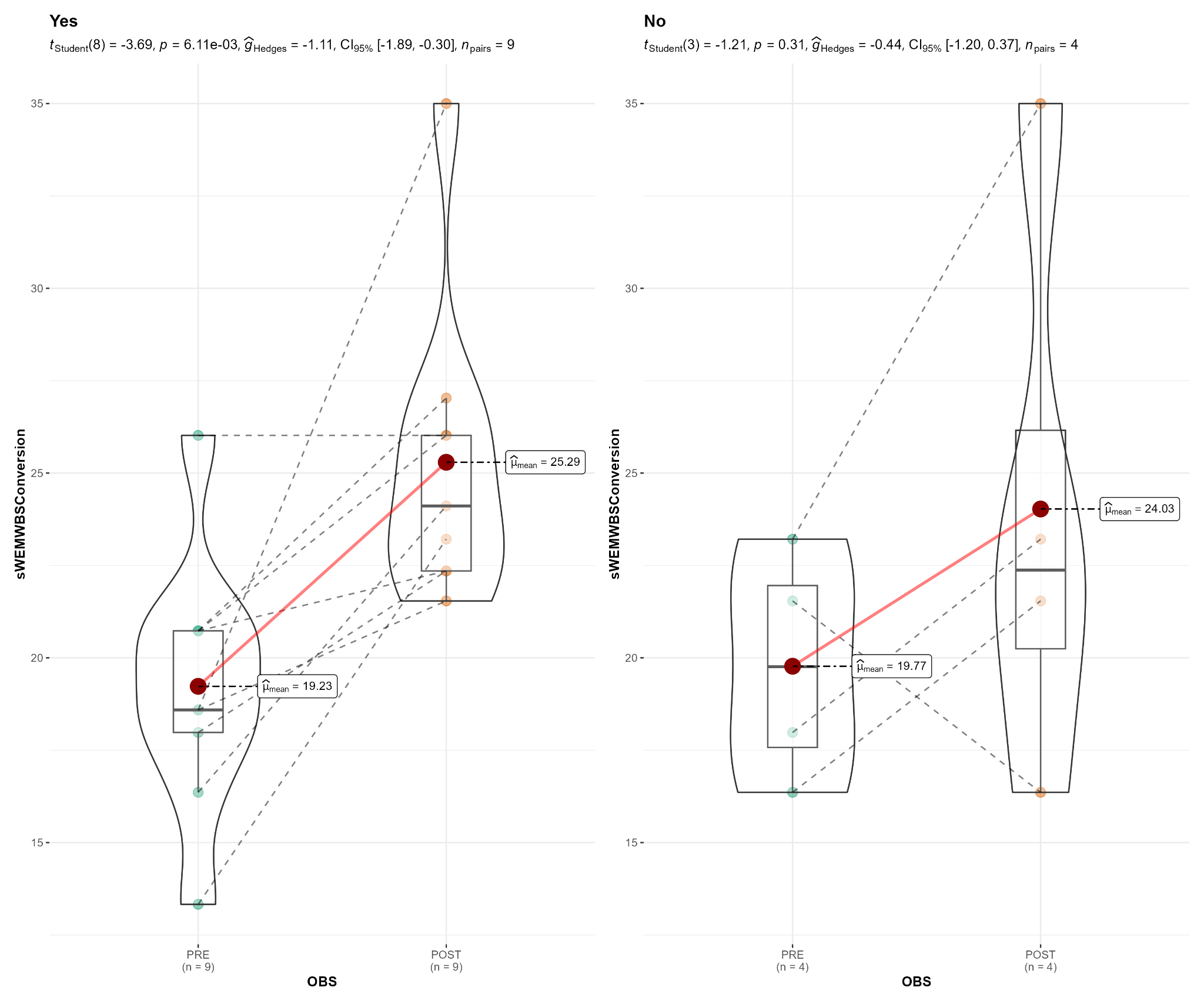


When analyzing sWEMWBS Conversion scores, the subgroup with prior psychedelic use showed a significant increase in wellbeing post-treatment (t(8) = -3.69, p = 6.11e–03), with a large effect size (Hedges’ g = -1.11, 95% CI [–1.89, –0.30]). In contrast, the psychedelic-naïve group also exhibited a numerical increase in scores (mean = 19.77 to 24.03), but this change was not statistically significant (t(3) = -1.21, p = 0.31), and the effect size was small (Hedges’ g = -0.44, 95% CI [–1.20, 0.37]).

###

###

###

###

###

### 2.6.1 sWEMWBS Conversion (The Warwick – Edinburgh Mental Well-being Scale)

### **
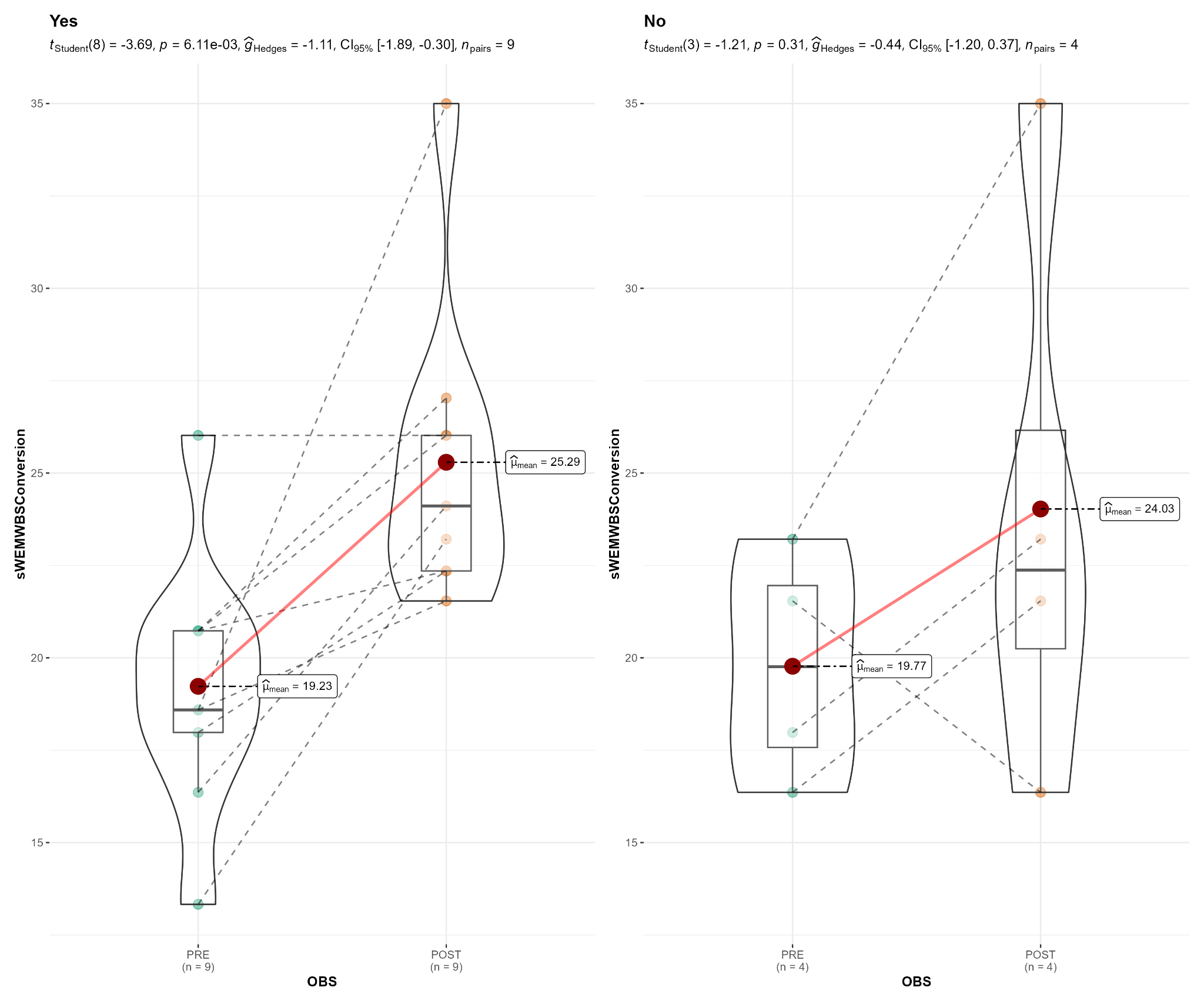
**

###

When analyzing sWEMWBS Conversion scores, the subgroup with prior psychedelic use exhibited a significant increase in wellbeing following treatment (t(8) = -3.69, p = 6.11e–03), with a large effect size (Hedges’ g = -1.11, 95% CI [–1.89, –0.30]). In contrast, the psychedelic-naïve group showed a numerical increase in scores (mean = 19.77 to 24.03), but this change was not statistically significant (t(3) = -1.21, p = 0.31), and the effect size was small (Hedges’ g = -0.44, 95% CI [–1.20, 0.37]).

###

###

###

## 2.7. STAIT (Anxiety)


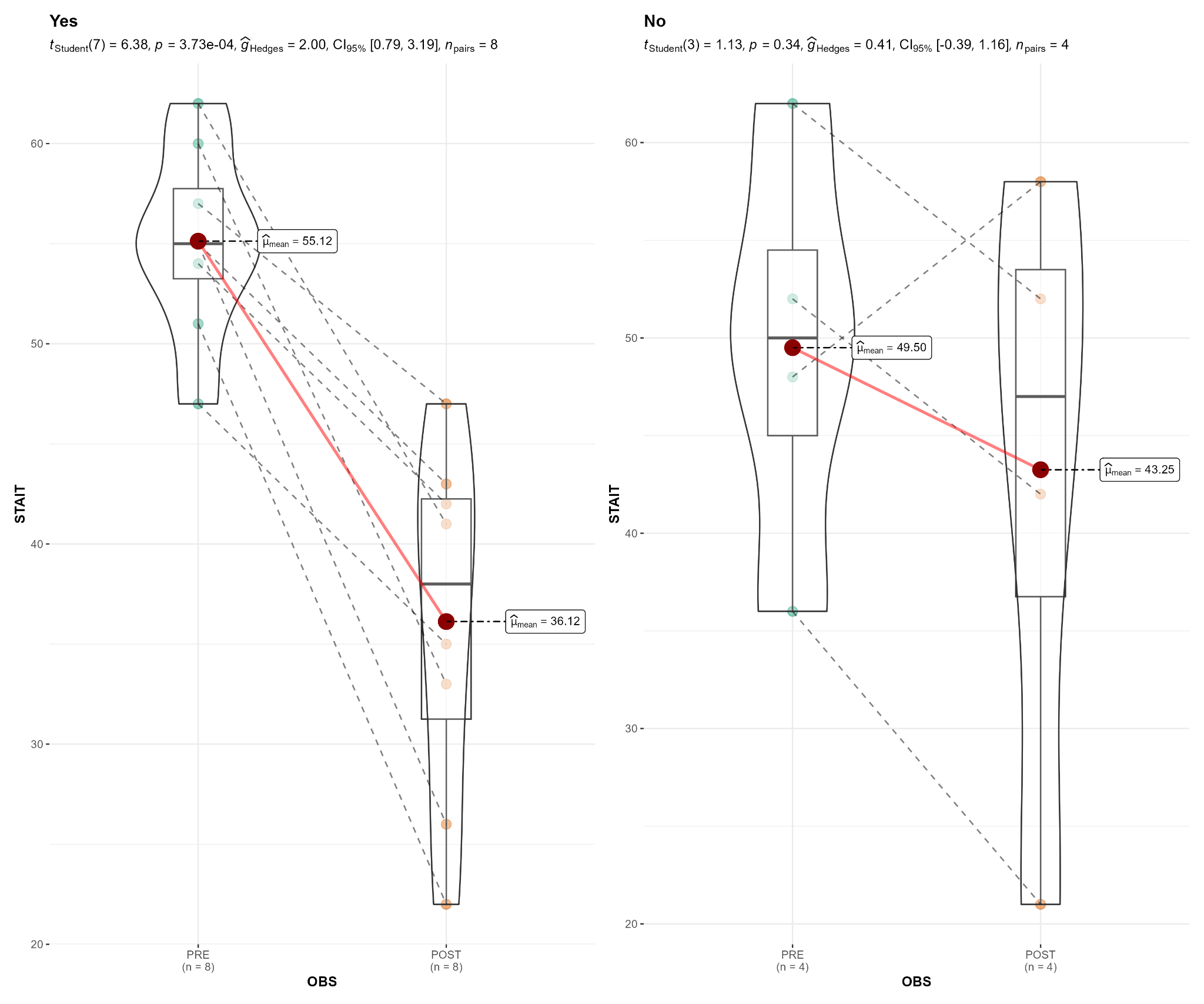


When analyzing STAIT scores, the subgroup with prior psychedelic use showed a significant reduction in anxiety levels post-treatment (t(7) = 6.38, p = 3.73e–04), with a very large effect size (Hedges’ g = 2.00, 95% CI [0.79, 3.19]). In contrast, while the psychedelic-naïve group also showed a numerical reduction in anxiety (mean = 49.50 to 43.25), this change was not statistically significant (t(3) = 1.13, p = 0.34), and the effect size was small (Hedges’ g = 0.41, 95% CI [–0.39, 1.16]).

###

###

###

##

##

## 2.8. M2C (Military to civilian questionnaire)

**
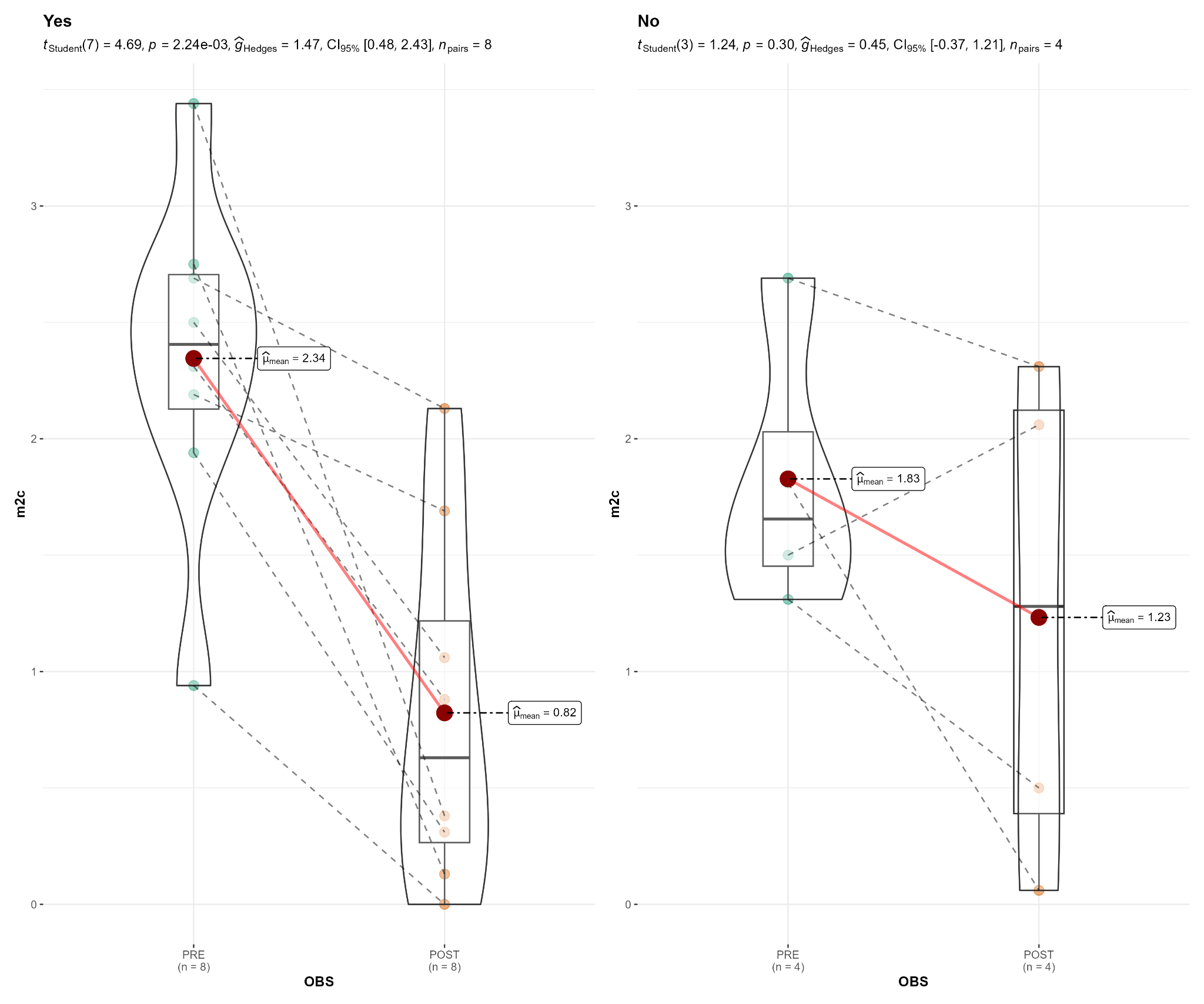
**

When analyzing M2C scores, the subgroup with prior psychedelic use showed a significant reduction in cognitive complaints post-treatment (t(7) = 4.69, p = 2.24e–03), with a large effect size (Hedges’ g = 1.47, 95% CI [0.48, 2.43]). In contrast, the psychedelic-naïve group also showed a numerical decrease in scores (mean = 1.83 to 1.23), but this change was not statistically significant (t(3) = 1.24, p = 0.30), and the effect size was small (Hedges’ g = 0.45, 95% CI [–0.37, 1.21]).

##

##

##

# 3. Summary of Differences Compared to Primary Analysis

| Metric | Significant in Original? | Still Significant (Psychedelics experienced)? | Significant (Psychedelics naïve)? |
| --- | --- | --- | --- |
| **PHQ-9** | Yes | Yes | No |
| **QOLIBRI** | Yes | Yes | No |
| **Rivermead** | Yes | Yes | No |
| **PCL-5** | No (adj.) | Yes | No |
| **PROMIS Raw** | Yes | Yes | No |
| **PROMIS T** | No (adj.) | Yes | No |
| **sWEMWBS Raw** | No (adj.) | Yes | No |
| **sWEMWBS Conversion** | No (adj.) | Yes | No |
| **STAIT** | Yes | Yes | No |
| **M2C** | Yes | Yes | No |

# 4. Discussion and Limitations

These stratified sub-analyses suggest that the therapeutic benefits of psilocybin remain evident in both psychedelic-naïve and experienced individuals, though significance levels are observed in the experienced group only, possibly due to larger sample size and reduced inter-individual variance. The small number of naïve participants (N = 4) poses an important limitation. Despite consistent trends in outcomes such as PHQ-9, M2C, and STAIT, statistical power remains insufficient to detect all effects reliably in this subgroup. Future work with larger sample sizes is warranted to more precisely characterize whether prior psychedelic exposure modulates baseline neuropsychological profiles or treatment responsiveness.
