# Supplementary material for: Improved mental health outcomes and normalised spontaneous EEG activity in veterans reporting a history of traumatic brain injuries following participation in a psilocybin retreat
Source: Front Psychiatry. 2025 Aug 6;16:1594307. doi: 10.3389/fpsyt.2025.1594307 (PMC12364870; doi:10.3389/fpsyt.2025.1594307)

Supplementary Material 2: EEG Analyses Details

# Methods

## Statistical Analyses

To quantify pre- to post-retreat changes in neural activity, statistical comparisons were performed on band-specific EEG measures across canonical frequency bands (delta, theta, alpha, beta). Paired, non-parametric Wilcoxon signed-rank tests were used to assess differences in both spectral power and variance across electrodes within each frequency band. To evaluate inter-band distinctions, pairwise comparisons were conducted between delta and each of the higher frequency bands, as well as between theta and alpha, within each subject. P-values were transformed using the negative base-10 logarithm (–log₁₀(p)) for interpretability, with values exceeding 1.3 indicating statistical significance at p < 0.05. False discovery rate (FDR) correction was applied to control for multiple comparisons across electrodes. This approach enabled spatially resolved identification of significant changes in oscillatory dynamics and enhanced network differentiation following psilocybin administration.

## Electrode Selection

The EEG recordings were stored in EDF format and consisted of 25 channels. Five of these channels were dropped as they had no use in the analysis. The dropped channels included CM, X1, X2, X3, and Trigger. Electrodes included in the analysis were from the following regions: parietal (P3, P4), central (Cz, C3, C4), temporal (T3, T4, T5, T6), frontal (F3, F4, F7, F8), frontopolar (Fp1, Fp2), and those placed on the ear (A1, A2).

## Preprocessing

Regardless of the lengths of each recording, the first and last 15 seconds of the recordings were trimmed, resulting in recordings of various lengths. **Figure S1** shows the distribution of resulting recording lengths (duration) in the pre-retreat and post-retreat recordings after trimming. All recordings reported a line frequency of zero, as shown in **Figure S2**, but the power spectral density plots of recordings show the presence of 50Hz line frequency and its harmonics in some recordings (**Figure S4a-4b**).

As depicted in **Figure S3**, all recordings had a sampling frequency of 300Hz, making the useful information content upper-bounded by 150Hz. **Figure S3** also shows that all recordings implemented bandpass filtering by using both a lowpass and high pass filter. Some recordings used a low pass cutoff of 150 Hz and a high pass of 0Hz, while others used a low pass cutoff of 50Hz and a high pass cutoff of 0Hz.

Since no specific phenomenon in the higher beta band (>30Hz) is required for this analysis (given that it is a rest state EEG and our interest is in lower frequency bands) and due to the presence of 50Hz line noise and harmonics in some recordings, a high pass and lowpass filter with cutoffs of 1Hz and 35Hz were applied to each trimmed recording. **Figure S4a-4b** show the power spectral density of the recordings prior to the 1-35 Hz bandpass filtering, and **Figure S4c-4d** show the power spectral density of the recordings after 1-35 Hz bandpass filtering.

There was no need for re-referencing as the EEG recordings were already referenced to Pz.

## Features Computation & Analysis

After preprocessing, the power of the frequency bands of interest was computed, the coherence of the recordings was also computed, and the time-frequency representation of the recordings was obtained via the short-time Fourier transform.

## Bands Power

Frequency bands of interest are in the lower range of 1-30Hz, as each recording is taken during simulated sleep or a restful state of subjects. Four bands are of interest in this frequency range: the delta band (1-4Hz), theta band (4-8Hz), alpha band (8-12Hz), and beta band (12-30Hz). The Welch spectrum was computed for each recording, and the mean of the powers in the frequency range of each band resulted in the various bands' power (delta, theta, alpha, and beta).

In analysing the frequency bands' power, the internal correlations between the bands' power of the pre-retreat were computed, as well as the post-retreat. Significant changes in the correlation of the delta band powers were noticed, as shown in **Figure S5**, and its implications are discussed further in the results section.

To investigate the relationship and differences between the bands' power of the pre-retreat and post-retreat recordings, canonical correlation analysis was carried out between the two, with the pre-retreat bands' power acting as the independent variable and the post-retreat bands' power as the target variable. Again, significant changes in the pre-retreat and post-retreat canonical weights were noticed, particularly in the delta band, as shown in **Figure S6**, and the implications are also discussed in the results section.

Lastly, the montage of the bands' power and the distribution of the logarithm of the bands' power show notable differences between the two groups, indicative of improvement due to treatment. This is illustrated in **Figure S7** and **Figures S8a-8d**, respectively.

## Coherence

The frequency coherence between electrodes was computed within the 1-30 Hz frequency range of interest. To facilitate analysis, coherence values were converted to the decibel scale and then categorised into the delta, theta, alpha, and beta bands by computing the mean of the coherence values for the frequency range of each band. The resulting bands' coherence was scaled between zero and one, aiming to simplify the interpretation of resultant data and plots.

In analysing the frequency bands' coherence, the average of the pre- and post-retreat coherence for each band was computed. Heatmaps and connectivity circles were employed for interpretation. The heatmaps and connectivity circles are presented in **Figure S9** and **Figure S10**, respectively.

## Short Time Fourier Transform

The short-time Fourier transform was computed for each electrode in each recording, resulting in a time-frequency plot for each recording. The frequencies of interest and their activity over time were extracted from the time-frequency arrays and further processed to enhance ease of analysis. In analysing the resulting time-frequency spectrograms, representation similarity analysis was employed, using cosine similarity as the measure. Each 2D spectrogram from each recording was flattened, and the cosine similarity between every pair of pre- and post-retreat resultant vectors was computed. The same procedure was applied for pairs of pre-retreat recordings and pairs of post-retreat recordings. The results of this analysis were visualised in heatmaps known as the representational similarity matrix (RSM).

Before conducting representational similarity analysis, the time axes of the spectrograms were resampled due to their varying lengths across recordings, resulting from the varying lengths of the recordings themselves. **Figure S11** shows the RSM for the various pairs considered.

## Canonical Correlation Analysis as a Multivariate Time-Series Analysis Method

Canonical Correlation Analysis (CCA) was employed on the recordings to comprehend the correlation structure. For every pre-retreat recording, the canonical weight was computed from the CCA between that specific pre-retreat recording and all post-retreat recordings. The mean of the independent variable canonical weights provided the resultant weights for that pre-retreat recording, and the mean of the dependent variable (post-retreat) canonical weights served as the resultant weight that described all post-retreat recordings. Essentially, this analysis served to compare each pre-retreat recording to all post-retreat recordings. **Figure S12a-12b** shows the results of this analysis, revealing a repeated structure in the post-retreat weights. Further analysis and implications are provided in the results section.

# Results

## Frequency Bands Correlations

In order to study specifically the changes in EEG band correlations for TBI patients before and after treatment. By examining the intricate dance of delta, theta, alpha, and beta waves across different brain regions, we can glean valuable insights into the treatment's impact on their neural networks. Prior to treatment, a symphony of strong correlations dominates the scene. The delta band, associated with deep sleep and unconsciousness, seems tightly intertwined with the activity in other bands, particularly the alpha band linked to relaxation and attention. This suggests a dysfunctional interplay between processing external stimuli and internal states among the subjects prior to the retreat treatment, potentially reflecting ongoing cognitive struggles. In the post-treatment recordings, the tide turns. The correlations weaken, hinting at a loosening of the rigid grip that delta once held. Theta waves, crucial for learning and memory consolidation, begin to forge new connections with the alpha band, suggesting a shift towards enhanced cognitive processing and integration. Beta waves, the orchestra conductors of focused attention, also show reduced correlations with delta, potentially indicating improved ability to filter out distractions and maintain vigilance. These changes paint a promising picture. The treatment appears to be dismantling the maladaptive connections that may have hampered cognitive function and replacing them with a more balanced interplay between brainwaves. This newfound freedom allows for improved information processing, attention, and potentially, memory consolidation.

## Frequency Bands Headplots

Pre-treatment assessments revealed widespread overactivity across all frequency bands, particularly in the frontal, temporal, and parietal lobes, indicative of disrupted cognitive function commonly observed in TBI patients.

Upon post-treatment analysis, notable changes were observed, suggesting positive effects on emotional regulation and cognitive processing. Reductions in delta power, particularly in frontal and temporal regions, were identified, signalling potential improvements in emotional regulation and processing control. The normalisation of abnormal theta activity in temporal lobes post-treatment suggested enhanced identification and understanding of emotional cues. Modulation of alpha power, specifically decreased activity in frontal and central regions, indicated heightened attention and improved cognitive processing directed towards emotional information.

Further analysis of the beta band revealed increased power in frontal and central regions post-treatment, suggesting enhanced cognitive engagement and processing efficiency, particularly for emotional stimuli. While these findings indicated positive responses to neuromodulation treatment, it is noteworthy that some regions, particularly in the temporal lobes, still exhibited evidence of overactivity post-treatment.

In conclusion, our quantitative EEG analysis provides valuable insights into the neural changes associated with neuromodulation treatment in TBI patients. The observed reductions in delta power, normalisation of theta activity, and modulation of alpha and beta bands suggest potential improvements in emotional information processing and cognitive control. However, the persistence of overactivity in certain regions warrants further investigation to optimise treatment strategies and address lingering challenges in emotional and cognitive function post-TBI.

## Frequency Bands Power Distribution

Analysing decibel power across frequency bands before and after treatment reveals significant changes. Wilcoxon signed-rank tests revealed significant variance increases in theta, alpha, and beta bands after the retreat, with –log₁₀(p) values of 5.38, 6.00, and 6.00, corresponding to approximate p-values of 4.17 × 10⁻⁶, 1.00 × 10⁻⁶, and 1.00 × 10⁻⁶, respectively. The delta band displayed a marginal effect, with a –log₁₀(p) of 1.41 (p ≈ 0.039). These results are shown in **Figure S13.**

Violin plots of the log-scaled variance distributions (**Figure S14**) further illustrate these differences. Post-treatment, the theta, alpha, and beta bands exhibit broader distributions and upward shifts in variance compared to the pre-treatment recordings, consistent with increased spatial heterogeneity in power expression. In contrast, the delta band variance remains tightly constrained.

To further investigate the divergence between bands, electrode-wise Wilcoxon signed-rank tests were performed comparing delta band power to theta, alpha, and beta bands, both pre- and post-treatment. The results, shown in **Figures S15-18**, reveal significant increases in inter-band distinctiveness following treatment. Specifically, post-retreat comparisons between delta and each of the higher-frequency bands show multiple electrodes with FDR-corrected –log₁₀(p) values exceeding 1.3, particularly in P3, C3, C4, P4, Cz, A1, T5, F8, and A2. These spatially distributed effects suggest enhanced differentiation of band activity in centro-parietal and temporal regions.

In contrast, comparisons between theta and alpha band power (**Figure S18**), despite reductions in correlation observed post-treatment, apart from electrode T5 and P4, did not show widespread significant divergence across electrodes. This indicates that while functional coupling between theta and alpha bands may have weakened, their spatial power distributions remained similar after the intervention.

In the delta band, there is a shift from widespread pretreatment power in the temporal region to post-treatment symmetry, suggesting improved emotional processing and cognitive functions. Higher delta power in the Cz region indicates focus and information processing deficits, which reduce post-treatment, implying enhanced attention control. The change in delta power in the parietal lobes signifies improved stimuli selection, spatial awareness, visual processing, and sensory integration, potentially leading to enhanced multisensory processing.

Notably, Cz exhibits similar pre- and post-treatment patterns in the theta band, suggesting subtler or more gradual effects on attention, cognitive processing, and emotional regulation compared to delta band changes. The shift towards symmetry in the temporal lobes' theta power indicates improved emotional processing and information encoding, albeit to a lesser extent compared to delta, possibly implying slower or more complex developments. The parietal lobes show no distinct pre- and post-treatment differences in theta power, suggesting a focus on other frequency bands or involvement in cognitive functions beyond theta activity. Finally, the convergence of post-treatment theta power in central electrodes C3 and C4 suggests enhanced synchronisation and coordination, potentially improving cognitive processing efficiency and information exchange in those regions.

At Cz, the subtle effects on attention and cognitive processing are indicated by minimal changes in alpha power compared to delta and theta. In the temporal lobes, a partial shift towards symmetry in post-treatment alpha power, particularly in T3 and T5, suggests localised improvements in emotional processing. Parietal lobes show no distinct pre- and post-treatment alpha power differences, possibly reflecting focus on other bands or involvement in functions like multisensory integration. Widespread post-treatment alpha power distribution in P3 may signify increased cognitive engagement. Frontal lobes exhibit minimal changes in alpha power, but preliminary signs of improved cognitive control and attention regulation in Fz and F4 are noted, warranting further analysis of specific tasks related to these functions.

In the central region (Cz), subtle cognitive improvements are indicated by stable beta power levels compared to other bands. Analysing information processing or motor control tasks at Cz post-treatment could provide more insights. In frontal regions, the shift from widespread to symmetrical beta power suggests enhanced cognitive control, attention, and information processing efficiency after treatment. Exceptions highlight regional specificity. In parietal lobes, increased beta power indicates improved attention, visual processing, and multisensory integration after treatment. Overlap in P3 signals individual variability in attentional impact. In temporal lobes, minimal symmetry shifts suggest slower developments in emotional and auditory processing.

Higher T6 beta power post-treatment may reflect increased engagement in auditory/memory tasks. Further T6 analysis could clarify this.

## Coherence: Connectivity Matrix and Connectivity Circle

The connectivity matrices analysed pre- and post-treatment reveal notable patterns in brain connectivity. Prior to treatment, there is widespread, moderate connectivity without well-defined clusters, suggesting a less organised network architecture. Post-treatment, increased connectivity strength is observed, especially in the frontal and central regions for delta, theta, and alpha bands, implying improved communication in crucial cognitive processing areas. Delta band exhibits the most significant increase, indicating enhanced communication for low-frequency cognitive processes. Although beta band connectivity remains widespread, there is some strengthening in frontal and central regions post-treatment. Notable changes include the emergence of stronger connections in frontal and central regions, suggesting improved integration and communication, particularly in delta, theta, and alpha bands. The absence of distinct modules and lateralization implies a distributed network architecture, while increased connectivity strength in specific regions post-treatment suggests improved regulation of cognitive and emotional processes. Short Time Fourier Transform: Representational Similarity Analysis

In our examination, we utilised Representational Similarity Matrix (RSM) to analyse the patterns of EEG recordings before and after treatment. The pre-pre RSM illustrates widespread dark and purple areas, indicating high dissimilarity between pre-treatment EEG recordings among different patients. This suggests a notable variability in the initial brain representations before the intervention. In contrast, the post-post RSM displays an increased presence of red, yellow, and light colours, implying higher overall similarity between post-treatment EEG recordings across different patients. This observation suggests that the treatment may have induced a more convergent and homogeneous pattern of brain activity across the subjects. The pre-post RSM, characterised by a mix of dark, red, and light colours, unveils variability in the treatment's effects on patient similarity. Some pairs of patients exhibit increased similarity, potentially reflecting positive treatment effects, while others remain dissimilar, indicating individual differences in response to the treatment. These findings underscore the nuanced and varied impact of the treatment on the neural representations among individuals.

## Canonical Correlation Analysis: Frequency Bands Power

Canonical correlation analysis of EEG data before and after treatment revealed significant insights. The prevalence of red and orange areas indicated widespread positive correlations between pre and post-treatment band powers, suggesting enhanced consistency and potential synchronisation in brain activity patterns across various frequency bands and regions post-treatment. Conversely, blue and purple areas hinted at less frequent negative correlations, suggesting enhanced differentiation or specialisation in specific brain regions and frequency bands following treatment. Further examination highlighted strong positive correlations in frontal and parietal lobes, indicative of improved functional integration and coordinated activity post-treatment, potentially contributing to enhanced cognitive control, attention, and information processing. Notably, the delta and beta bands showed stronger positive correlations, potentially reflecting a focus on fundamental cognitive processes in frontal and parietal lobes. In contrast, the theta and alpha bands exhibited more mixed patterns, suggesting a broader impact on diverse cognitive functions. Specific brain regions, such as central electrode Cz, displayed enhanced information processing and integration. Temporal lobes exhibited mixed patterns, particularly in theta and alpha bands, indicating nuanced changes in emotional processing and auditory functions. These findings suggest potential implications for cognitive function, with widespread positive correlations supporting improved cognitive control post-treatment, while mixed patterns in temporal lobes suggest individualised impacts. Further validation through clinical data and specific cognitive tasks is recommended for a precise understanding of the observed canonical correlation analysis patterns.

## Canonical Correlation Analysis: EEG MultiChannel Time Series

The comprehensive analysis of overall network connectivity reveals a moderately interconnected system characterised by a prevalence of yellow and orange hues, indicating generally positive correlations between components. This suggests a balanced network with a blend of integration and differentiation, as evidenced by both positive and negative correlations within the system. Notably, distinct clusters with strong positive correlations point to central hubs that likely serve as information-sharing centres, hinting at a potentially modular organisation where closely connected groups of components form distinct subnetworks. The overall pattern exhibits relative symmetry, indicating balanced interactions and information flow across the network, while subtle asymmetries in correlation strength suggest potential specialisations or unique information-processing roles for specific components or subnetworks. In summary, the correlation patterns imply a well-organised network with efficient information transfer, a balance between overall integration and specialised functions, and the presence of central hubs and potential modularity, facilitating efficient information sharing within subnetworks and coordinated activity across the larger system.

The post-retreat analysis of overall network connectivity indicates substantial changes compared to the pre-retreat condition. The post-retreat Y-weight heatmap reveals an augmented strength of positive correlations, marked by increased red and orange hues, suggesting potential improvements in integration and synchronisation within the brain network following the retreat. A reduction in blue and purple areas in the post-retreat data implies a decrease in negative correlations, potentially indicating reduced functional differentiation and more unified activity across the network. High connectivity clusters, particularly central hubs, persist post-retreat, emphasising their ongoing role in information sharing and network coordination. These clusters appear more well-defined and spatially distinct in the post-retreat Y-weight heatmap, suggesting a potentially reinforced modularity within the network, indicative of more efficient and specialised processing within subnetworks. Enhanced symmetry in interhemispheric communication in the post-retreat data suggests improved communication and balanced information flow between hemispheres. Subtle asymmetries observed in the pre-retreat data become less apparent post-retreat, possibly indicating a greater balance in specialised functions across hemispheres. Inferences from the post-retreat data suggest a brain network with heightened interconnectedness, synchronised activity, potentially improved information flow, and a potentially sharper modularization.

# List Of Figures

## Figure S1 : Distribution of EEG recording durations (in seconds) for pre-retreat and post-retreat sessions after trimming the first and last 15 seconds.


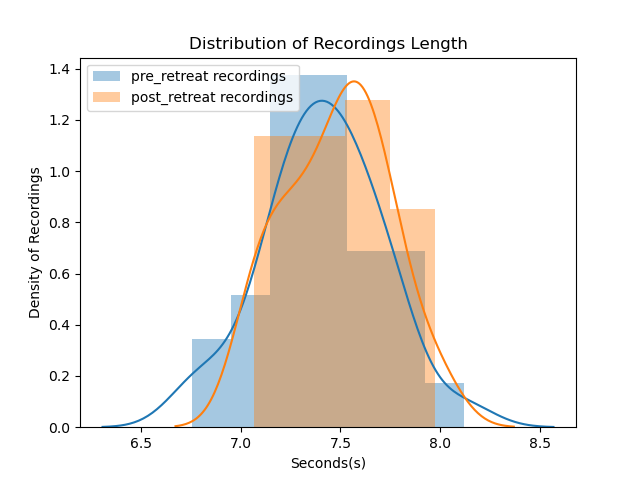


## Figure S2 : Histogram showing reported line frequency of EEG recordings, confirming all recordings reported zero line frequency.


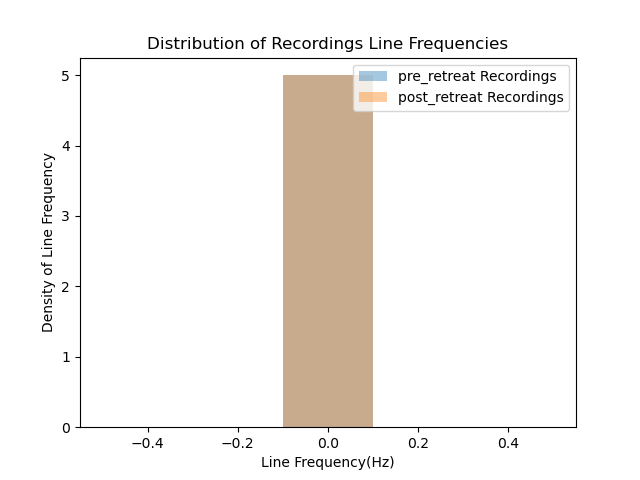


## Figure S3 : Histogram depicting the sampling frequency (Hz) of all EEG recordings, highlighting consistent sampling frequency (300 Hz) across recordings and variations in original bandpass filter settings.


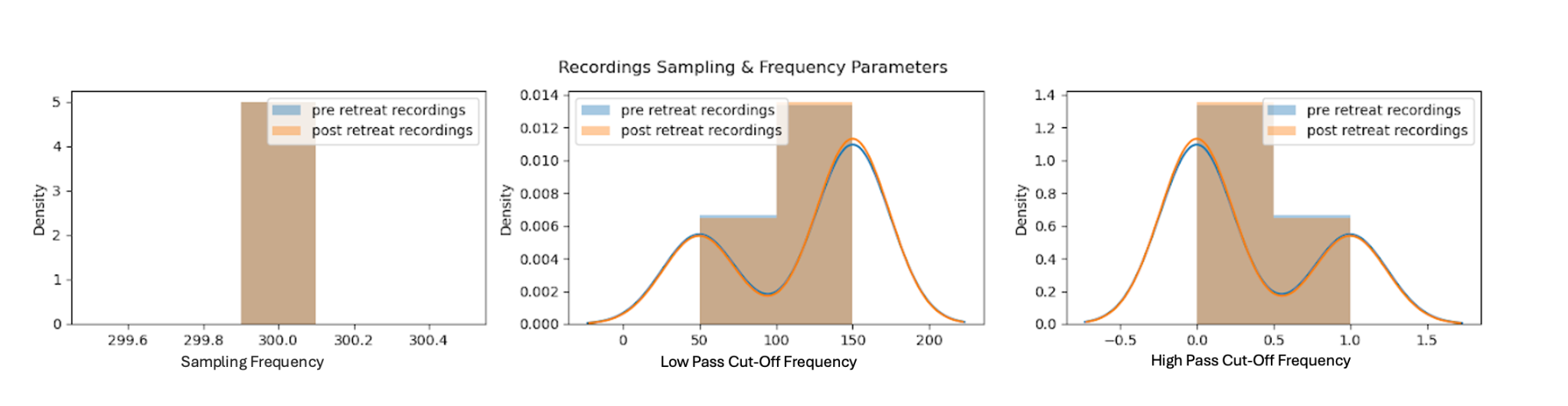


## Figure S4a, b, c, and d: Power spectral density plots illustrating EEG data before and after bandpass filtering (1–35 Hz). Panels 4a and 4b show pre-filtering PSD with evident 50 Hz noise and harmonics. Panels 4c and 4d display post-filtering PSD, indicating effective noise removal.


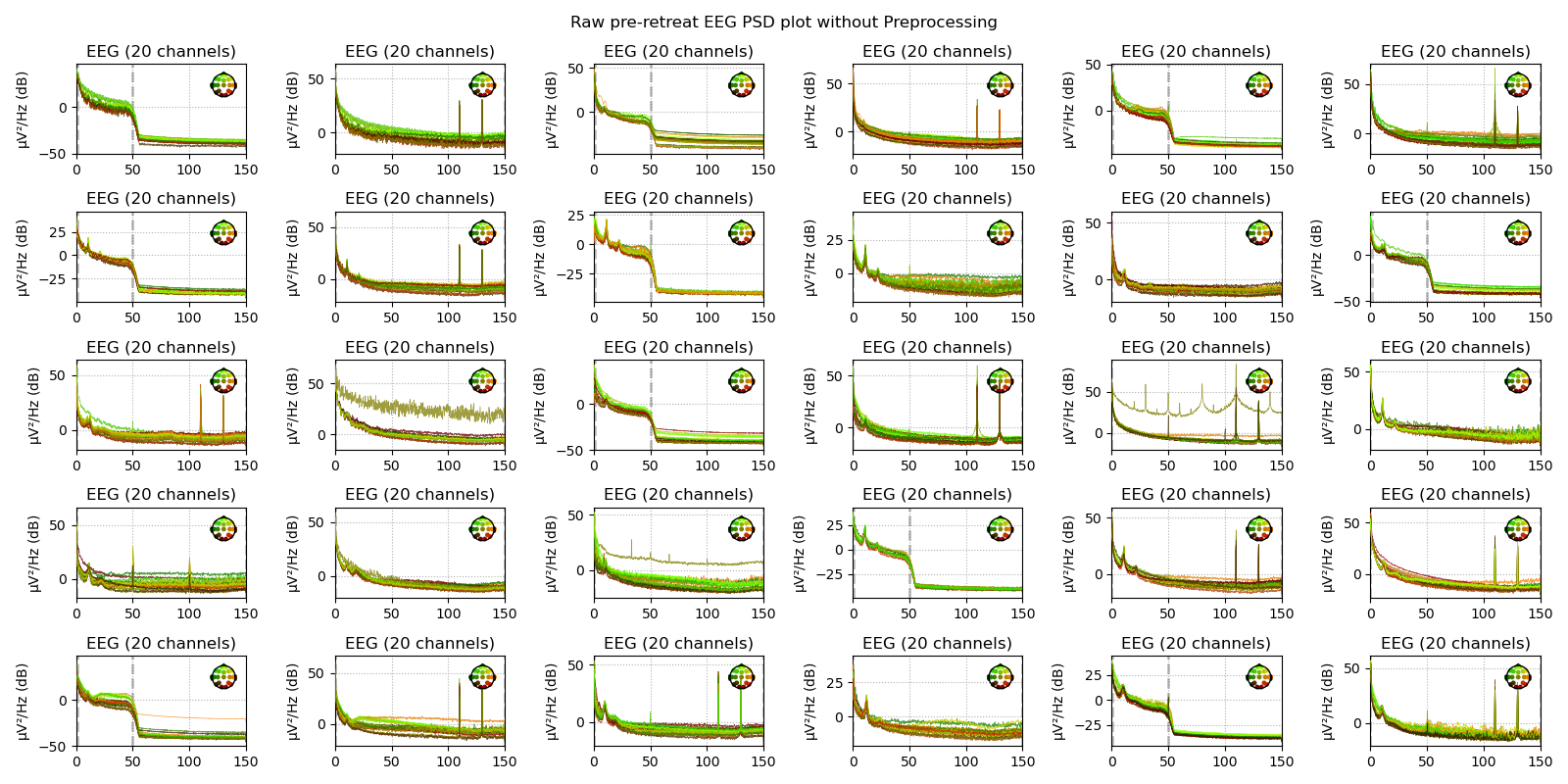


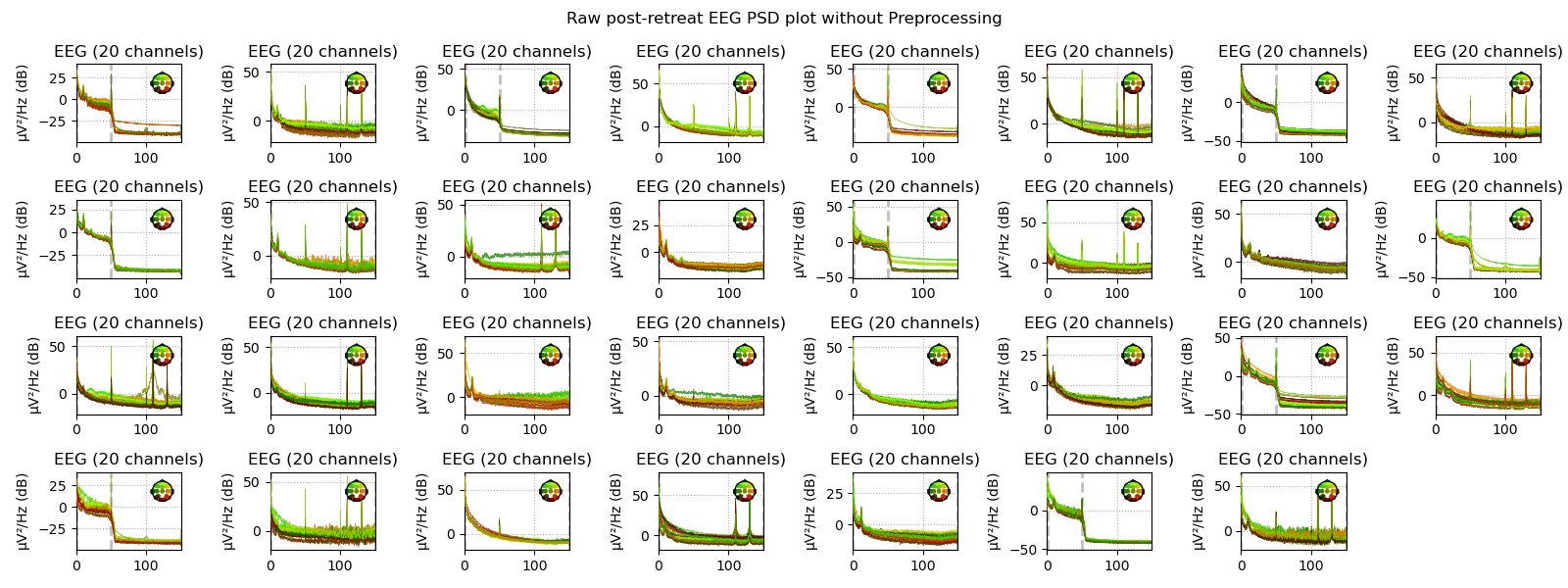

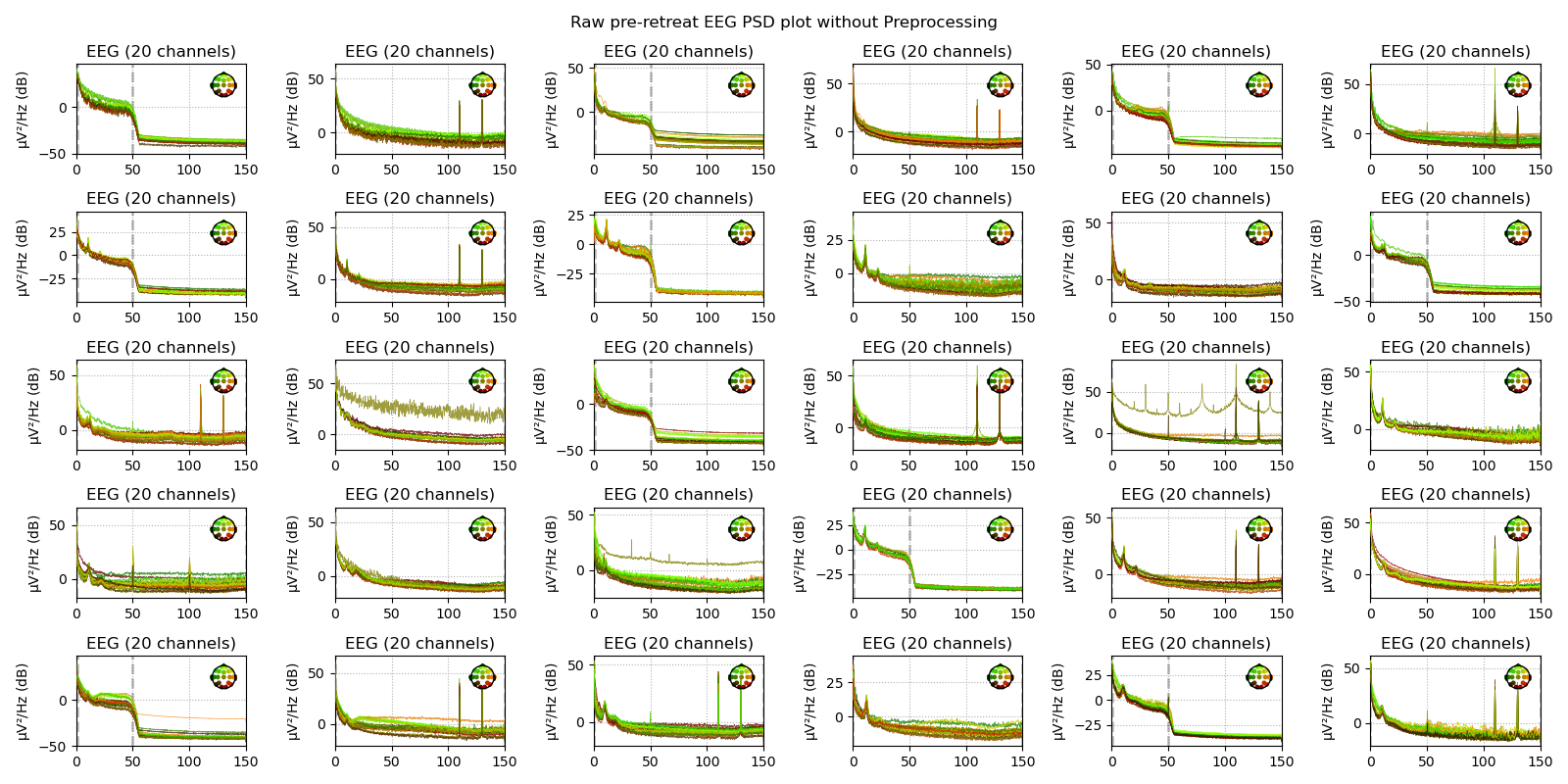

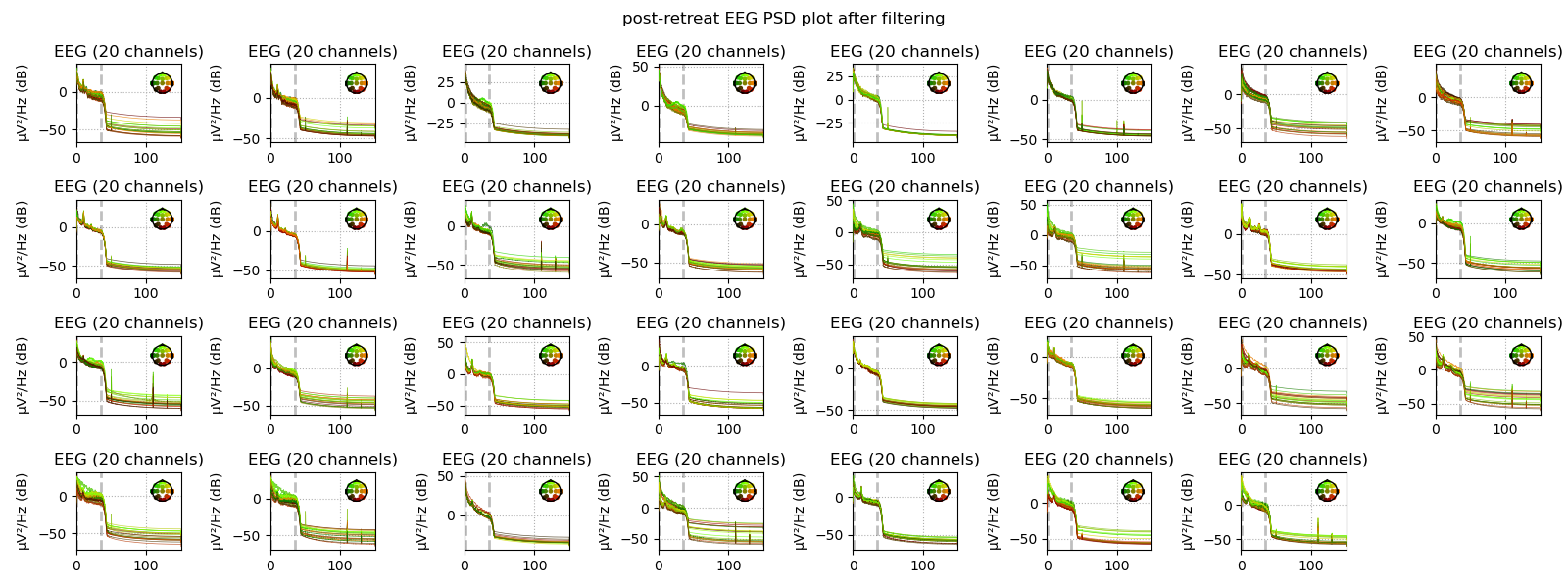


## Figure S5: Internal correlation matrices of EEG frequency bands' power (delta, theta, alpha, beta) in pre- and post-retreat recordings, highlighting significant changes, particularly in delta band correlations.


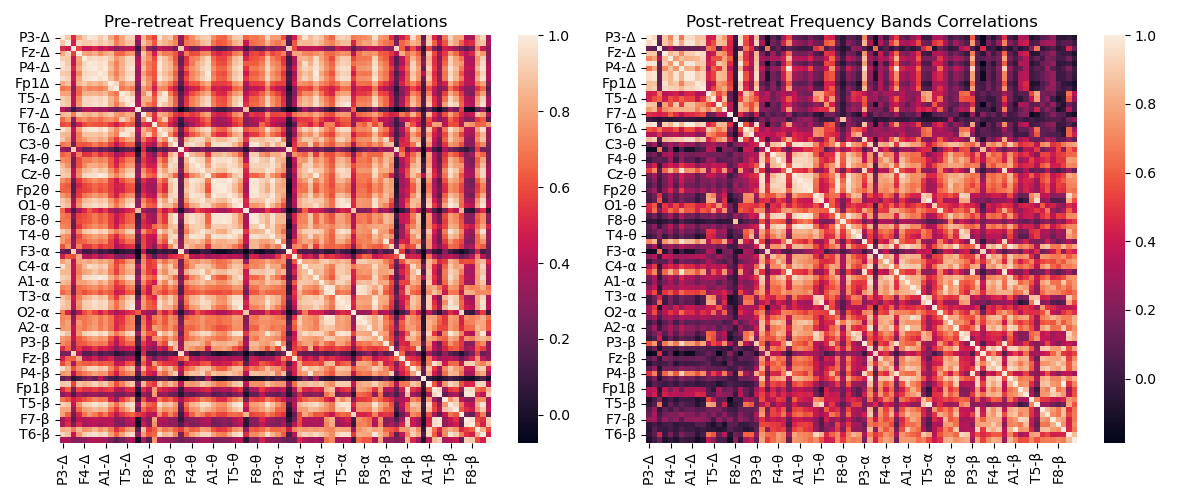


## Figure S6: Canonical correlation analysis (CCA) weights showing significant shifts in EEG frequency band relationships between pre-retreat (independent variables) and post-retreat (dependent variables), highlighting the notable changes in delta band weights.


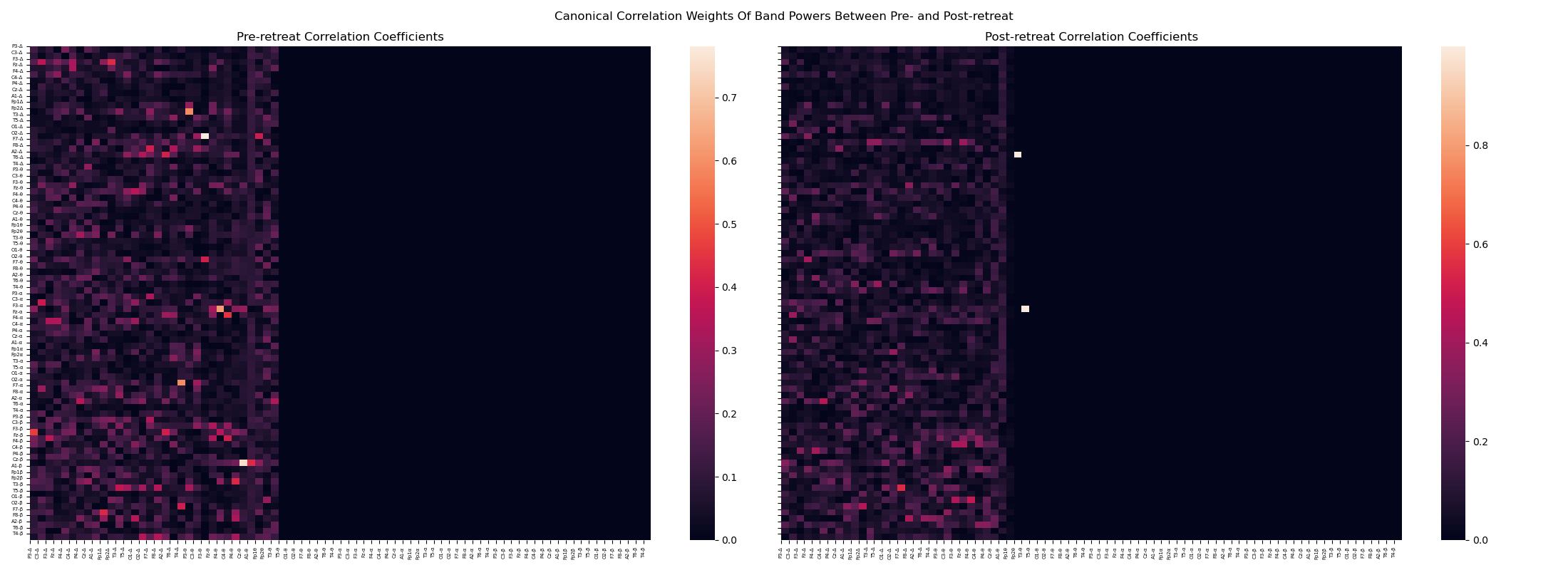


## Figure S7: Montage visualizations depicting differences in EEG bands' power between pre-retreat and post-retreat recordings, illustrating improvements following treatment.


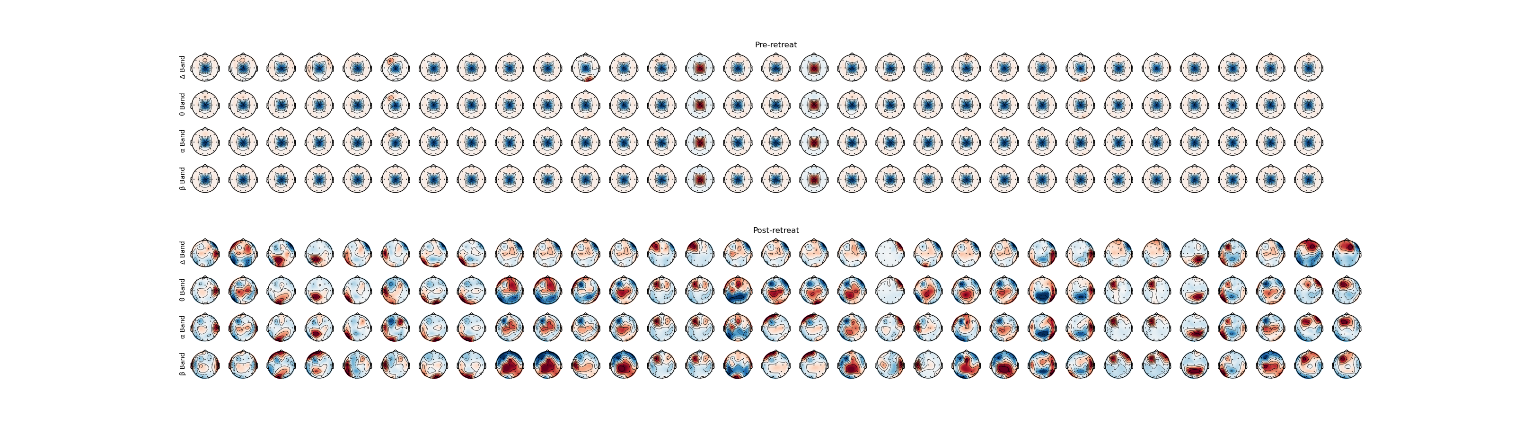


## Figure S8a, b, c, and d: Distributions of the logarithmic EEG band power (delta, theta, alpha, and beta) comparing pre-retreat and post-retreat groups, highlighting notable shifts toward treatment-induced normalization.


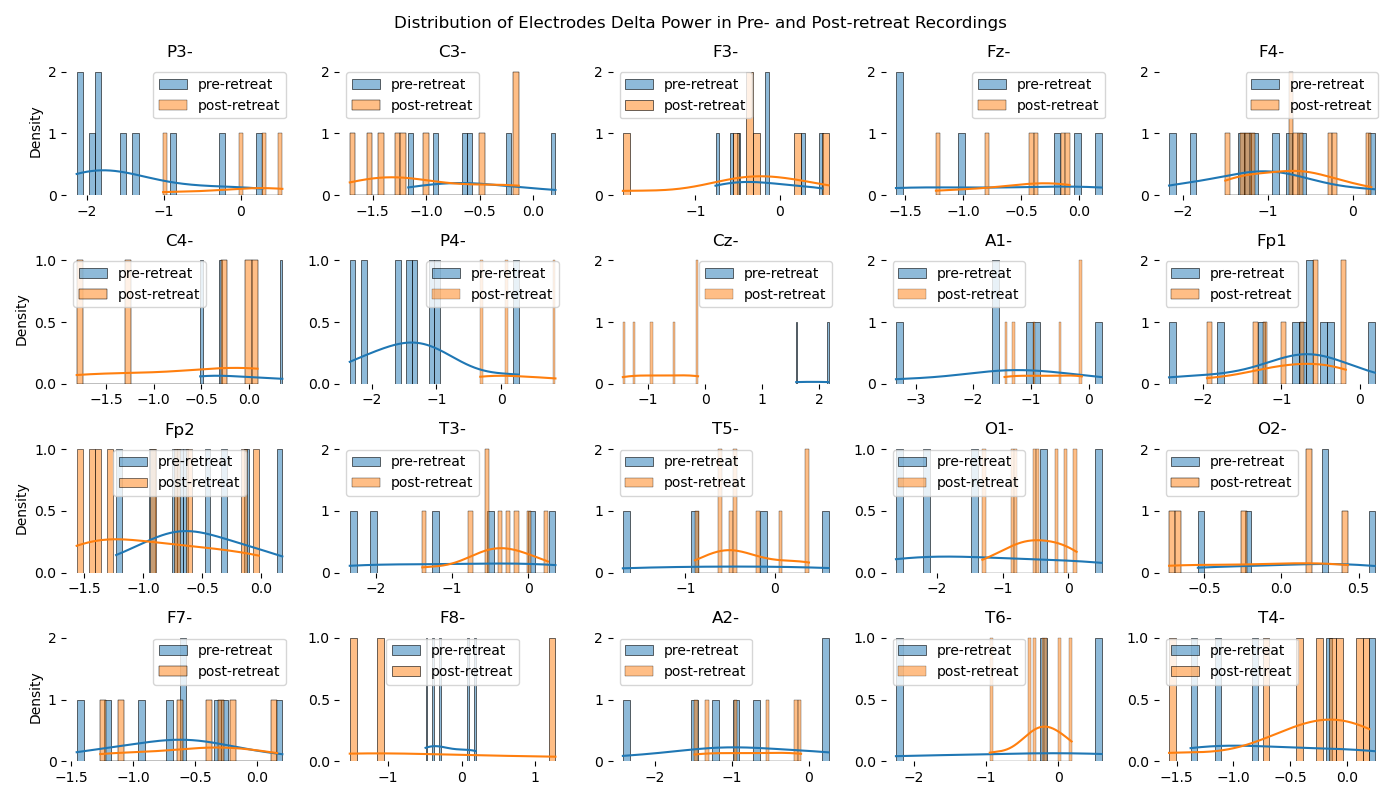


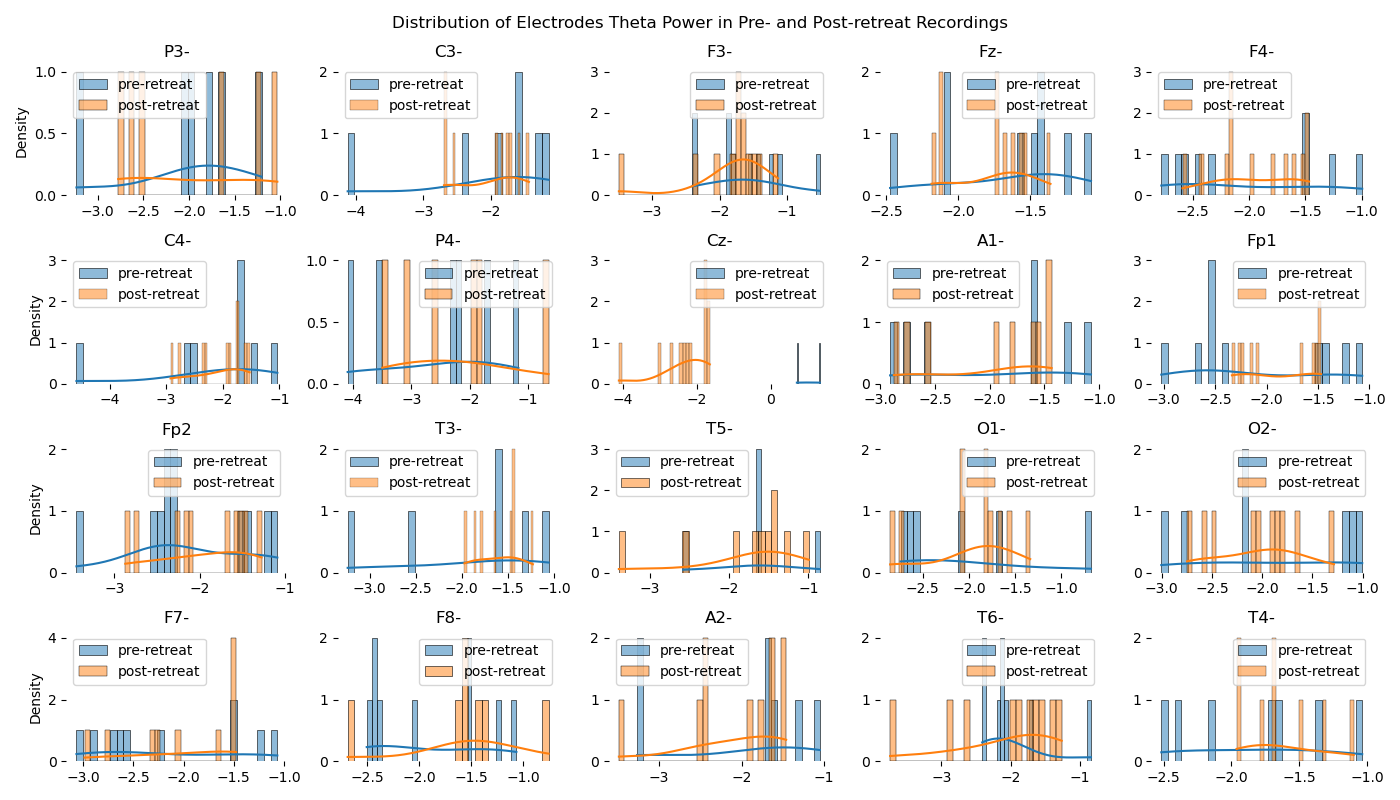


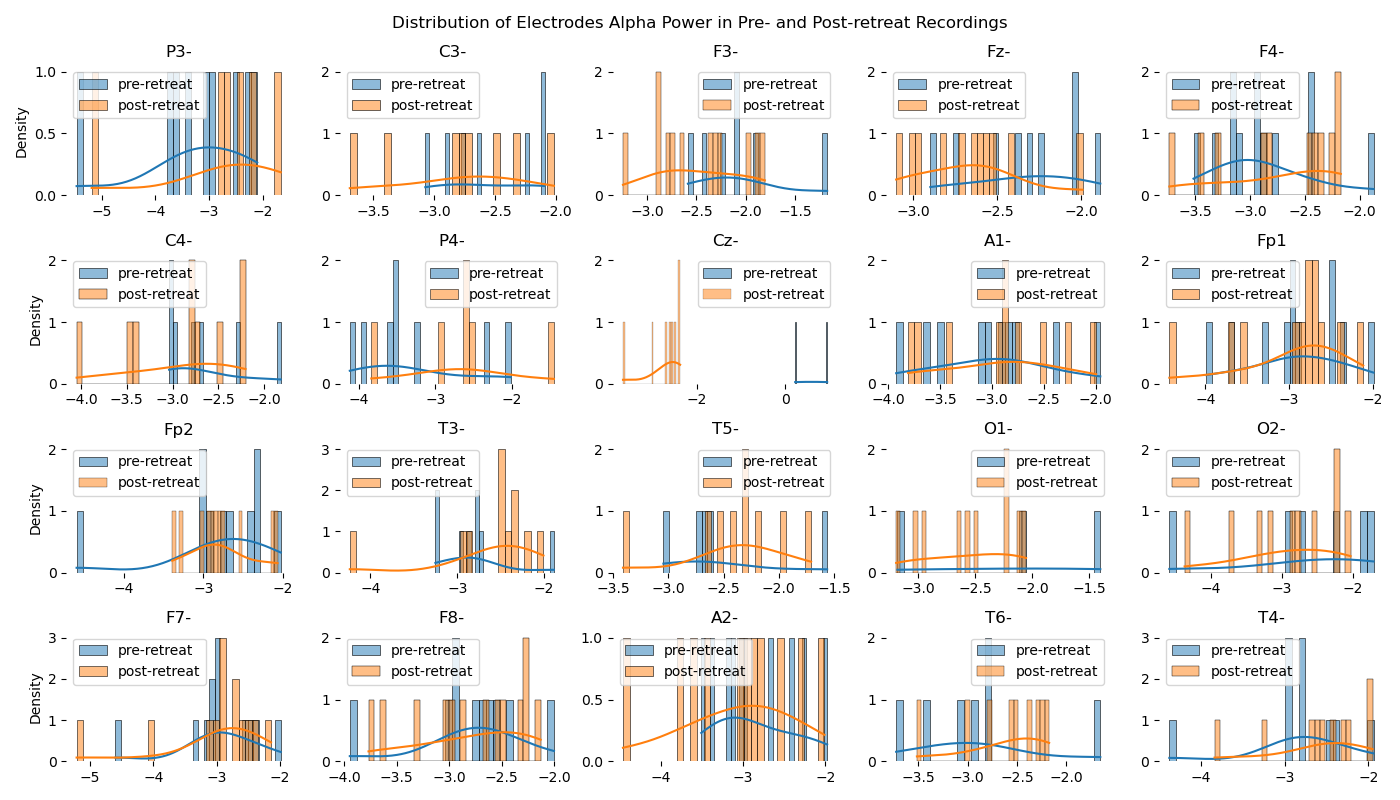


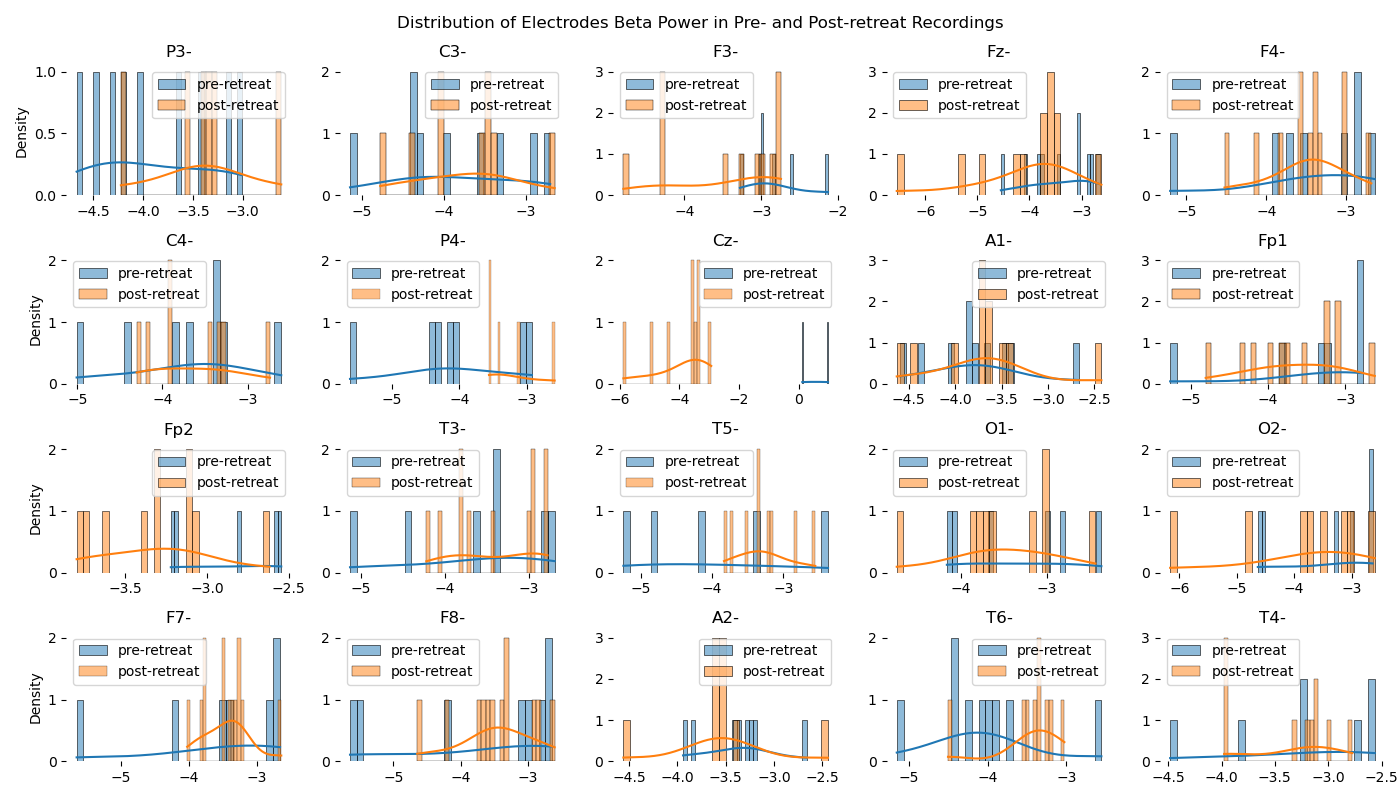


## Figure S9: Heatmaps displaying averaged EEG coherence (scaled 0–1) across delta, theta, alpha, and beta bands, comparing pre- and post-retreat sessions, reflecting changes in connectivity patterns.


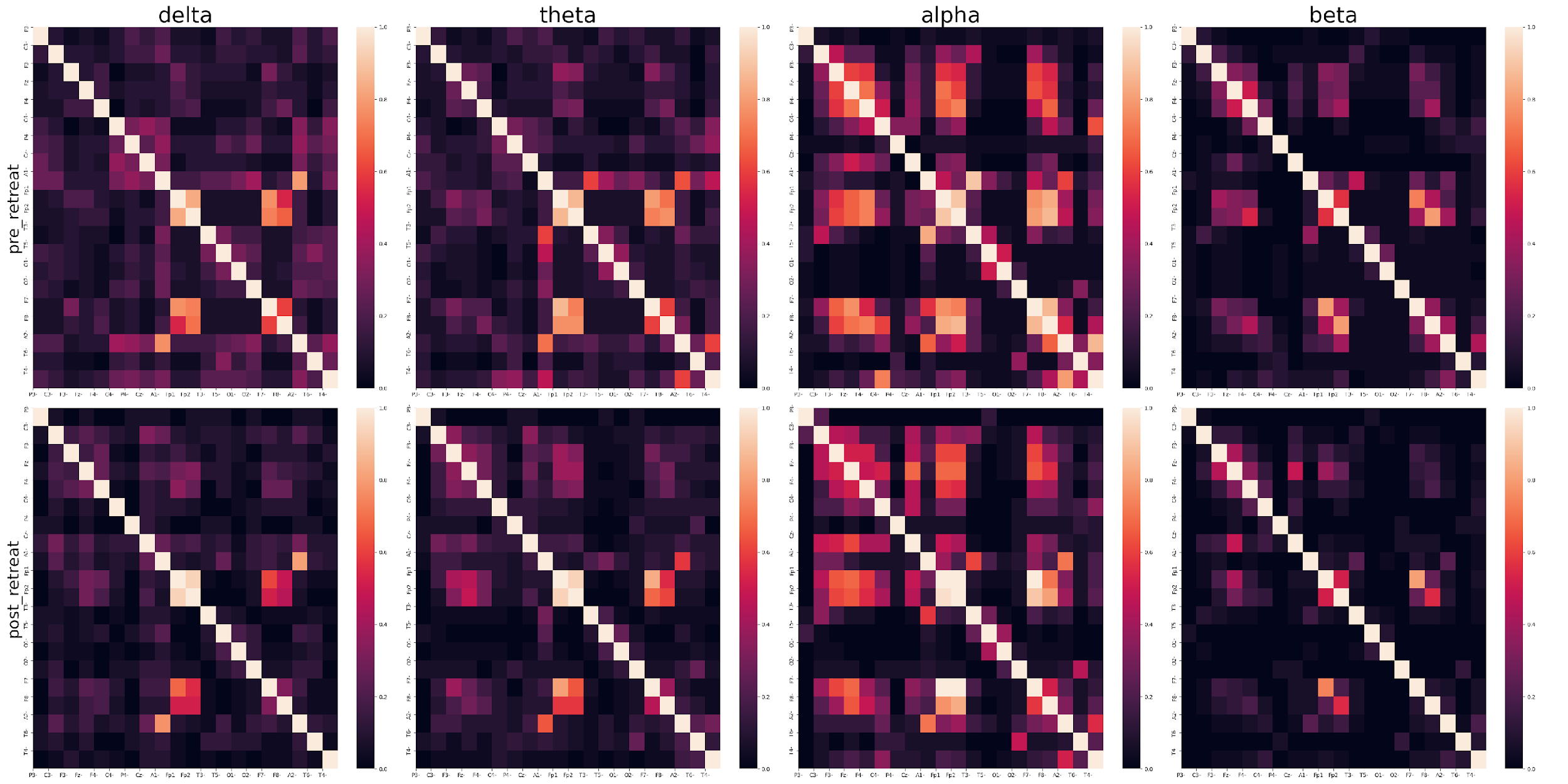


## Figure S10: Connectivity circles illustrating electrode coherence changes across EEG frequency bands (delta, theta, alpha, beta) from pre- to post-retreat, indicating enhanced synchronization post-treatment.


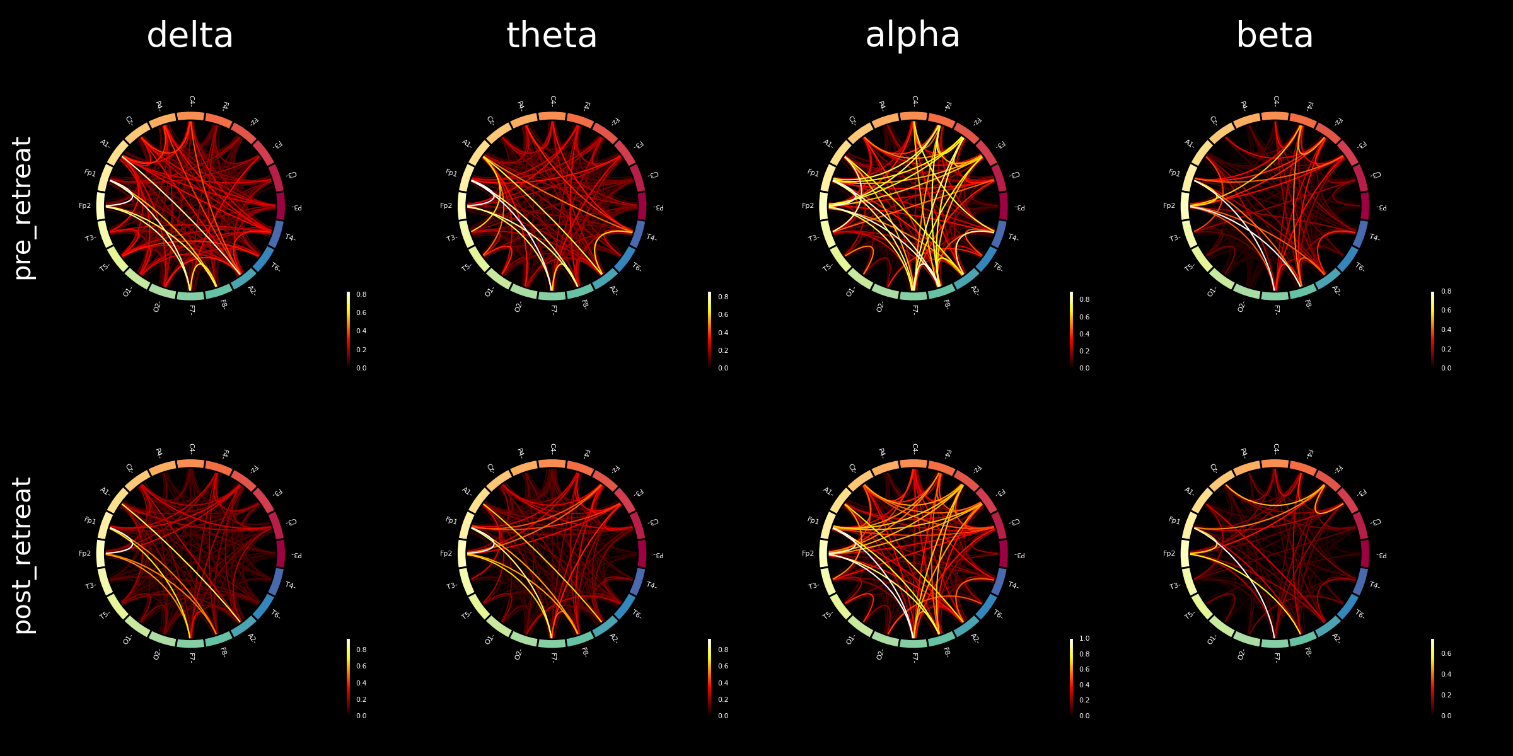


## Figure S11: Representational Similarity Matrices (RSM) derived from Short-Time Fourier Transform spectrograms comparing pre-retreat vs. pre-retreat, post-retreat vs. post-retreat, and pre-retreat vs. post-retreat EEG recordings, showing increased similarity patterns post-treatment.


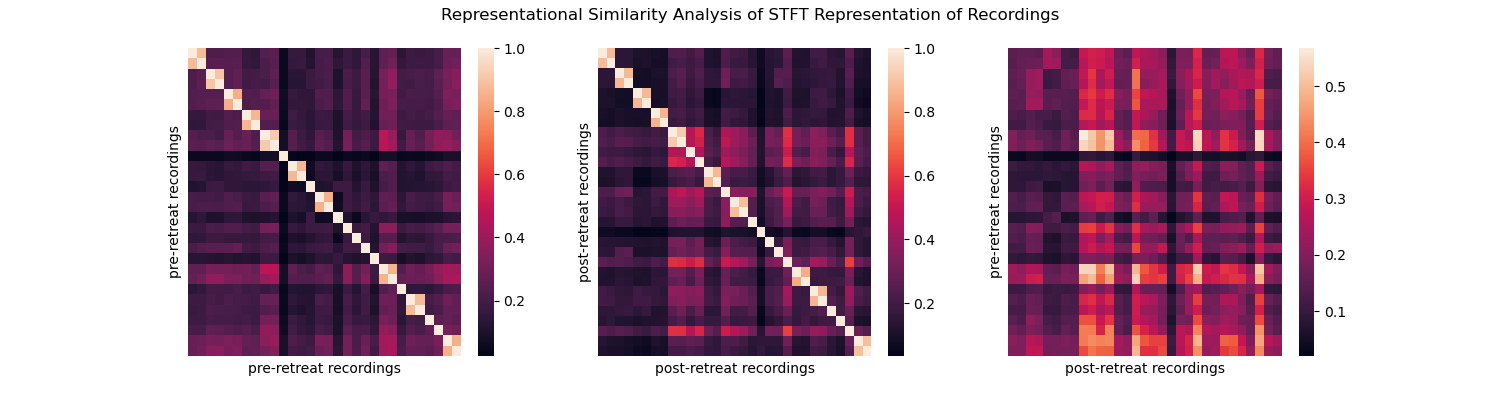


## Figure S12a, and b: Canonical Correlation Analysis (CCA) results comparing multichannel EEG time series pre- and post-retreat. Panel 12a illustrates canonical weights for pre-retreat recordings, and panel 12b presents canonical weights post-retreat, indicating significant shifts toward enhanced network integration after treatment.


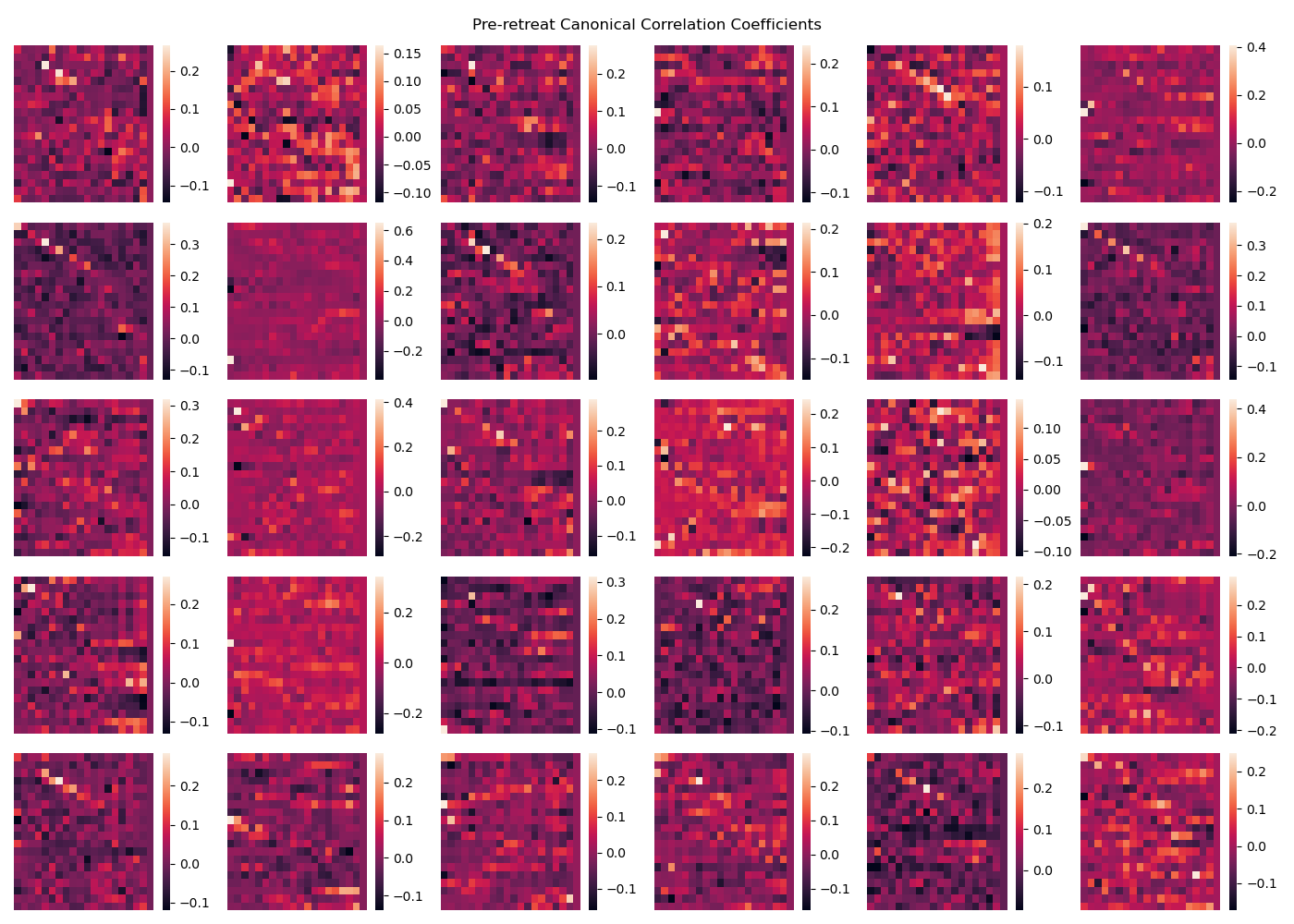


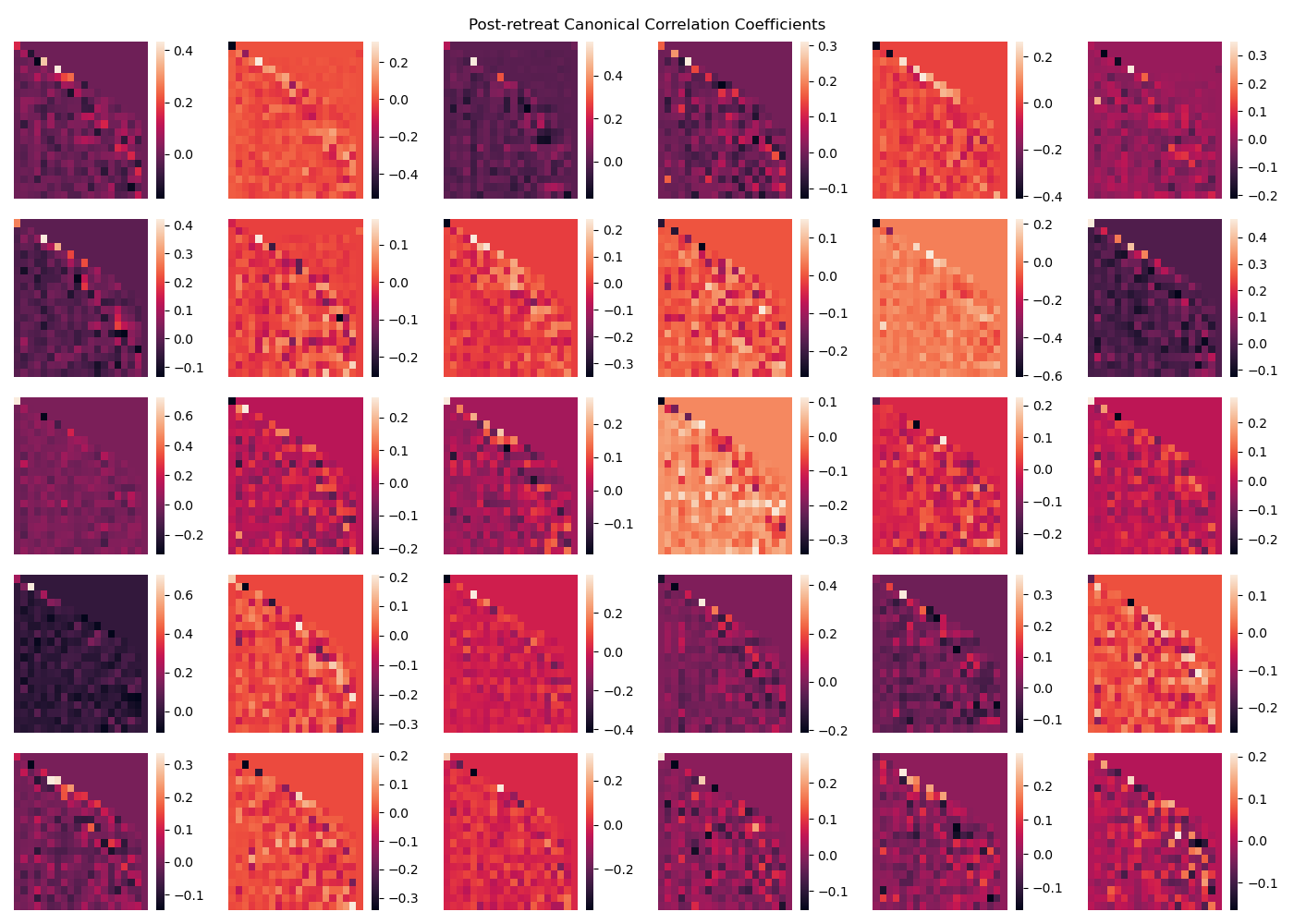


## Figure S13: Barplot of –log₁₀(p-values) for Wilcoxon signed-rank tests comparing pre- and post-treatment spatial variance across EEG bands (delta, theta, alpha, beta).


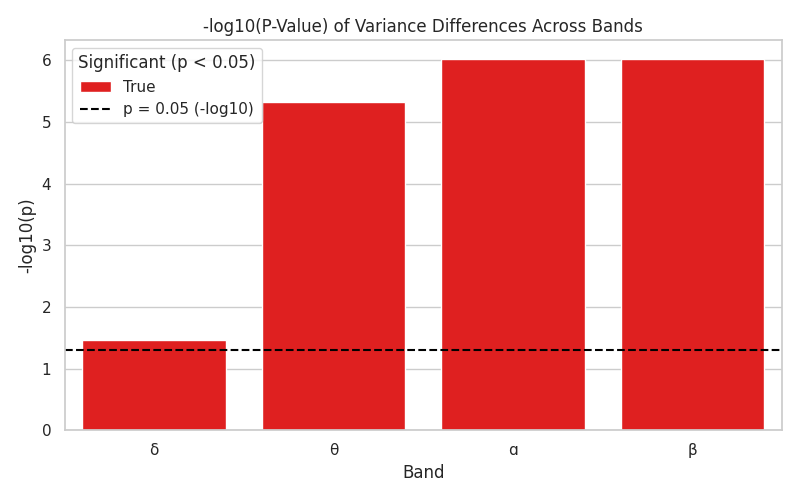


## Figure S14: Violin plots showing the log-scaled distribution of band power variance across channels pre- and post-treatment for each frequency band.


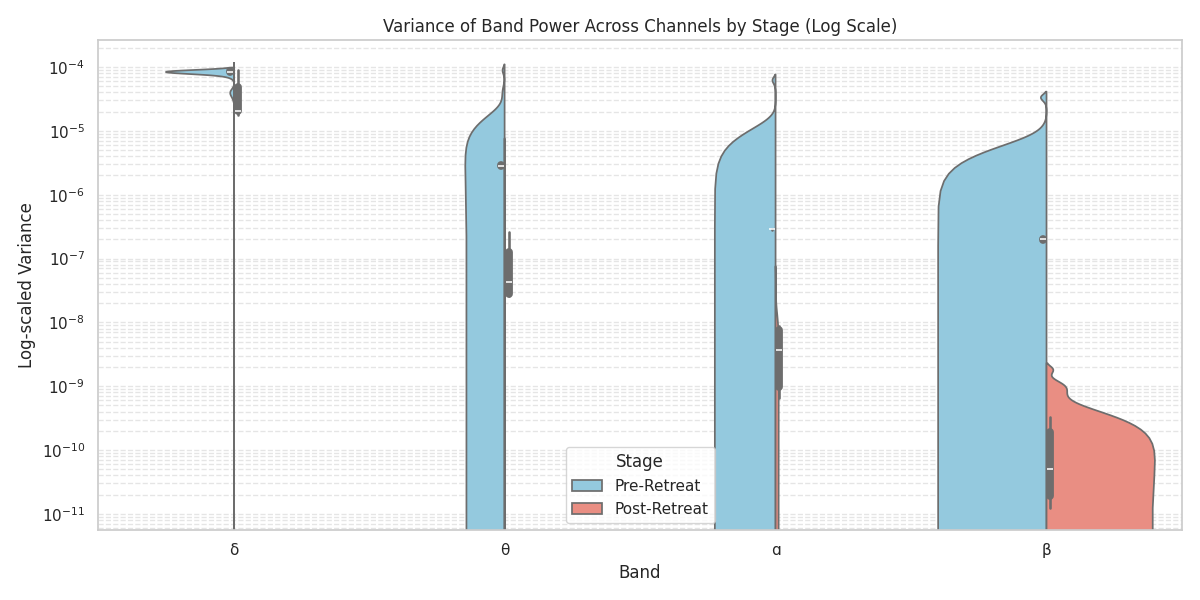


## Figure S15: Electrode-wise statistical comparison of delta vs. alpha band power (–log₁₀ of FDR-corrected p-values), pre- vs. post-retreat.


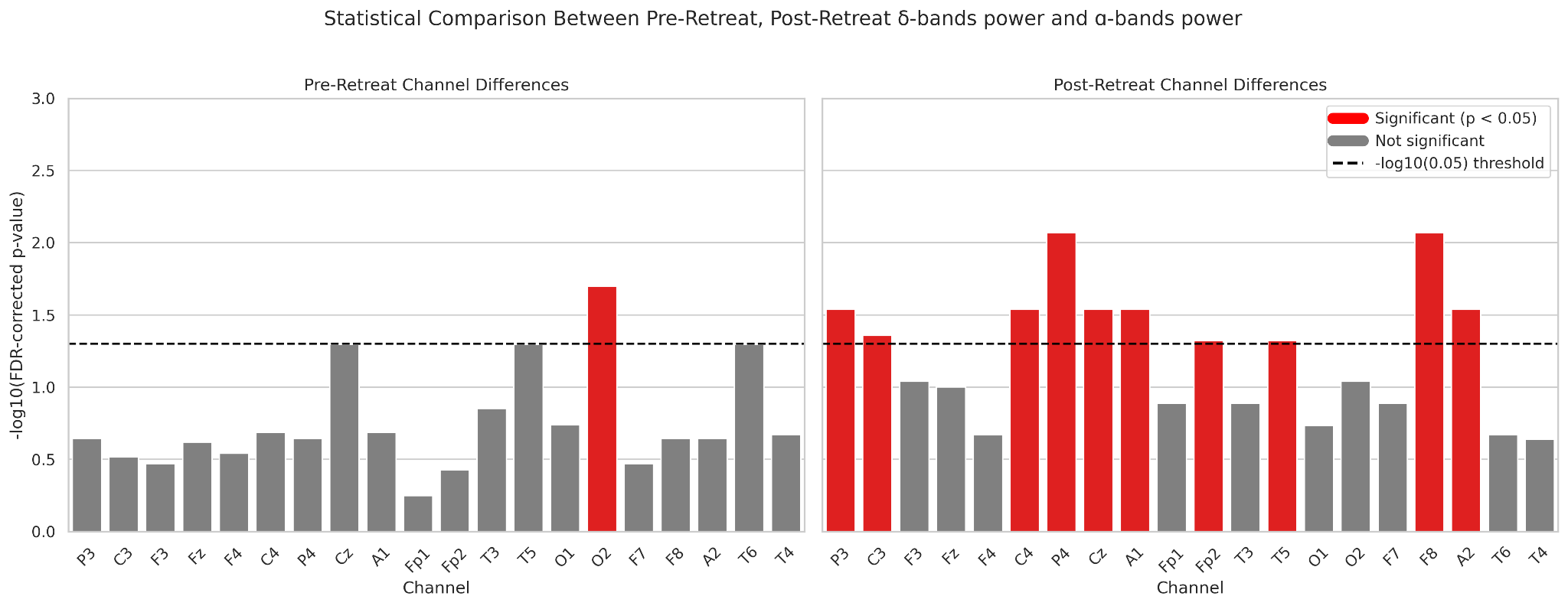


## Figure S16: Electrode-wise statistical comparison of delta vs. beta band power (–log₁₀ of FDR-corrected p-values), pre- vs. post-retreat.


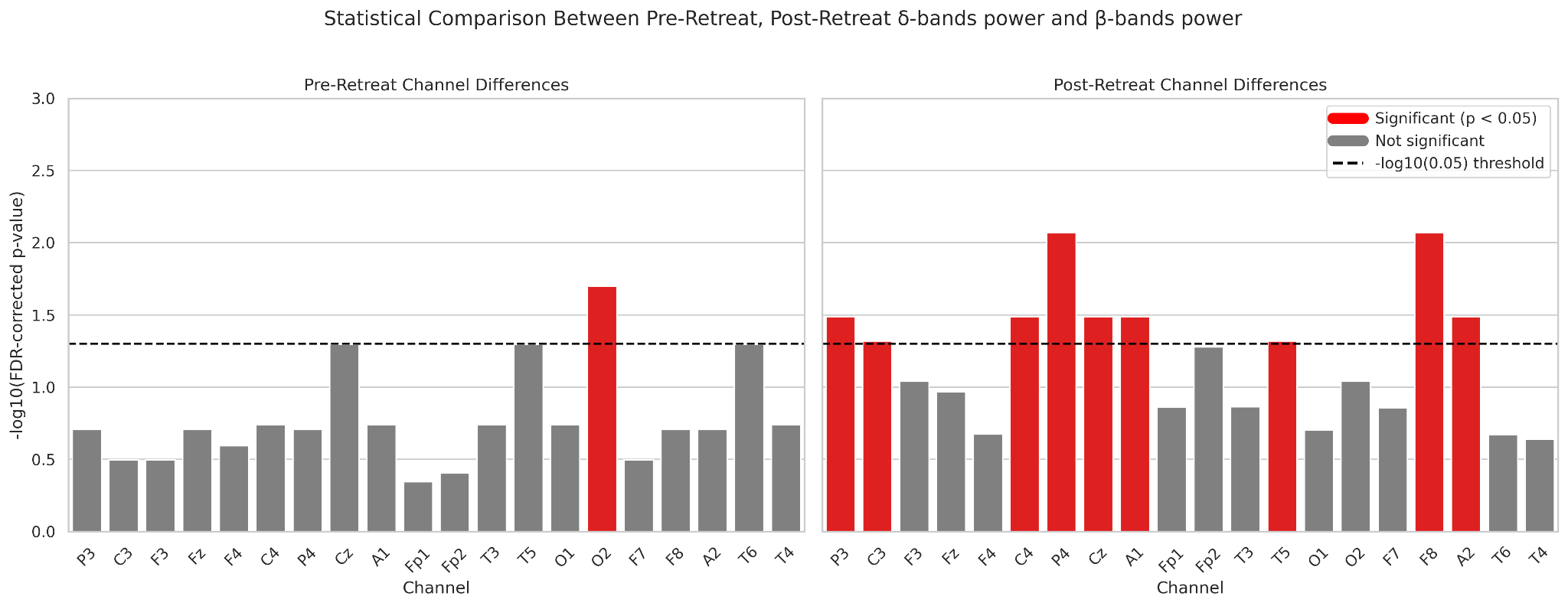


## Figure S17: Electrode-wise statistical comparison of delta vs. theta band power (–log₁₀ of FDR-corrected p-values), pre- vs. post-retreat.


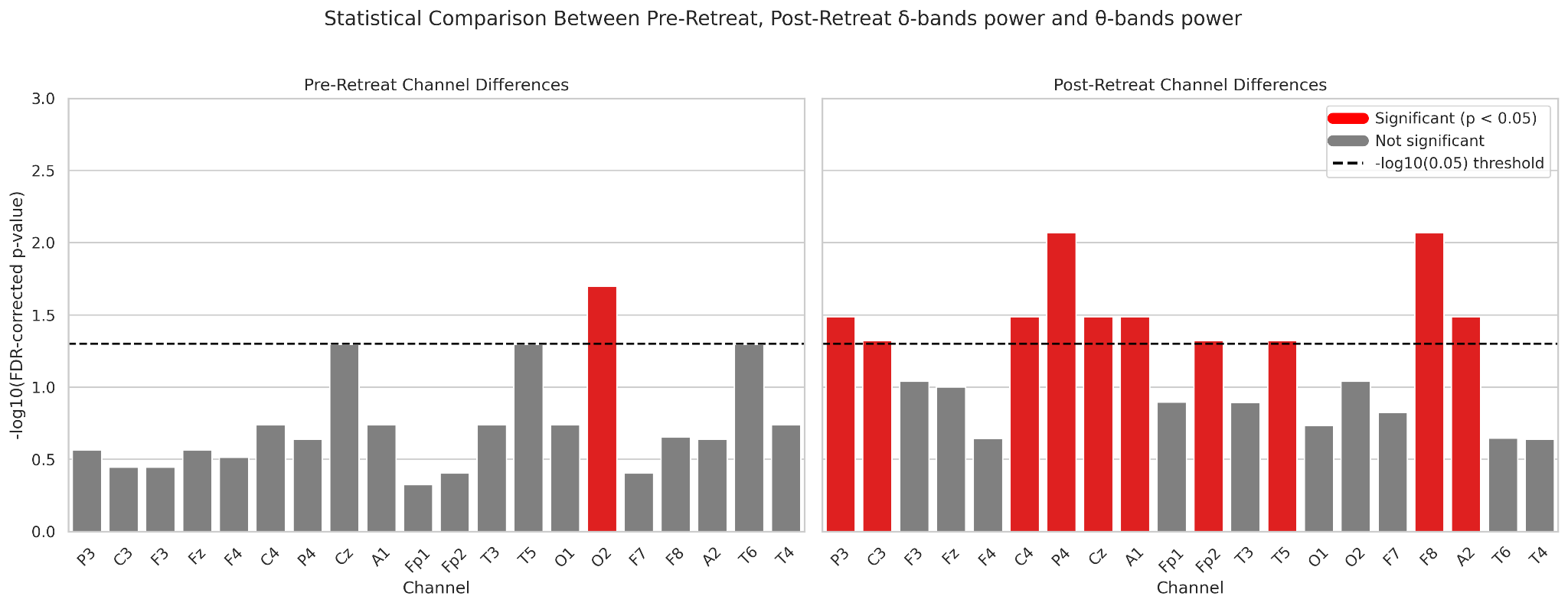


## Figure S18: Electrode-wise statistical comparison of theta vs. alpha band power (–log₁₀ of FDR-corrected p-values), pre- vs. post-retreat.


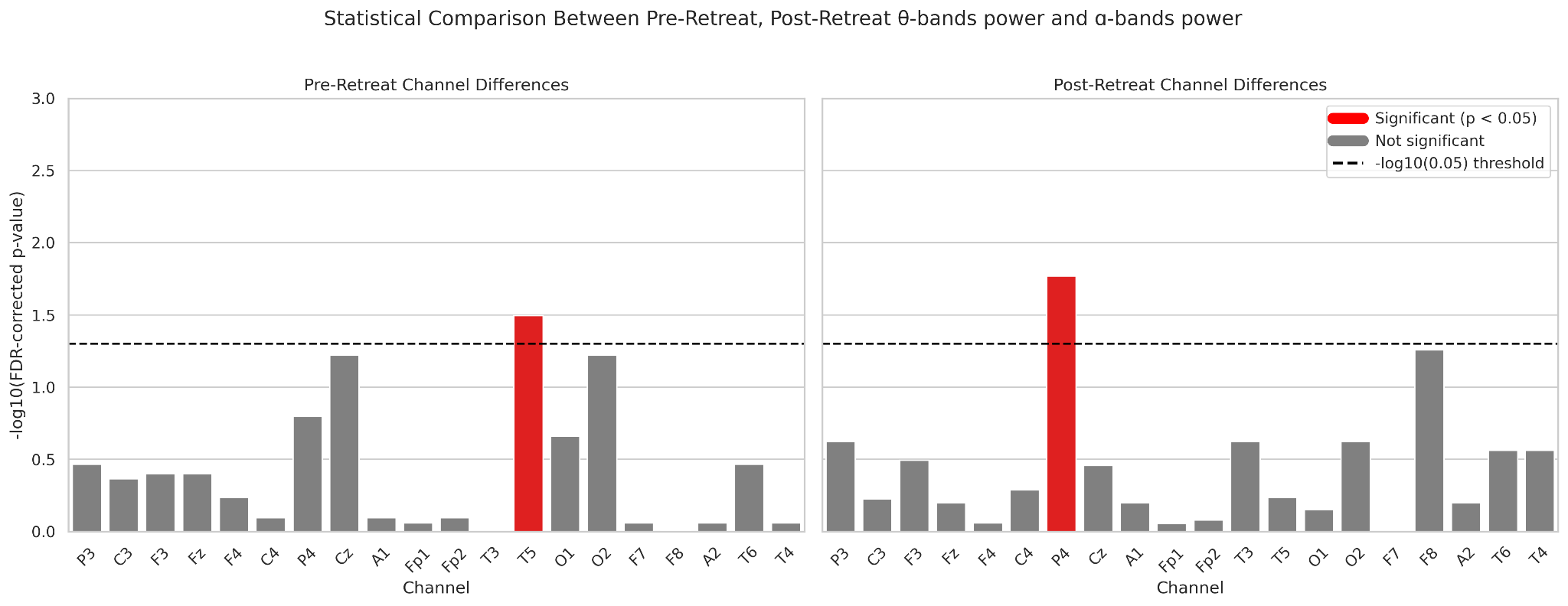

Supplement: Supplementary file 2 [file Supplementaryfile2.docx]
